# Understanding Patient Experience: A Course for Residents

**DOI:** 10.15766/mep_2374-8265.10558

**Published:** 2017-03-22

**Authors:** Julie Niedermier

**Affiliations:** 1 Associate Professor, Department of Psychiatry, The Ohio State University

**Keywords:** Safety, Patient Satisfaction, Patient Experience, Quality of Care, Clinical Effectiveness

## Abstract

**Introduction:**

A 4-hour curriculum was developed to provide residents with information about the concepts of patient satisfaction and experience. The course focuses on the competencies of professionalism and interpersonal and communication skills. It is designed to allow participants to reflect on current knowledge of the patient experience and service principles and to develop a greater appreciation of these concepts’ utility and importance to everyday work.

**Methods:**

Thirty-two residents in 2015 and nine incoming residents in 2016 participated in weekly hour-long sessions over 4 weeks. The curriculum also included an optional fifth component, in which patient satisfaction data were provided to the residents. Residents participated in pre- and postcurriculum survey assessments regarding their awareness of concepts involving patient experience.

**Results:**

Preliminary results suggested that residents found the curriculum beneficial and that it helped to increase their understanding of the relevance of patient satisfaction and experience education to their practice. Quarterly feedback from patient surveys was provided to residents, identifying strengths and opportunities for improvement.

**Discussion:**

Given the growing importance and utilization of patient satisfaction surveys, residents participated in this educational intervention to determine if a novel curriculum and proactive approach to resident understanding and utilization of satisfaction data could result in increased patient satisfaction with resident interactions. The study is ongoing and longitudinal, with initial results encouraging.

## Educational Objectives

By the conclusion of this module, the learner will be able to:

1. Describe current evidence, best practices, and controversies involving patient surveying and the role these surveys play in providing patient-centered care.
2. Name specific systems in place at the national level to monitor health care quality.
3. Discriminate between organizational and individual implications of patient satisfaction data.
4. Demonstrate professionalism and interpersonal and effective communications skills that compose service excellence.

## Introduction

Patient satisfaction is a commonly used indicator for measuring physician performance, yet the true significance and relationship between satisfaction results and both actual patient outcomes and quality of care received remain controversial.^1,4^ Patient satisfaction surveys and results have implications for both organizational and individual reputations as well as organizational financial health, while at the same time presenting ethical dilemmas, potential benefits, and inherent threats to the physician-patient relationship.^3^ Despite widespread surveying of patients in teaching hospitals nationwide, residents generally have both limited exposure to and understanding of the significance that patient satisfaction results may have to their postresidency professional careers.^4,4^

In addition, patient satisfaction surveys are imperfect proxy measures of the larger concept of patient-centered care.^6^ The essence of the patient-centered care approach is the experience of shared decision making between patients, families, and caregivers, as well as the quality of these interactions, emphasizing not only satisfaction but also positive clinical outcomes.^7^ Often, for the inherent mutual benefits of success, organizations and training programs undertake widespread efforts to promote patient-centered care and achieve favorable results,^8,4^ while offering suggestions for future directions.^10,4^

Unlike existing resources available in MedEdPORTAL,^12–14^ this resource assesses outcomes of resident communication and professional interaction within actual patient encounters over time. This is in contrast to those using standardized patients. It is the use of actual patient encounters that bridges the gap between classroom simulation skills and clinical practice.

## Methods

### Background

Materials for the course have been derived from existing literature regarding the patient experience, patient-centered care, and patient satisfaction. The course focuses on the integration of professionalism and interpersonal communication skills as they relate to the patient experience.

### Participants

This course has been implemented annually at The Ohio State University Harding Hospital Psychiatry Residency Program for the last 2 years, with a total of 40 participants.

### Logistics

The course is composed of four weekly 1-hour sessions conducted over 4 weeks. An optional fifth session is available for institutions interested in providing residents with specific feedback solicited from actual patients of the residents. The required curricular elements by session are listed below:

• Session 1: precurriculum survey (Appendix A) and the PowerPoint presentation Understanding “Patient Experience” (Appendix B, slides 1–16).
• Session 2: the PowerPoint presentation Understanding Patient Satisfaction and Quality Measures** (Appendix B, slides 17–32).
• Session 3: the PowerPoint presentation Strategies for Improving Patient Satisfaction and the Patient Experience (Appendix B, slides 33–56). The self-evaluation of patient encounters (Appendix C) is needed for this session.
• Session 4: Facilitator questions (Appendix D) are needed to direct a discussion about hospital/provider satisfaction data. It is beneficial to have on hand additional materials such as hospital-specific or physician-specific satisfaction data. These can be acquired from the quality division within the hospital. The postcurriculum survey (Appendix A) is needed here as well.
• Session 5: This is an optional session consisting of feedback provided to trainees individually, based on summary data from patient survey questions (Appendix E). This session occurs at a defined interval (such as 3–6 months) following Sessions 1–4 and consists of individual or ongoing meetings between the instructor and learner.

### Assessment

There are two assessment methods involved in this course, with an optional third assessment method related to Session 5:

• Resident pre- and postcurriculum understanding of concepts is assessed using Appendix A. The pre- and postcurriculum surveys take approximately 10–15 minutes each to complete.
• Residents complete a self-assessment of aspects of patient service using Appendix C. This self-assessment is derived from principles of the customer service industry and should take approximately 10–15 minutes to complete.
• Utilized in the optional fifth session, the patient survey (Appendix E) collects feedback from patients on the residents’ clinical rotations. In the 4 months prior to and following the educational sessions, patients are surveyed about their resident interactions using questions derived from standardized questionnaires commonly employed throughout our hospital. These surveys may be completed by patients in approximately 10 minutes.

These assessment components hold positive consequential validity as they are designed to be formative. Residents participate in the course annually and typically complete Session 5. During the final session, residents are provided with feedback about their patients’ responses to surveys and identify strategies to address opportunities for improvement and build upon existing strengths.

## Results

Residents rated their awareness and understanding of course content significantly greater following Session 4, compared to the presession survey completed at Session 1, on the 20-item, 5-point Likert-scale (1 = *Very Poor,* 5 = *Very Good*) session surveys (Appendix A). After totaling all response scores, the average presession survey score was 68 out of a possible maximum score of 100, while the average postsession score was 89.

Residents reported that completing the self-assessment survey (Appendix C) based on their own patient encounters and reviewing hospital/personal satisfaction data available at our institution's hospital were helpful. The average rating of the self-assessment survey responses (1 = *Rarely,* 3 = *Sometimes,* 5 = *Always*) was 4.2. The average hospital satisfaction data score, using a 5-point Likert scale, was 4.8.

A significant increase in patient satisfaction with resident interactions in key clinical areas, including the ambulatory and inpatient settings, was measured 4 months pre- and postsurvey/course, as per Appendix E. Using a 4-point Likert-type scale for items 1–3 (1 = *Never,* 4 = *Always*) and a 5-point Likert-type scale for items 4, 5, and 7 (1 = *Very Poor,* 5 = *Very Good*), with a maximum score of 27, the average ambulatory patient satisfaction survey change for PGY 3 residents was 6.3 (from 14.4 to 20.7). The average inpatient patient satisfaction survey change for PGY 1 and PGY 2 residents was 4.2 (from 14.9 to 19.1). It should be noted that items 6 and 8 in Appendix E were not assigned scores when compiling these results. Overall, each resident demonstrated an uneven but sustained upward trajectory on the measure of overall patient satisfaction with resident interactions over time. Specifically, the average patient satisfaction score for PGY 2, PGY 3, and PGY 4 residents who attended this course changed from 14.8 to 20.2 over 12 months.

A significant increase in the number of positive verbatim and anecdotal comments from patients about residents on general hospital surveying instruments was noted. Specifically, the average number of positive verbatim comments 4 months before and after the course regarding PGY 3 and PGY 4 residents rose from 9 to 14. Some examples of these comments include the following:

• “Dr. was AWESOME.”
• “Helped me feel less anxious.”
• “Explained my condition and my medications.”

Improvements in individual and collective resident ratings largely mirrored substantive increases in overall patient experiences in our institution. This was despite a small number of deliberate organized initiatives targeting patient satisfaction beyond this residency curriculum intervention. Given this, ratings in the ambulatory physician section increased from the 76th to the 94th percentile over 1 year, while those in the inpatient physician section increased from the 52nd to the 72nd percentile over the same time period.

### Additional Information

All 40 residents completed the surveys and assessments; however, four missed one of the educational sessions but completed the surveys/assessments following online review of the content. Given that there were often multiple individuals involved in a patient's care, patients received surveys either electronically or from an administrative worker immediately after the clinical encounter. These communications included a picture of and information about the resident to help ensure that patients were indeed providing an evaluation of the appropriate resident. The data regarding verbatim and anecdotal comments utilized textual analysis with count data from open-text comments from patient surveys.

## Discussion

The development and implementation of course materials for Sessions 1–4 were relatively straightforward, allowing for ample opportunities to educate learners and encourage them to reflect on the importance of clinical effectiveness, safety and quality, and the patient experience. The most challenging aspect of the course was providing context and meaning to the residency experience. This intervention required a considerable investment of time initially to develop a mechanism for obtaining satisfaction data about residents in Session 5 compared to other providers. Once a systematic process was established, a usable database of return rate and feedback from patient surveys became readily available for ongoing use.

### Limitations

Speakers need to review the PowerPoint presentation in advance to become familiar with the content, as there may be course instructors uncomfortable with the material. In the outline format of the PowerPoint presentation, additional notes are available to aid speakers’ understanding.

Participation in Session 4 could be variable depending on available hospital data and learner comfort with discussion of these potentially sensitive topics. Learners may receive delicate, disappointing, or overtly negative feedback from patients that can undermine their confidence, yet these experiences typically promote growth of their reflection, communication, and professionalism skills. It is important for instructors to have reviewed the patient survey results in advance and to offer support to learners reacting to criticism.

The survey instruments themselves were adapted from existing resources and may or may not have validity to resident physicians. The surveys may or may not translate to physician experiences within the clinical environment.

Another limitation is the lack of historical control to measure the maturation effect of patient satisfaction as residents advance further in their training. Because participants were at different stages, baseline communication skills were variable for each resident, limiting the utility of aggregate data. Similarly, residents moved among rotational settings that differed during the assessment period, and the return rate of patient responses was variable.

Finally, an additional limitation is potential bias by patient survey responders to attribute possible confounding variables to their residents. Such confounding variables may have included patient interactions with other hospital staff and hospital-related events outside the residents’ control.

### Lessons Learned

Residents were highly engaged in the process, appreciative of the course and its self-assessment components, and receptive to comments from their patients. In many ways, the curriculum became self-sustaining. That is, the more feedback residents received from patients, the more they reflected and the more information from patients (positive or negative) they sought. Providing feedback to residents also offered an opportunity to develop a global understanding of the patient experience, compared to the more traditional approach of reviewing isolated incidents of patient complaints. Overall, residents became less defensive about such feedback and were better able to counterbalance factors contributing to a patient's concern.

This course has been successfully deployed for the last 2 years and is occurring at a time when literature in this field is increasing at a dramatic rate. Understanding the breadth of content necessary to deliver the course effectively could be a challenge for instructors. The PowerPoint presentation describes some specific quality measures currently in use; however, the content is anchored by general principles common to the triad of clinical effectiveness, patient safety and quality, and patient experience.

### Conclusions

Arguably, trainees in a psychiatry residency, relative to other disciplines, may have a higher aptitude for communication skill building that contributed to the success of the intervention. However, any residency has some variability among learners, and all training levels that participated in our intervention made respectable improvements relative to their own skill levels. The curriculum itself is based on all-purpose principles of the patient experience and thus could potentially be generalizable to learners beyond psychiatry residency programs.

This relatively low-cost, high-yield curricular effort is already reaping organizational and personal dividends. It provides a tangible addition to our repertoire of assessment strategies and offers a sustainable process to monitor and provide feedback to trainees during the longitudinal residency experience. Participants refine their communication skills and professionalism, demonstrate an evolving capacity for self-reflection, and assimilate a new domain of vital practice-based skills.

Aspects of the course are being adopted within our institution by nurse practitioners and other disciplines to provide a framework for understanding the patient experience outside the resident learner category. Future opportunities include continuing to provide information to residents about their practice habits and determining the degree to which resident performance can be enhanced with additional experience and feedback.

## Appendices

A. Pre- and Postsession Survey.docx
B. Understanding the Patient Experience Presentation.pptx
C. Self-Assessment of Patient Encounters.docx
D. Facilitator Questions.docx
E. Patient Survey Questions.docx All appendices are peer reviewed as integral parts of the Original Publication.
