# Supplementary material for: Understanding Patient Experience: A Course for Residents
Source: MedEdPORTAL. 2017 Mar 22;13:10558. doi: 10.15766/mep_2374-8265.10558 (PMC6342057; doi:10.15766/mep_2374-8265.10558)
Supplement: Supplementary file 1 — A. Pre- and Postsession Survey.docx B. Understanding the Patient Experience Presentation.pptx C. Self-Assessment of Patient Encounters.docx D. Facilitator Questions.docx E. Patient Survey Questions.docx [file mep-13-10558-s001.zip › A. Pre- and Postsession Survey.docx]

| Please rate your degree of understanding of the following issues: | Very Poor | Poor | Average | Good | Very Good |
| --- | --- | --- | --- | --- | --- |
| 1. The meaning of ‘patient satisfaction’. |  |  |  |  |  |
| 2. The meaning of ‘patient experience’. |  |  |  |  |  |
| 3. The ‘patient experience’, quality and safety, and clinical effectiveness are closely related concepts. |  |  |  |  |  |
| 4. ‘Patient experience’ correlates with the physician’s interpersonal skills. |  |  |  |  |  |
| 5. Most physicians are not compensated based on incentives for quality care. |  |  |  |  |  |
| 6. Nurse-patient communication is closely linked to satisfaction ratings by patients. |  |  |  |  |  |
| 7. Most hospitals are compensated based on incentives for quality care. |  |  |  |  |  |
| 8. Verbal and nonverbal communication may be portrayed by patients negatively. |  |  |  |  |  |
| 9. ‘DMAIC’ is a strategy for process improvement. |  |  |  |  |  |
| 10. Strategies such as ‘AIDET’ can be used to improve communication abilities. |  |  |  |  |  |
| 11. Patients can be highly satisfied, despite having poor health outcomes. |  |  |  |  |  |
| 12. HCAHPS is a standardized survey used to obtain patients’ perspectives on healthcare. |  |  |  |  |  |
| 13. The utility of HCAHPS surveying is that patients can compare different hospitals’ performance. |  |  |  |  |  |
| 14. MIPS is a quality based system used to determine payments to clinicians for Medicare enrollees. |  |  |  |  |  |
| 15. ‘Managing up’ other aspects of hospital care can be helpful with improving the patient experience. |  |  |  |  |  |
| 16. Healthcare organizations are evolving and conceptualizing care to promote patient experience. |  |  |  |  |  |
| 17. A positive patient experience may improve adherence with aspects of care. |  |  |  |  |  |
| 18. Patient satisfaction is relevant to my role as a health care provider. |  |  |  |  |  |
| 19. The ‘reputation management’ industry addresses concerns with online ratings of physicians by patients. |  |  |  |  |  |
| 20. Inadequate physician-patient communication has been linked to hospital readmissions. |  |  |  |  |  |

The Patient Experience Curriculum Pre- and Post-Survey
