# Supplementary material for: Understanding Patient Experience: A Course for Residents
Source: MedEdPORTAL. 2017 Mar 22;13:10558. doi: 10.15766/mep_2374-8265.10558 (PMC6342057; doi:10.15766/mep_2374-8265.10558)
Supplement: Supplementary file 1 — A. Pre- and Postsession Survey.docx B. Understanding the Patient Experience Presentation.pptx C. Self-Assessment of Patient Encounters.docx D. Facilitator Questions.docx E. Patient Survey Questions.docx [file mep-13-10558-s001.zip › B. Understanding the Patient Experience Presentation.pptx]

## Slide 1
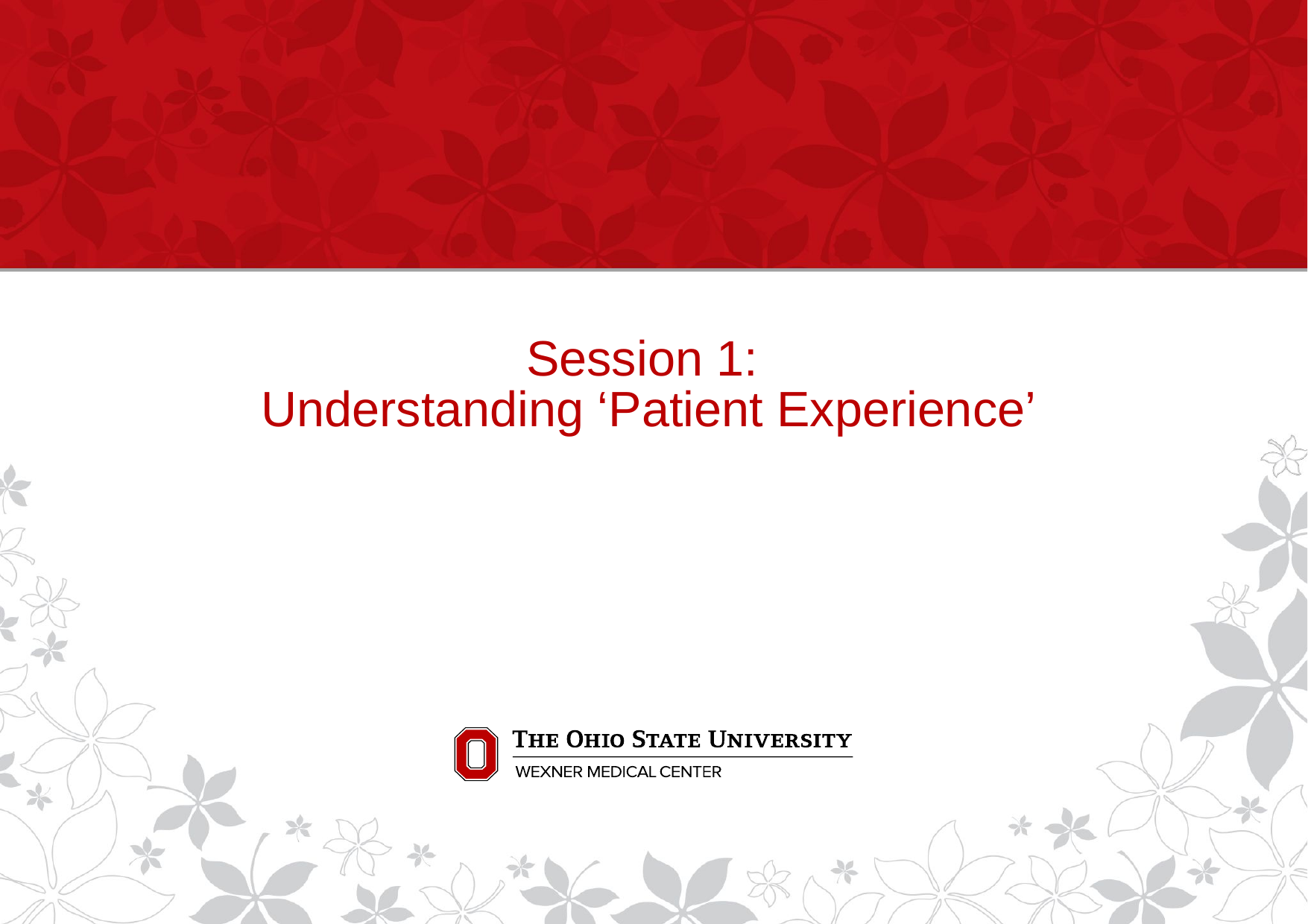

# Session 1: Understanding ‘Patient Experience’

## Slide 2
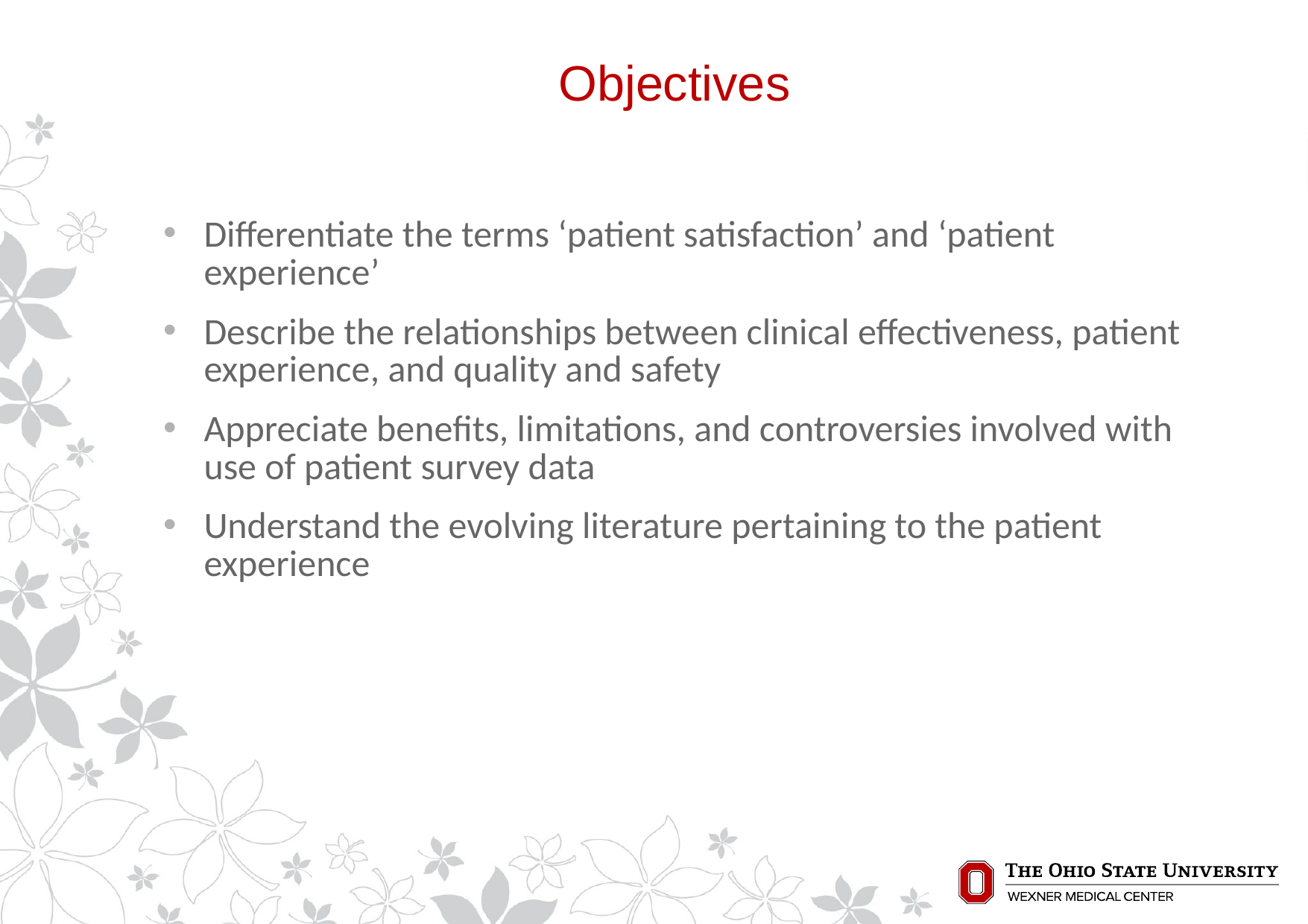

# Objectives
Differentiate the terms ‘patient satisfaction’ and ‘patient experience’
Describe the relationships between clinical effectiveness, patient experience, and quality and safety
Appreciate benefits, limitations, and controversies involved with use of patient survey data
Understand the evolving literature pertaining to the patient experience

## Slide 3
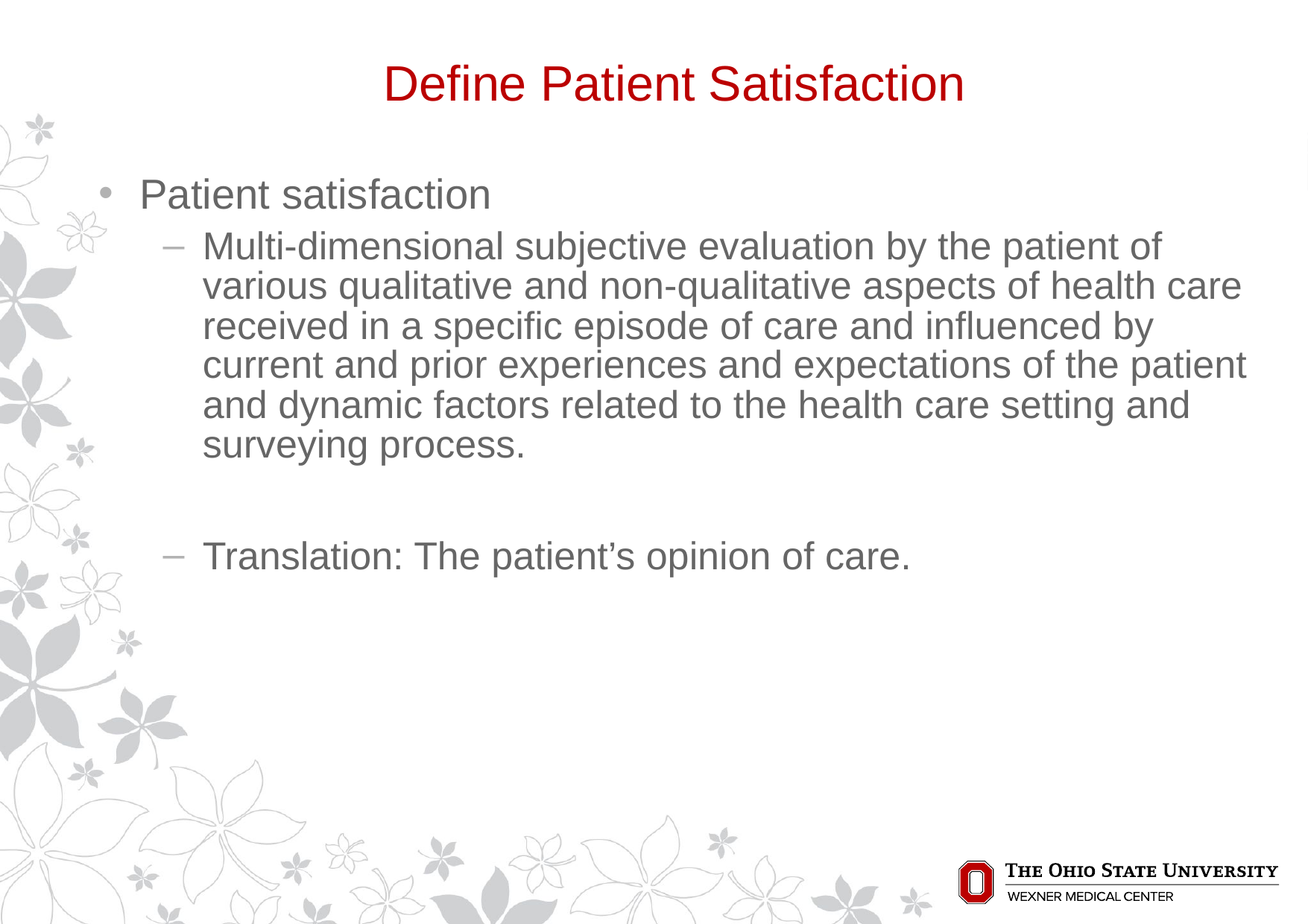

# Define Patient Satisfaction
Patient satisfaction
Multi-dimensional subjective evaluation by the patient of various qualitative and non-qualitative aspects of health care received in a specific episode of care and influenced by current and prior experiences and expectations of the patient and dynamic factors related to the health care setting and surveying process.
Translation: The patient’s opinion of care.

## Slide 4
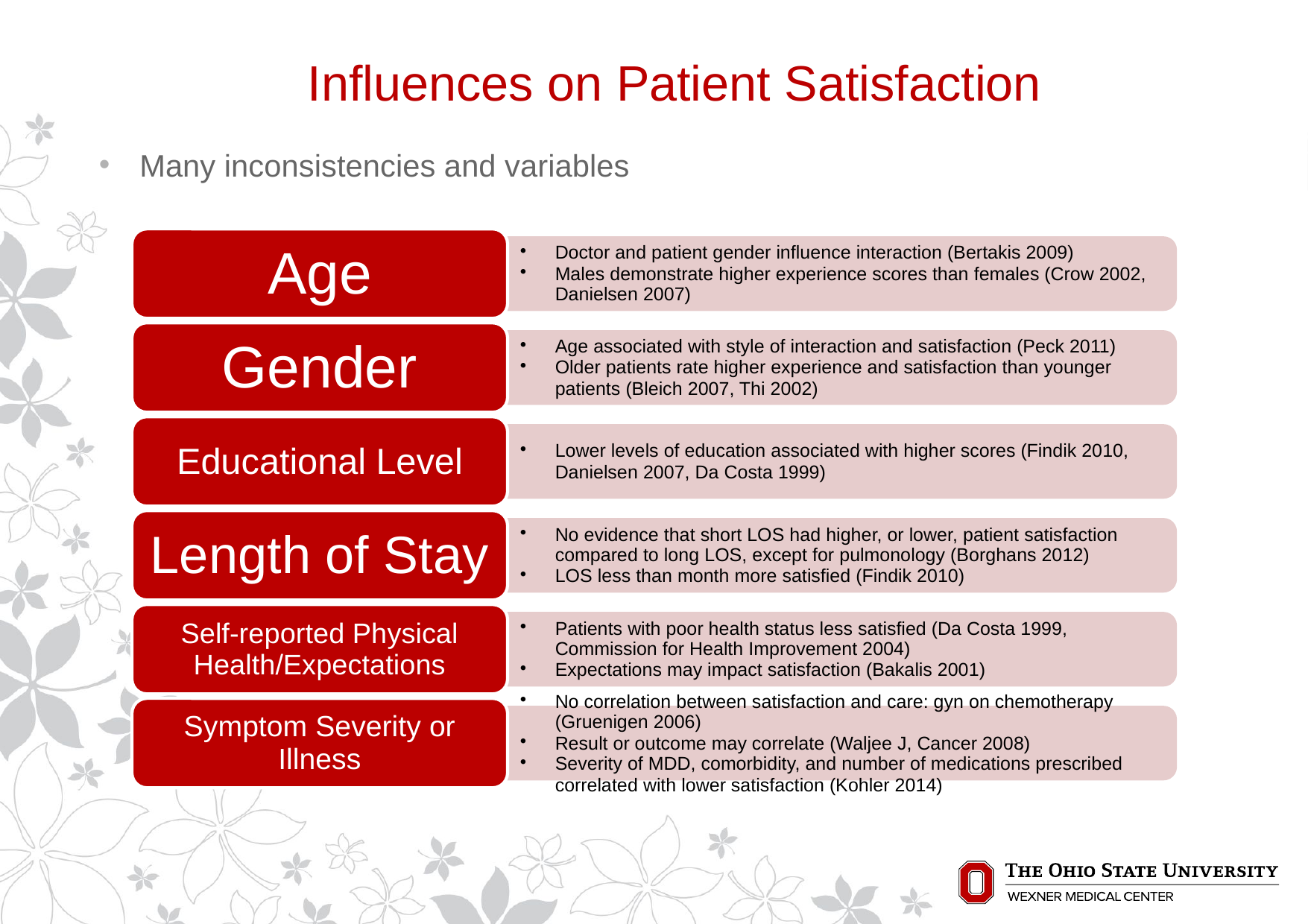

# Influences on Patient Satisfaction
Many inconsistencies and variables

## Slide 5
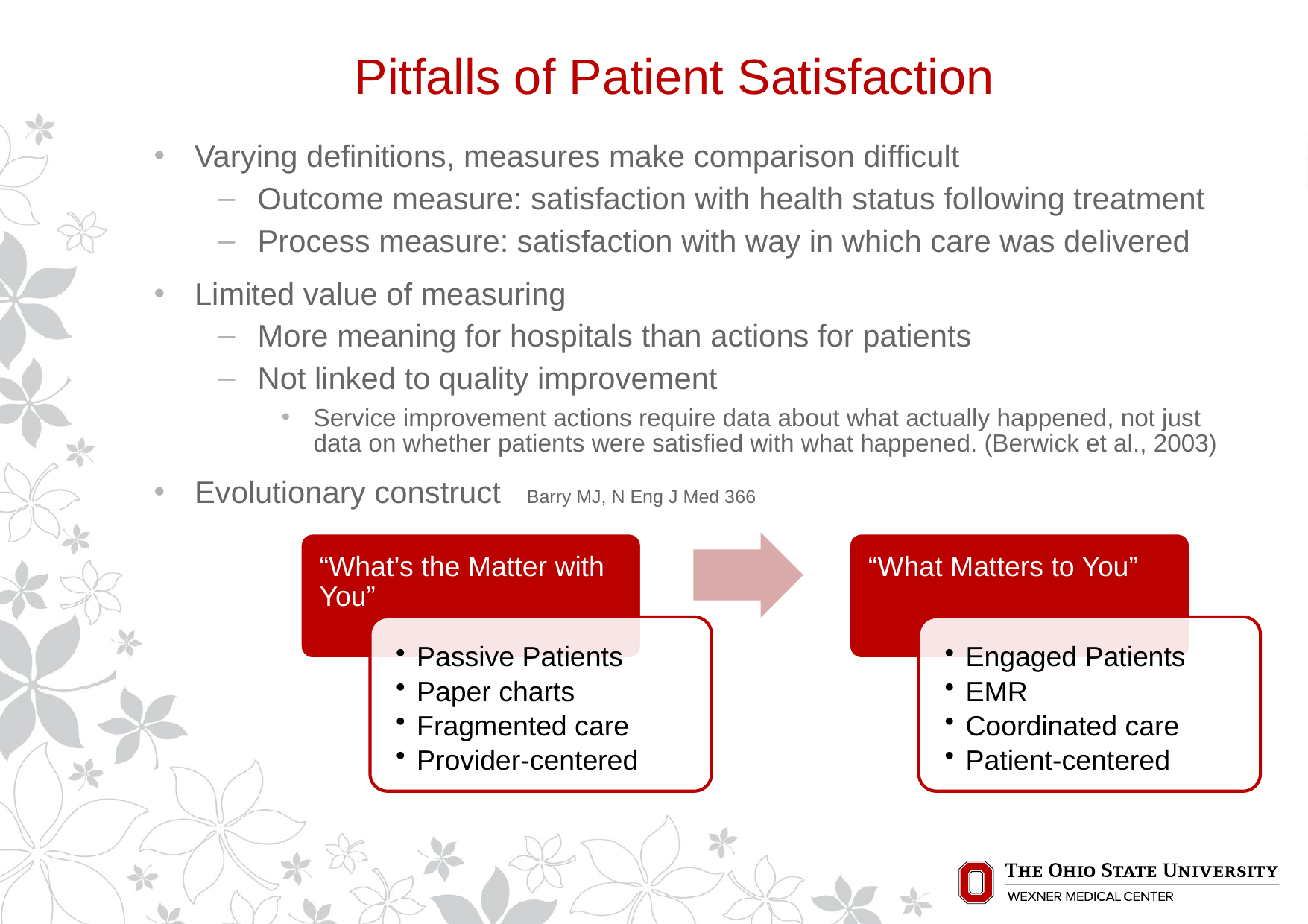

# Pitfalls of Patient Satisfaction
Varying definitions, measures make comparison difficult
Outcome measure: satisfaction with health status following treatment
Process measure: satisfaction with way in which care was delivered
Limited value of measuring
More meaning for hospitals than actions for patients
Not linked to quality improvement
Service improvement actions require data about what actually happened, not just data on whether patients were satisfied with what happened. (Berwick et al., 2003)
Evolutionary construct Barry MJ, N Eng J Med 366

## Slide 6
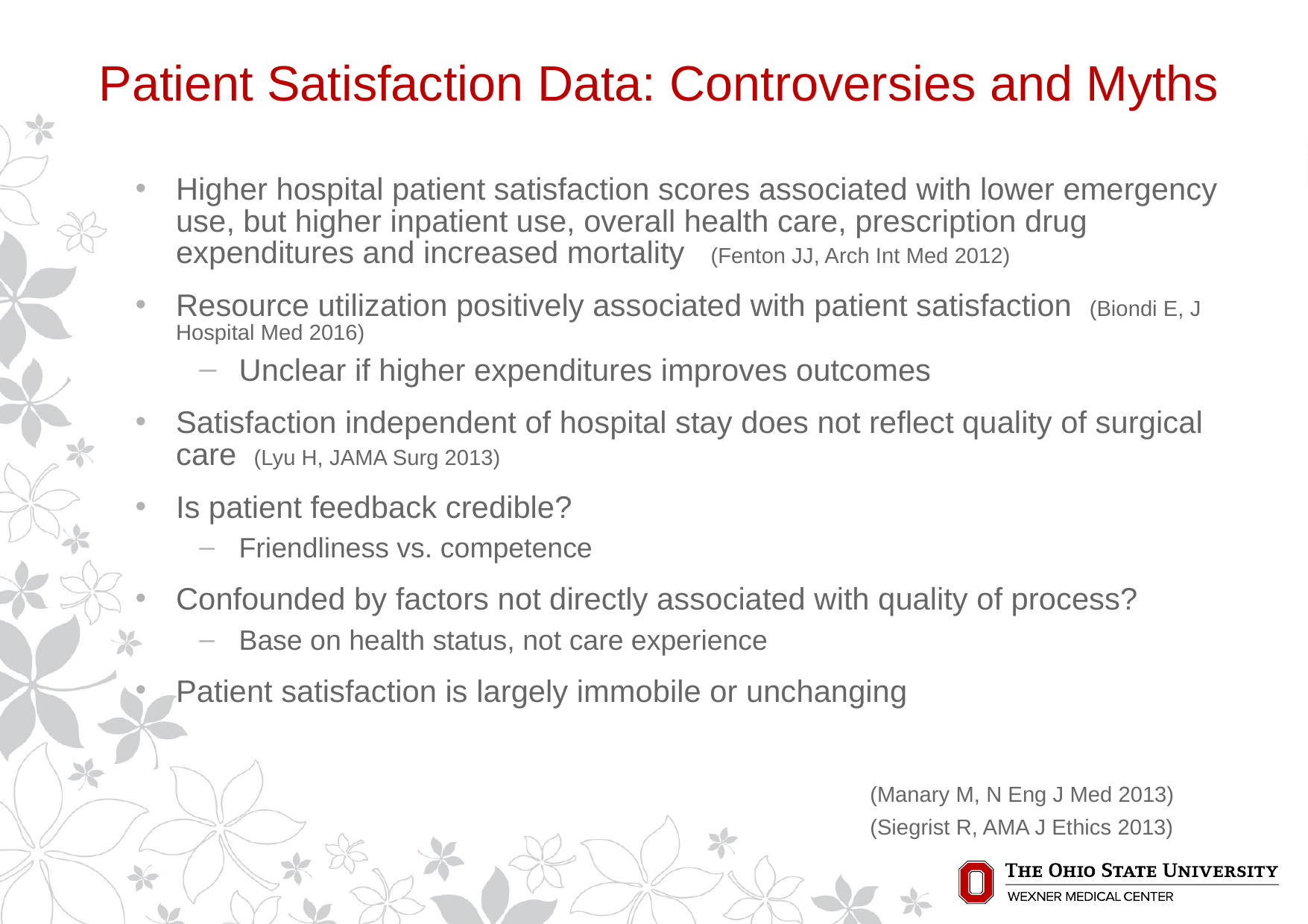

# Patient Satisfaction Data: Controversies and Myths
Higher hospital patient satisfaction scores associated with lower emergency use, but higher inpatient use, overall health care, prescription drug expenditures and increased mortality (Fenton JJ, Arch Int Med 2012)
Resource utilization positively associated with patient satisfaction (Biondi E, J Hospital Med 2016)
Unclear if higher expenditures improves outcomes
Satisfaction independent of hospital stay does not reflect quality of surgical care (Lyu H, JAMA Surg 2013)
Is patient feedback credible?
Friendliness vs. competence
Confounded by factors not directly associated with quality of process?
Base on health status, not care experience
Patient satisfaction is largely immobile or unchanging
						(Manary M, N Eng J Med 2013)
						(Siegrist R, AMA J Ethics 2013)

## Slide 7
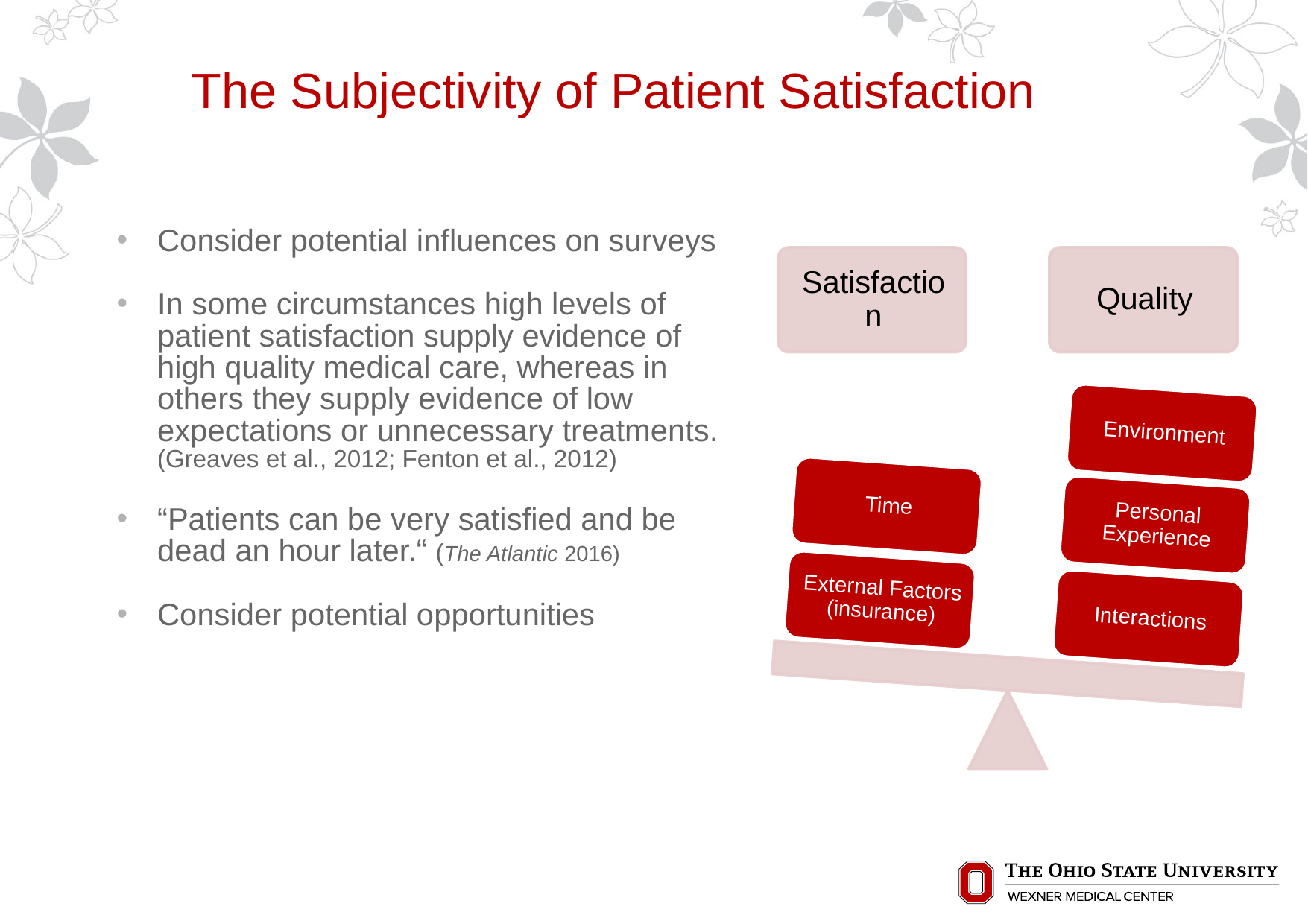

# The Subjectivity of Patient Satisfaction
Consider potential influences on surveys
In some circumstances high levels of patient satisfaction supply evidence of high quality medical care, whereas in others they supply evidence of low expectations or unnecessary treatments. (Greaves et al., 2012; Fenton et al., 2012)
“Patients can be very satisfied and be dead an hour later.“ (The Atlantic 2016)
Consider potential opportunities

## Slide 8
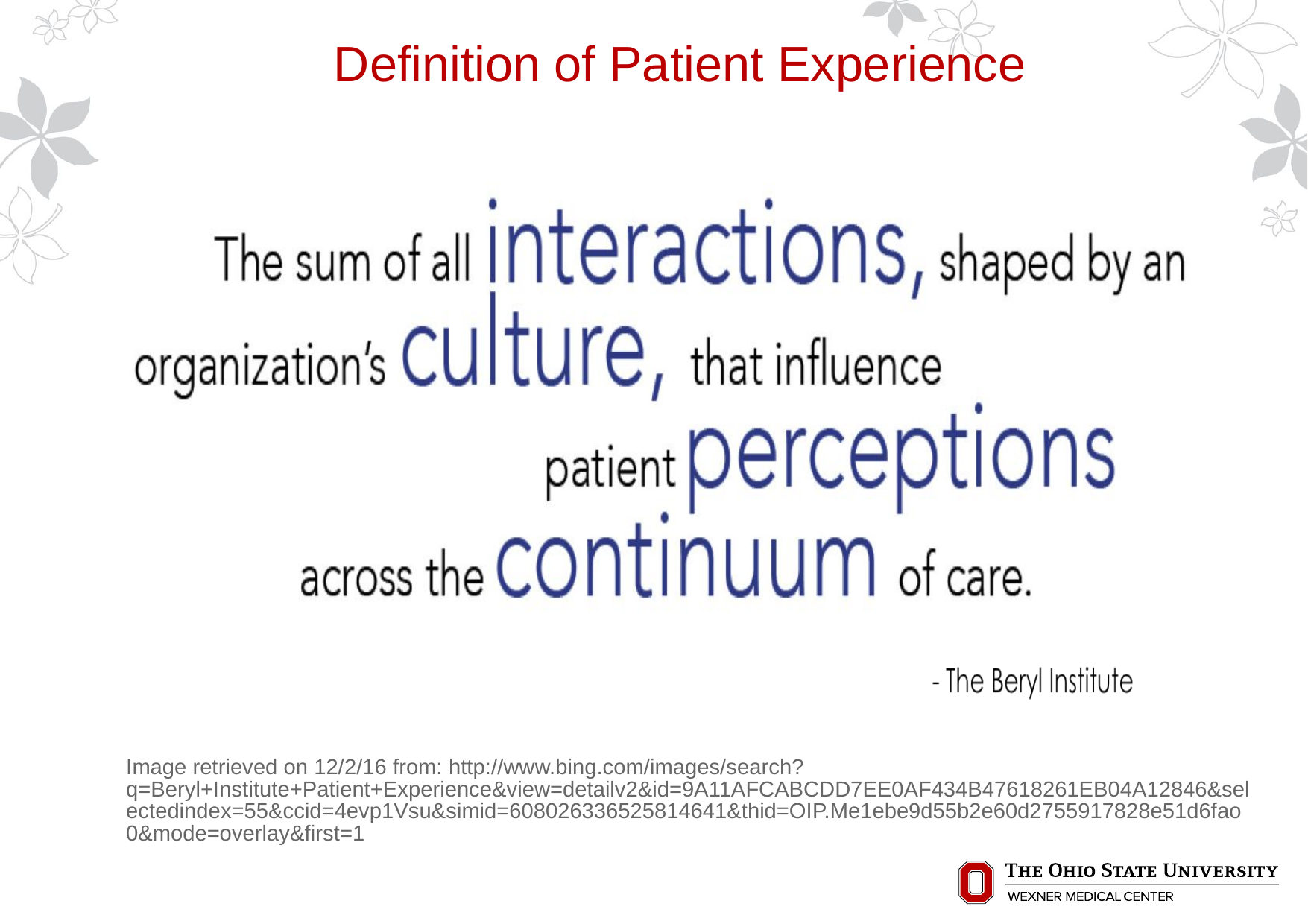

# Definition of Patient Experience
Image retrieved on 12/2/16 from: http://www.bing.com/images/search?q=Beryl+Institute+Patient+Experience&view=detailv2&id=9A11AFCABCDD7EE0AF434B47618261EB04A12846&selectedindex=55&ccid=4evp1Vsu&simid=608026336525814641&thid=OIP.Me1ebe9d55b2e60d2755917828e51d6fao0&mode=overlay&first=1

## Slide 9
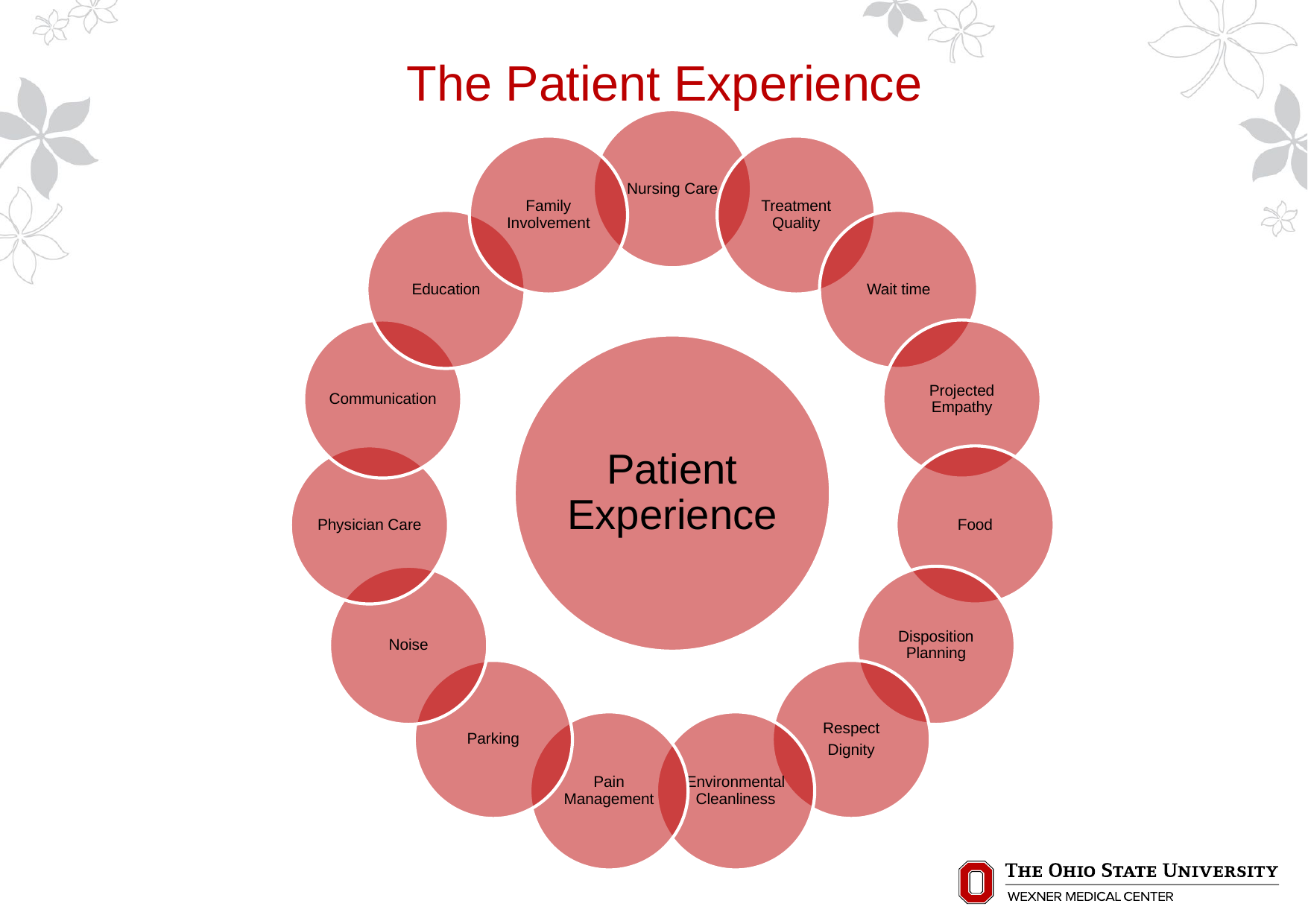

# The Patient Experience

## Slide 10
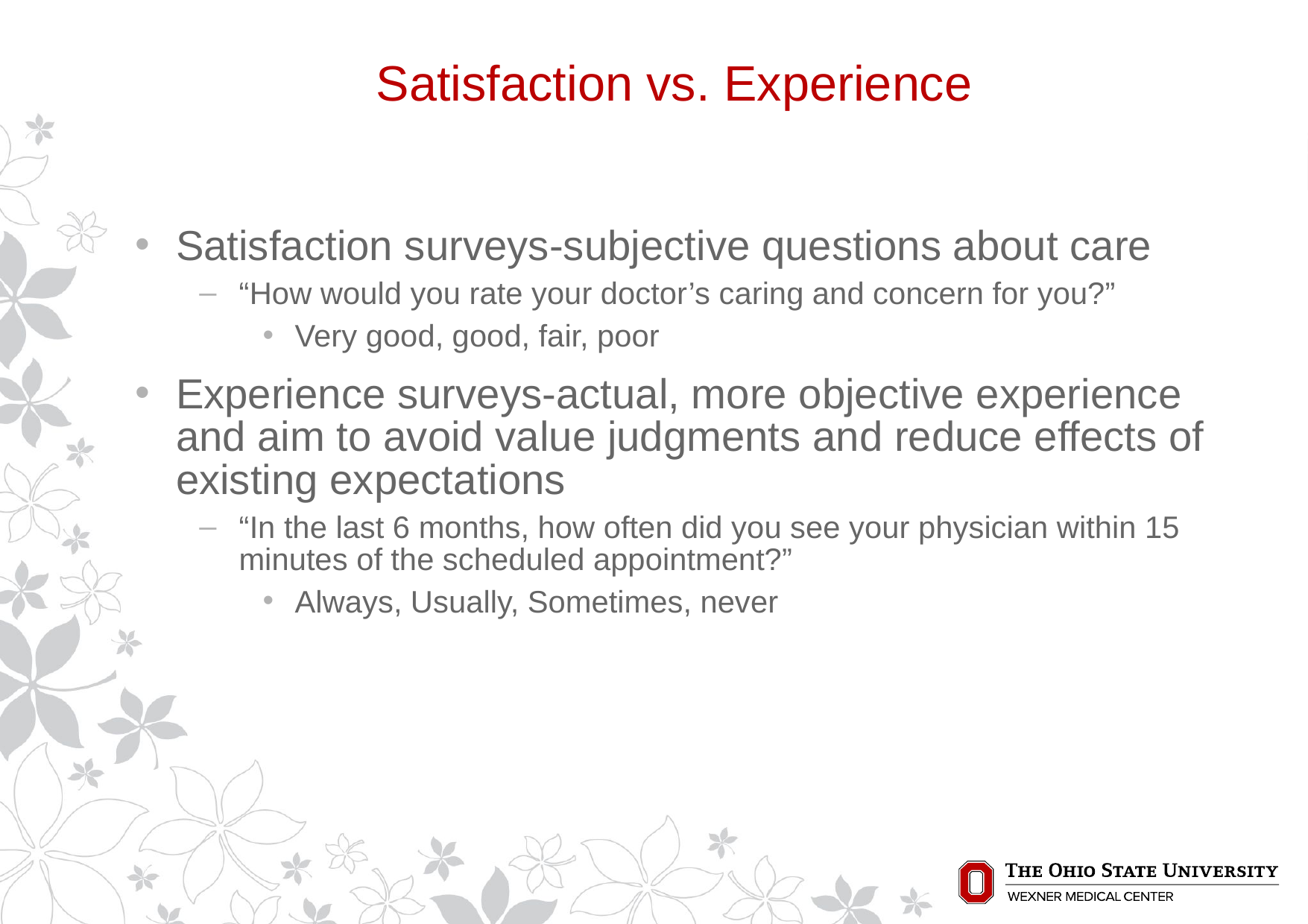

# Satisfaction vs. Experience
Satisfaction surveys-subjective questions about care
“How would you rate your doctor’s caring and concern for you?”
Very good, good, fair, poor
Experience surveys-actual, more objective experience and aim to avoid value judgments and reduce effects of existing expectations
“In the last 6 months, how often did you see your physician within 15 minutes of the scheduled appointment?”
Always, Usually, Sometimes, never

## Slide 11
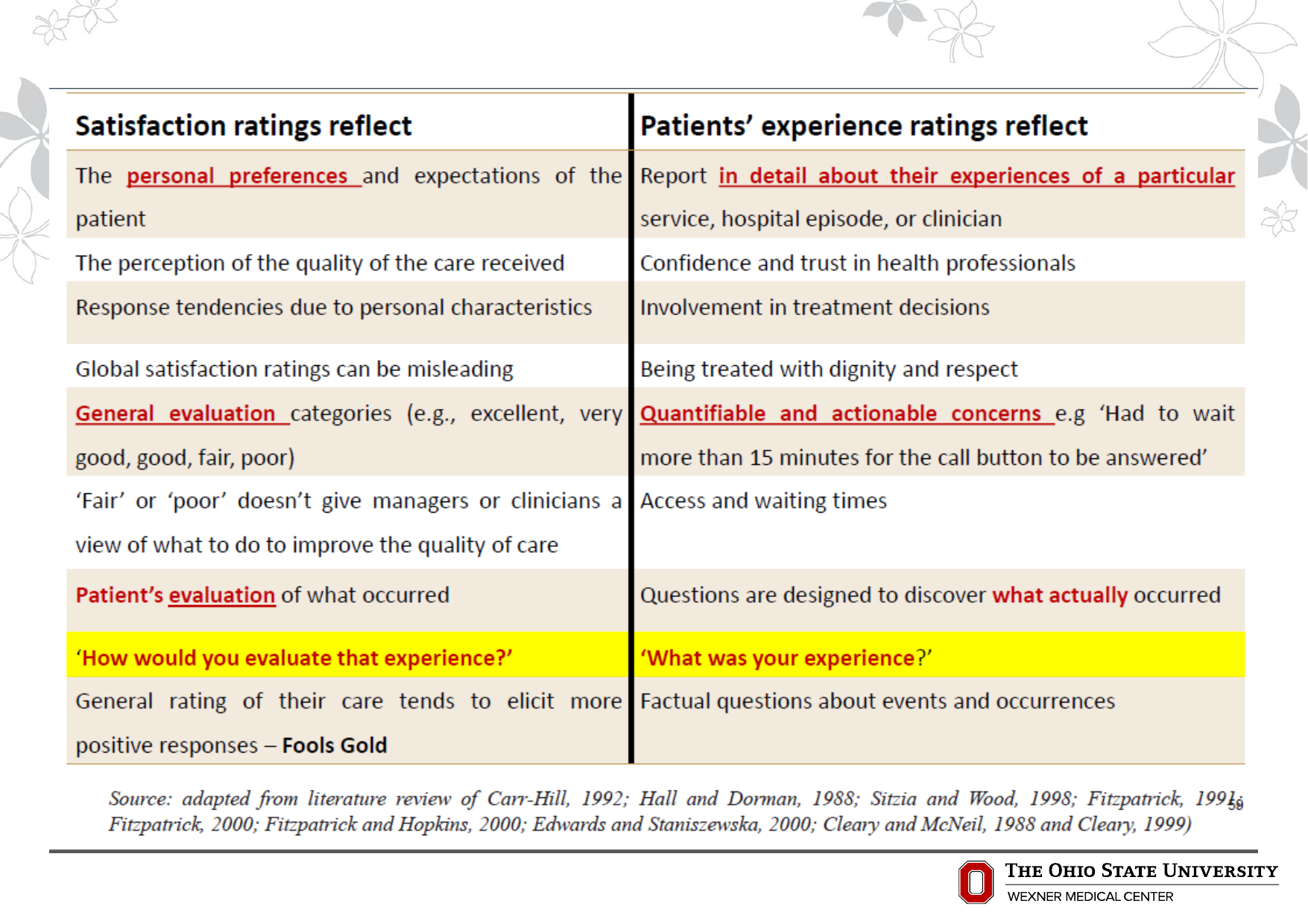

## Slide 12
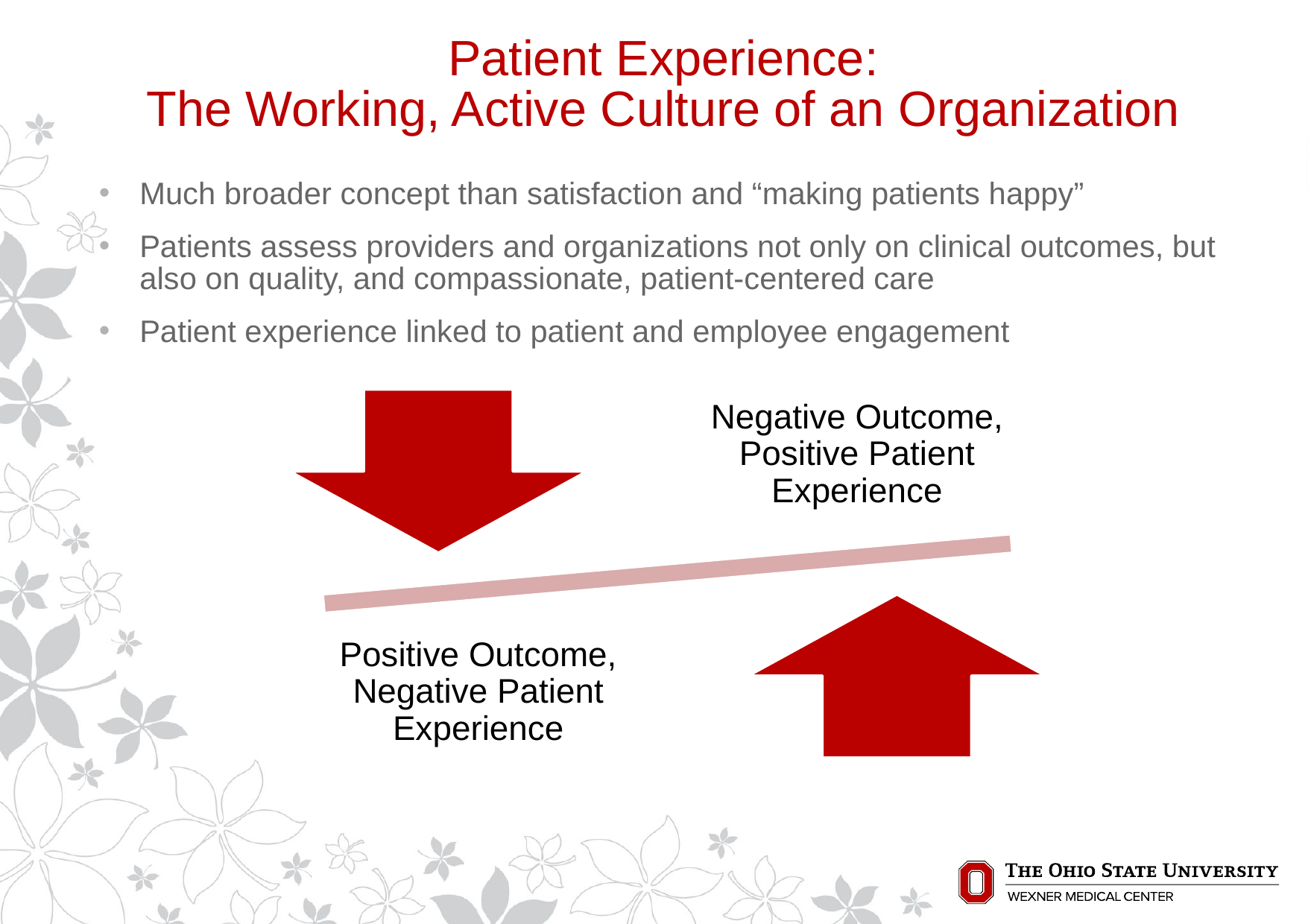

# Patient Experience: The Working, Active Culture of an Organization
Much broader concept than satisfaction and “making patients happy”
Patients assess providers and organizations not only on clinical outcomes, but also on quality, and compassionate, patient-centered care
Patient experience linked to patient and employee engagement

## Slide 13
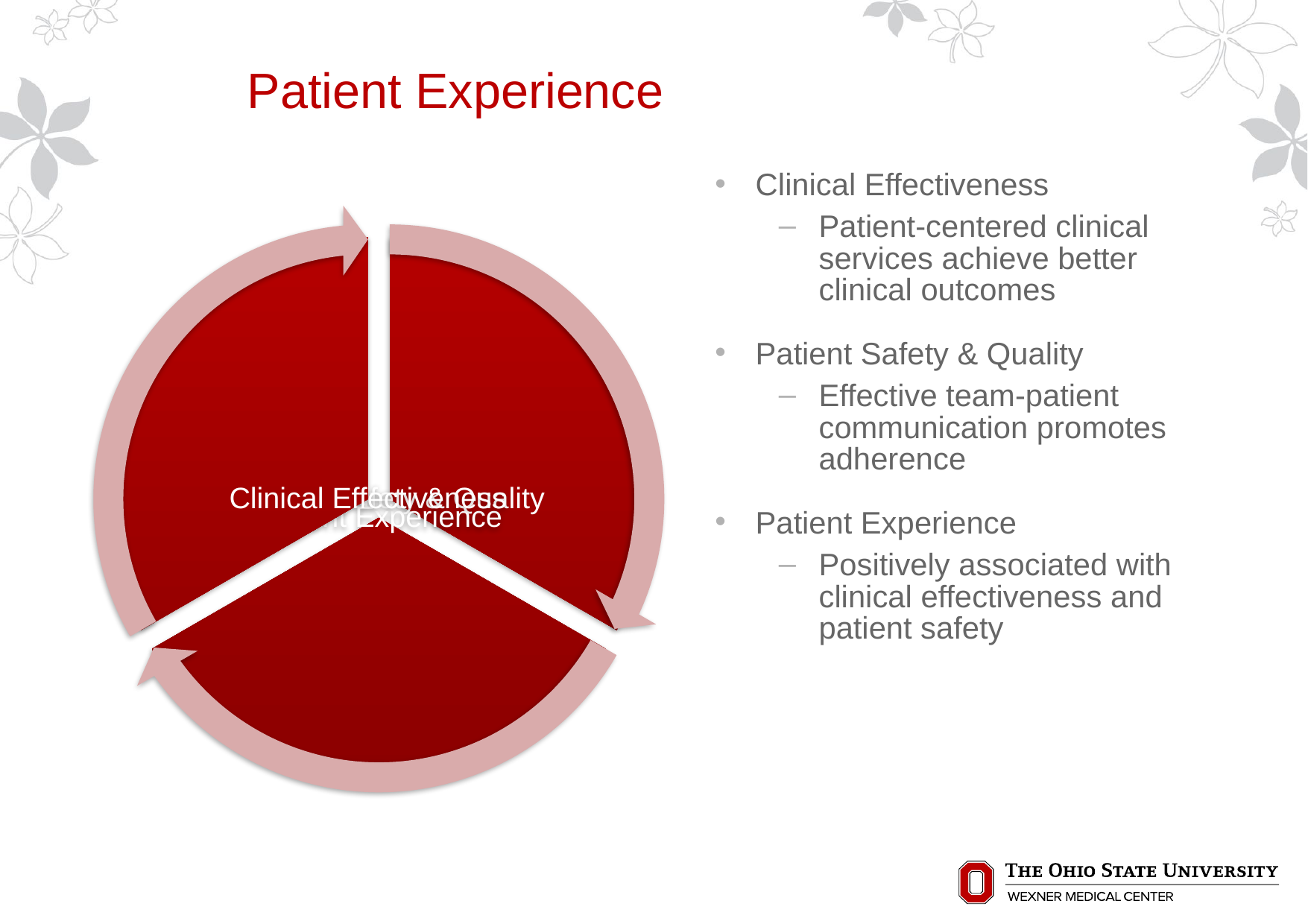

# Patient Experience
Clinical Effectiveness
Patient-centered clinical services achieve better clinical outcomes
Patient Safety & Quality
Effective team-patient communication promotes adherence
Patient Experience
Positively associated with clinical effectiveness and patient safety

## Slide 14
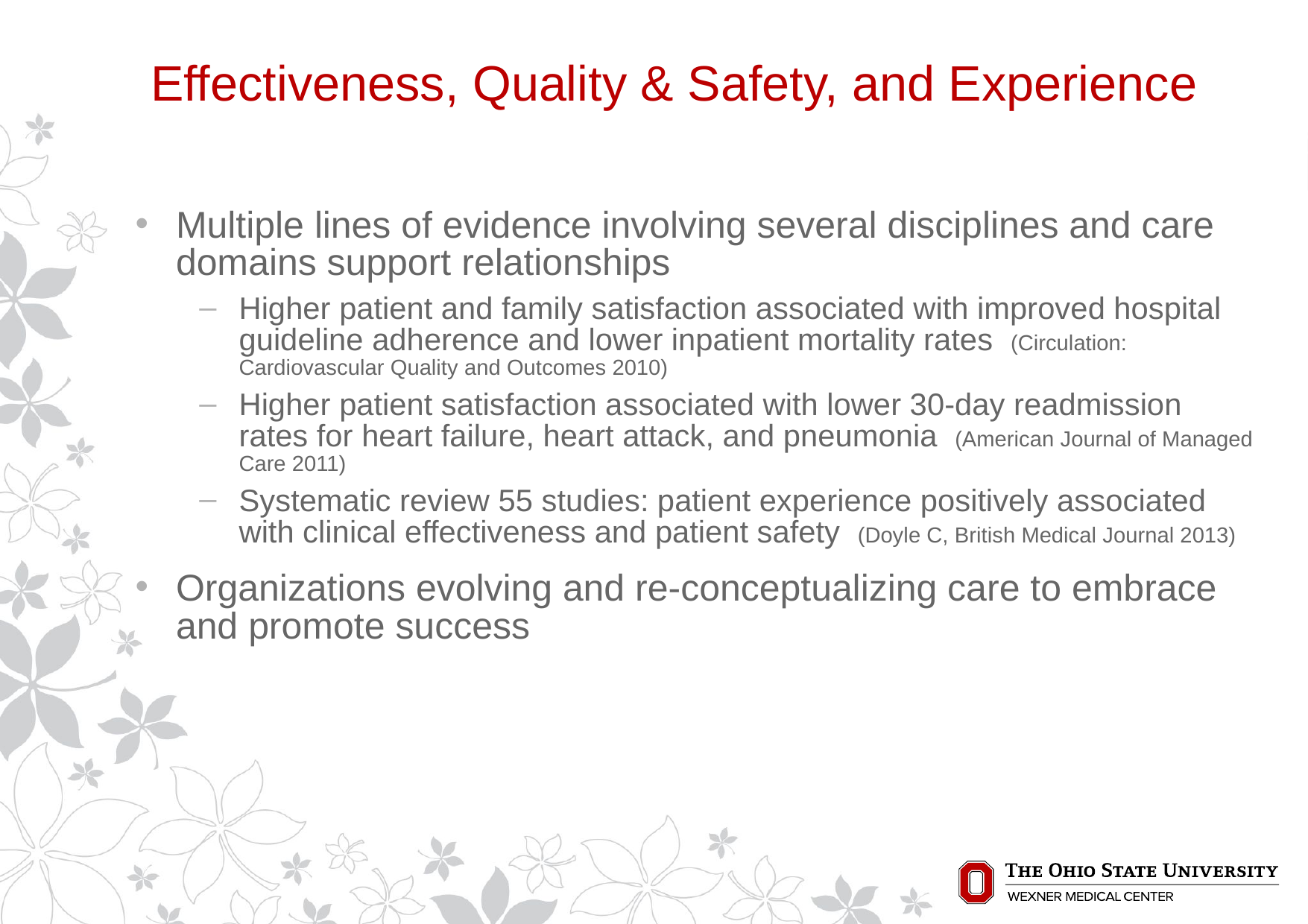

# Effectiveness, Quality & Safety, and Experience
Multiple lines of evidence involving several disciplines and care domains support relationships
Higher patient and family satisfaction associated with improved hospital guideline adherence and lower inpatient mortality rates (Circulation: Cardiovascular Quality and Outcomes 2010)
Higher patient satisfaction associated with lower 30-day readmission rates for heart failure, heart attack, and pneumonia (American Journal of Managed Care 2011)
Systematic review 55 studies: patient experience positively associated with clinical effectiveness and patient safety (Doyle C, British Medical Journal 2013)
Organizations evolving and re-conceptualizing care to embrace and promote success

## Slide 15
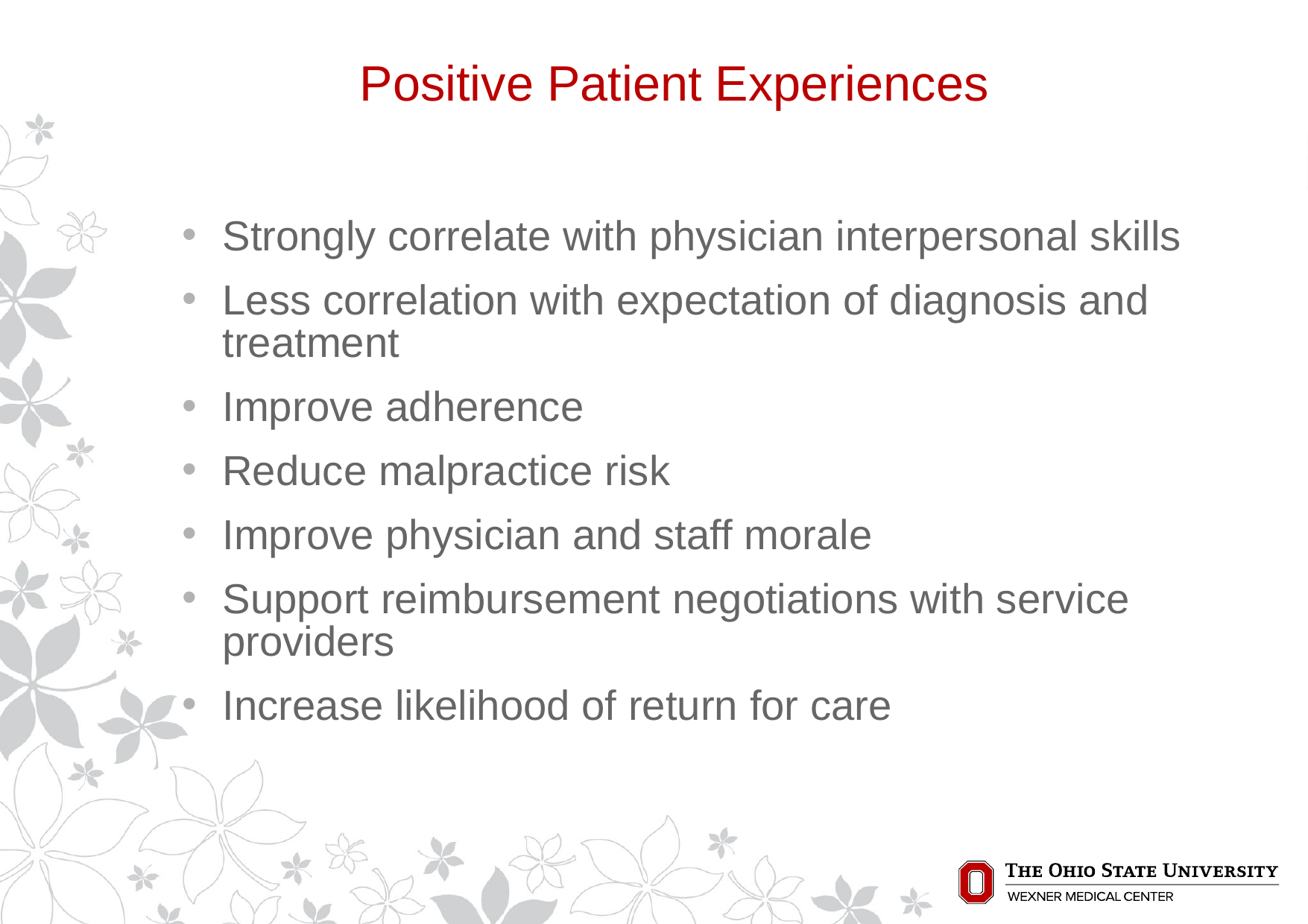

# Positive Patient Experiences
Strongly correlate with physician interpersonal skills
Less correlation with expectation of diagnosis and treatment
Improve adherence
Reduce malpractice risk
Improve physician and staff morale
Support reimbursement negotiations with service providers
Increase likelihood of return for care

## Slide 16
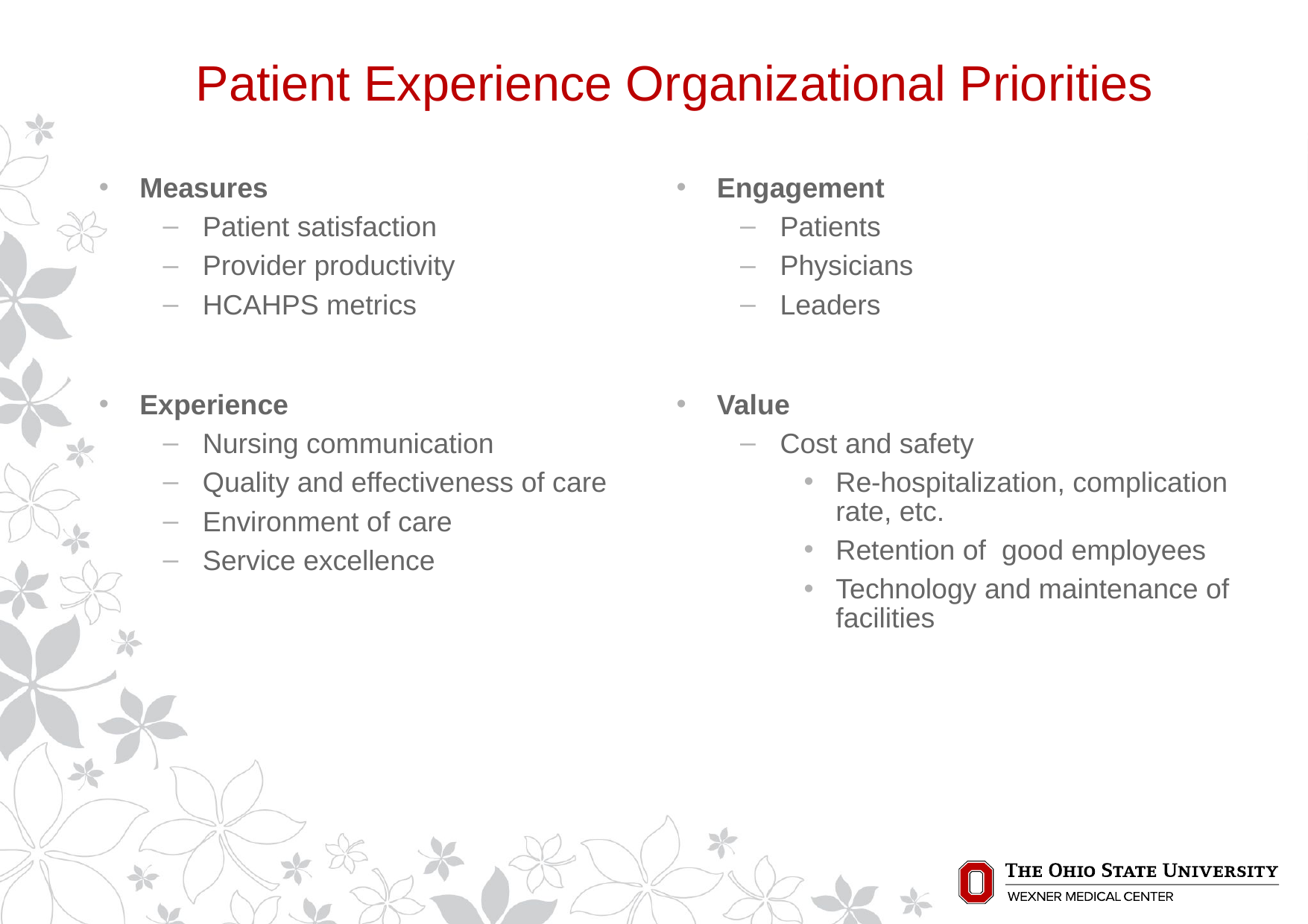

# Patient Experience Organizational Priorities
Measures
Patient satisfaction
Provider productivity
HCAHPS metrics
Experience
Nursing communication
Quality and effectiveness of care
Environment of care
Service excellence
Engagement
Patients
Physicians
Leaders
Value
Cost and safety
Re-hospitalization, complication rate, etc.
Retention of good employees
Technology and maintenance of facilities

## Slide 17
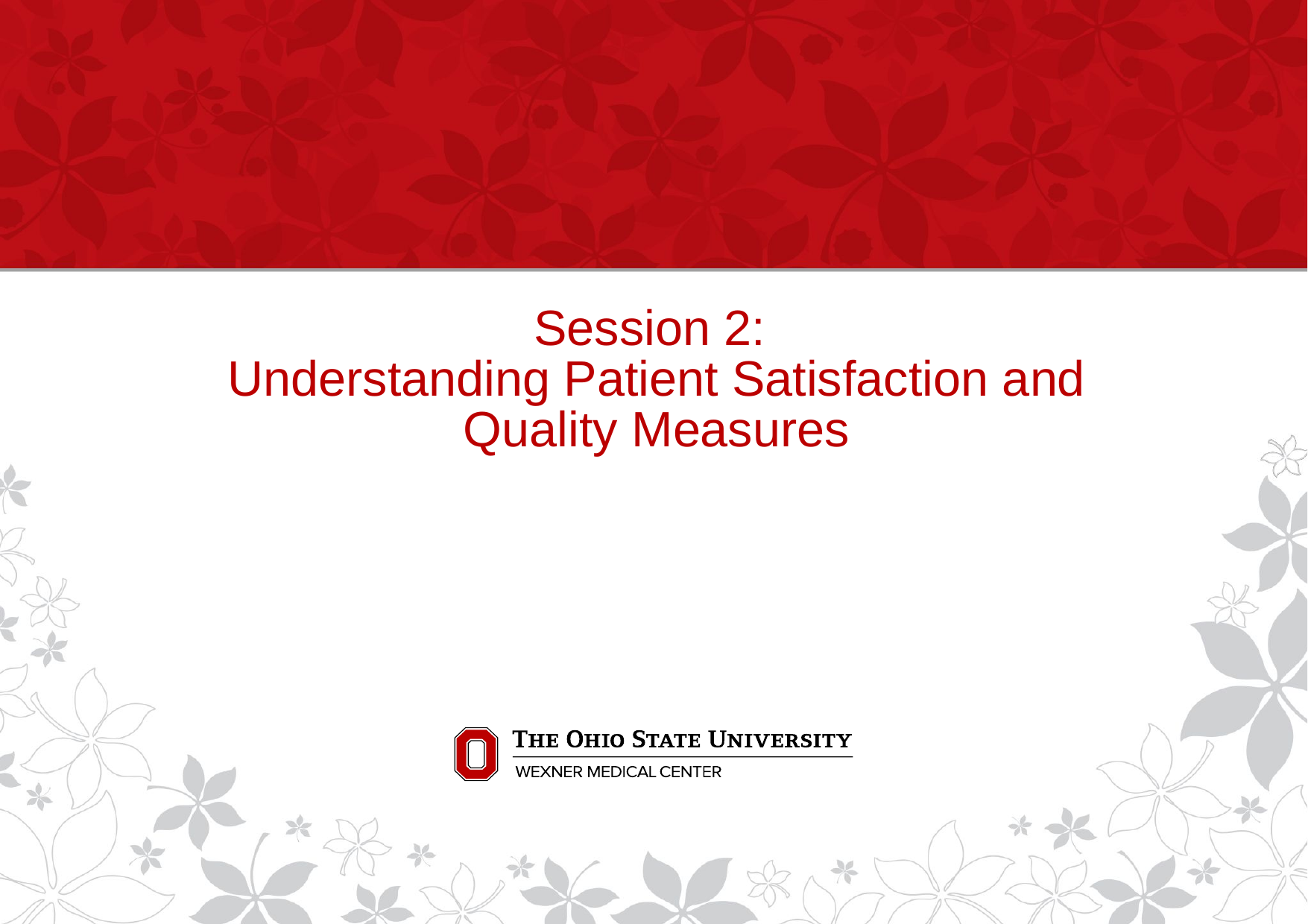

# Session 2: Understanding Patient Satisfaction and Quality Measures

## Slide 18
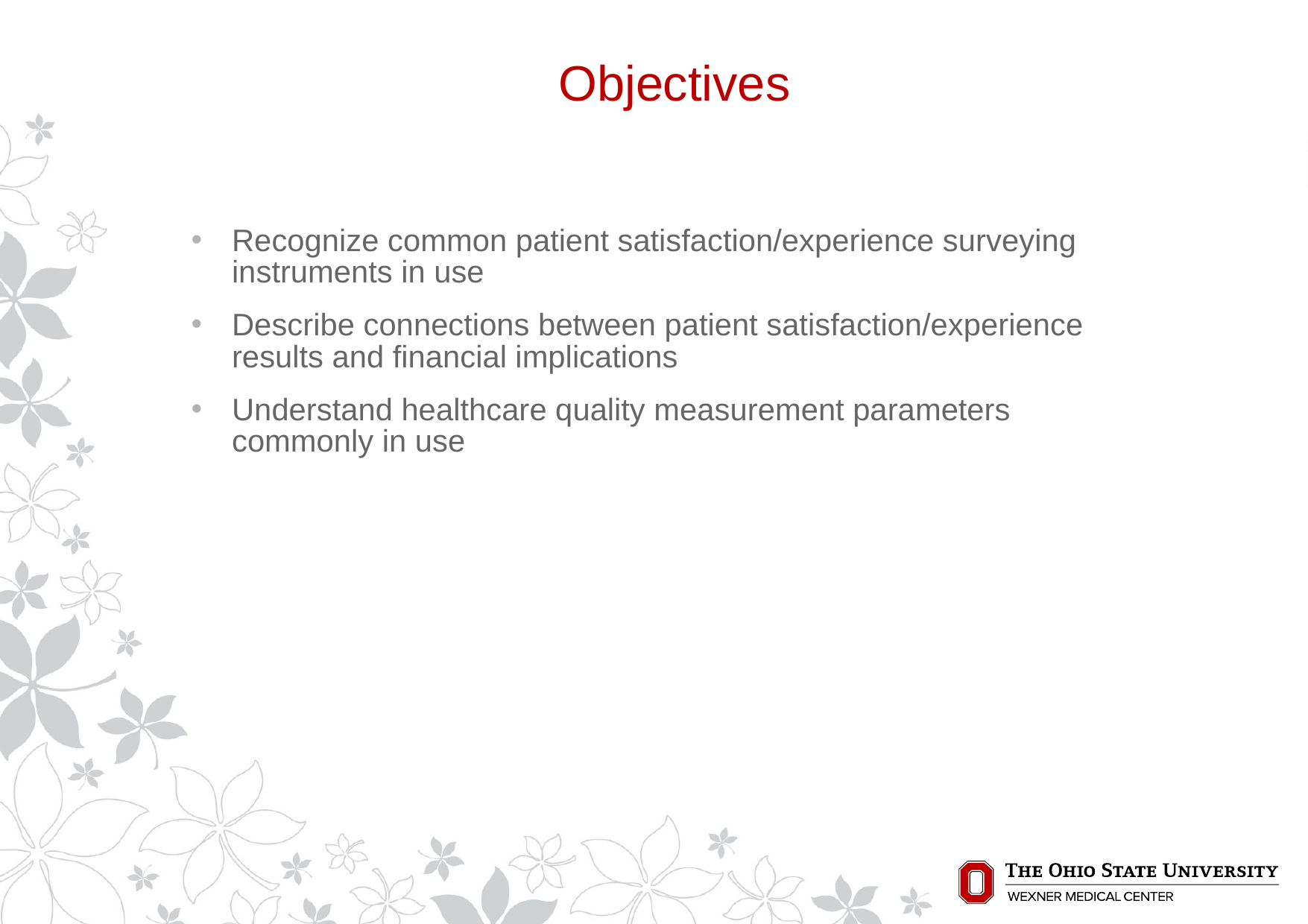

# Objectives
Recognize common patient satisfaction/experience surveying instruments in use
Describe connections between patient satisfaction/experience results and financial implications
Understand healthcare quality measurement parameters commonly in use

## Slide 19
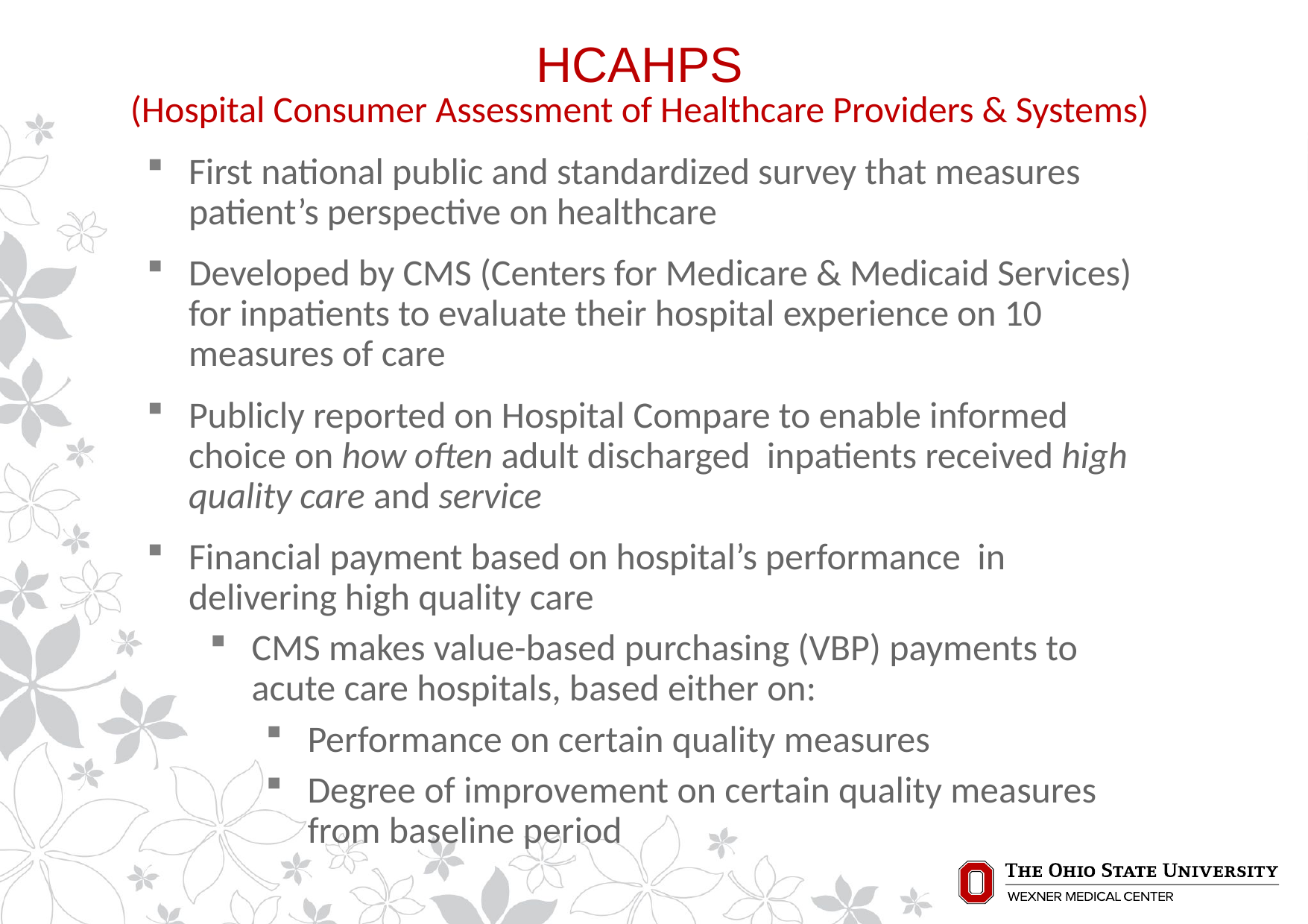

# HCAHPS (Hospital Consumer Assessment of Healthcare Providers & Systems)
First national public and standardized survey that measures patient’s perspective on healthcare
Developed by CMS (Centers for Medicare & Medicaid Services) for inpatients to evaluate their hospital experience on 10 measures of care
Publicly reported on Hospital Compare to enable informed choice on how often adult discharged inpatients received high quality care and service
Financial payment based on hospital’s performance in delivering high quality care
CMS makes value-based purchasing (VBP) payments to acute care hospitals, based either on:
Performance on certain quality measures
Degree of improvement on certain quality measures from baseline period

## Slide 20
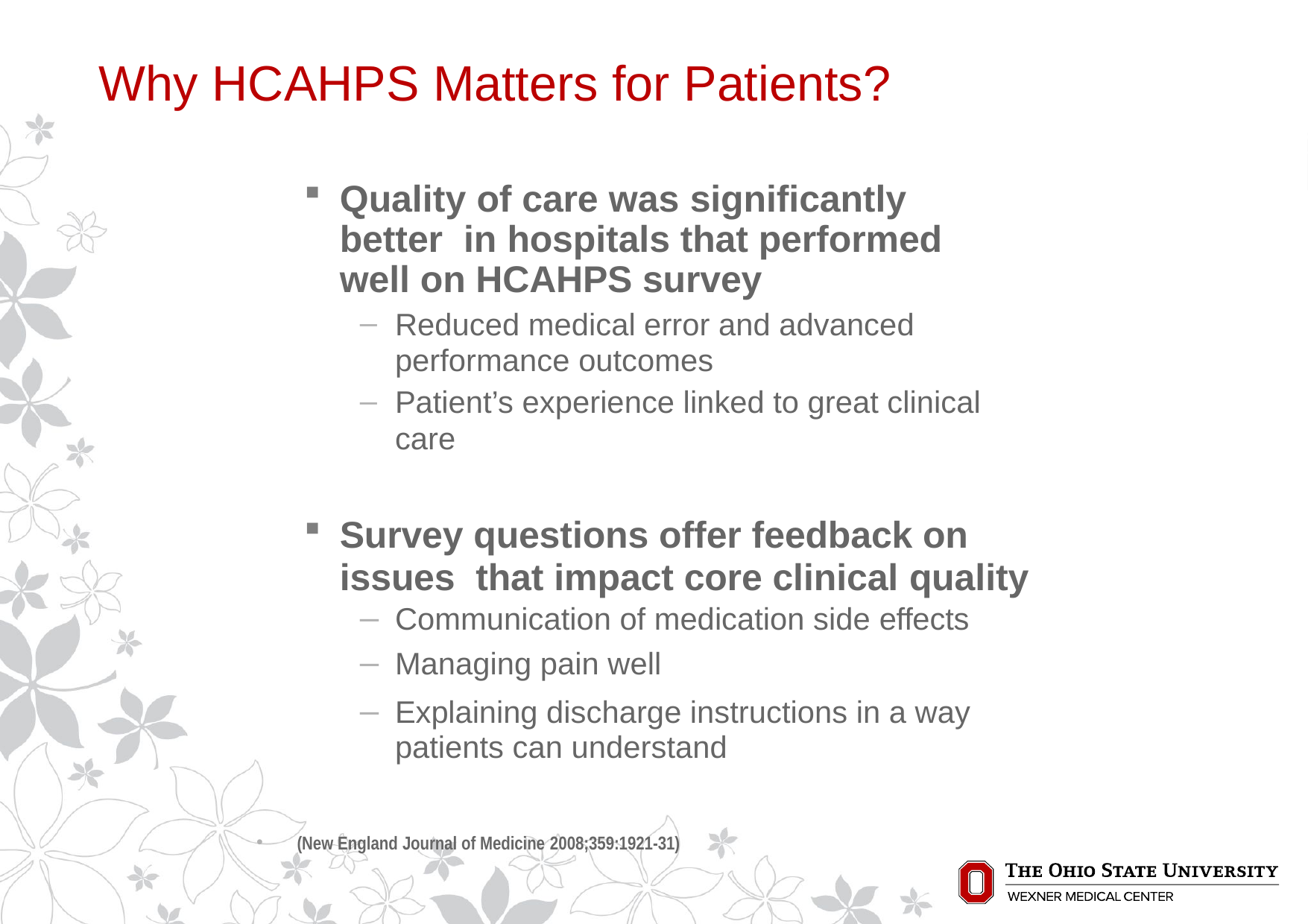

# Why HCAHPS Matters for Patients?
Quality of care was significantly better in hospitals that performed well on HCAHPS survey
Reduced medical error and advanced performance outcomes
Patient’s experience linked to great clinical care
Survey questions offer feedback on issues that impact core clinical quality
Communication of medication side effects
Managing pain well
Explaining discharge instructions in a way patients can understand
(New England Journal of Medicine 2008;359:1921-31)

## Slide 21
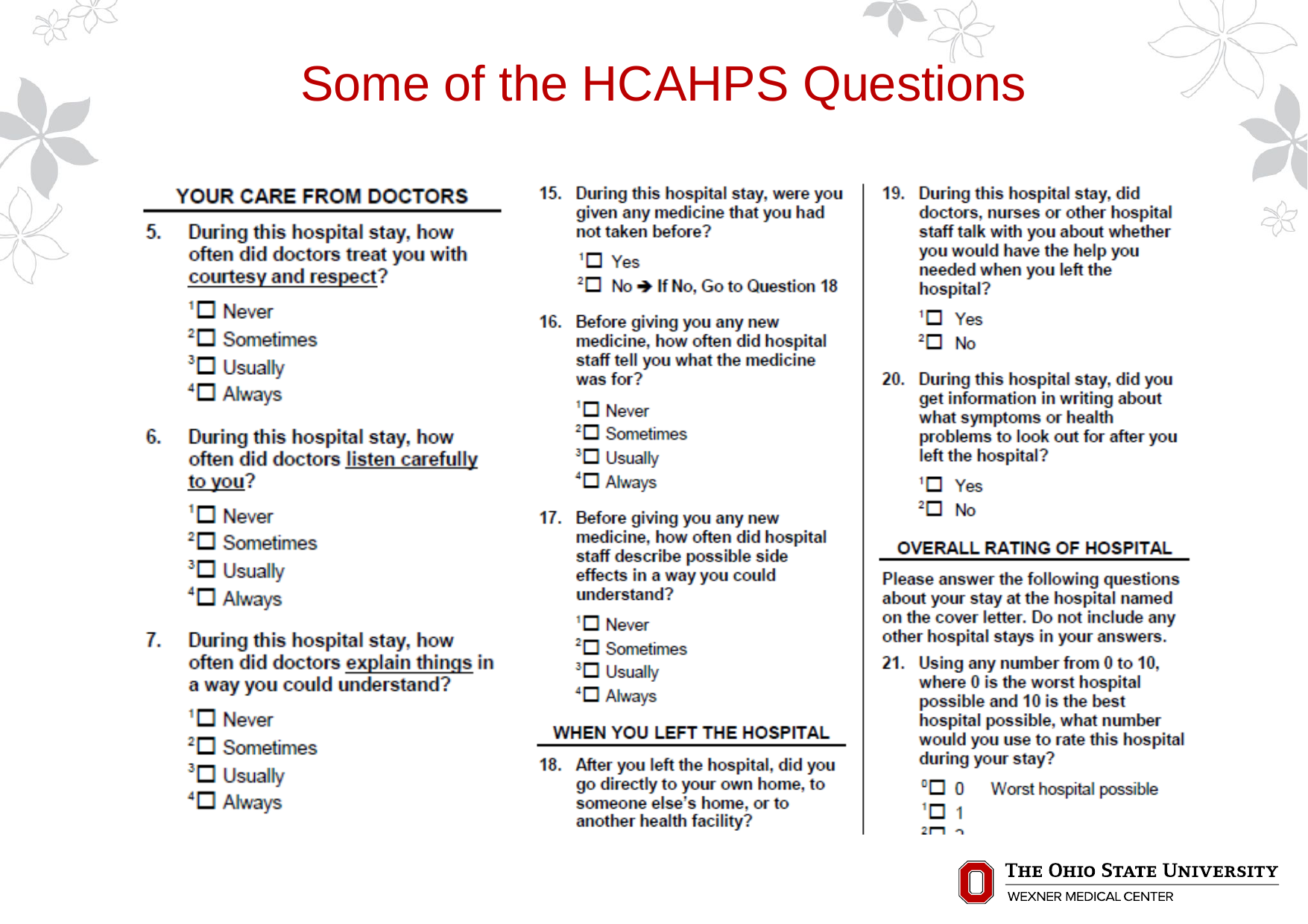

# Some of the HCAHPS Questions

## Slide 22
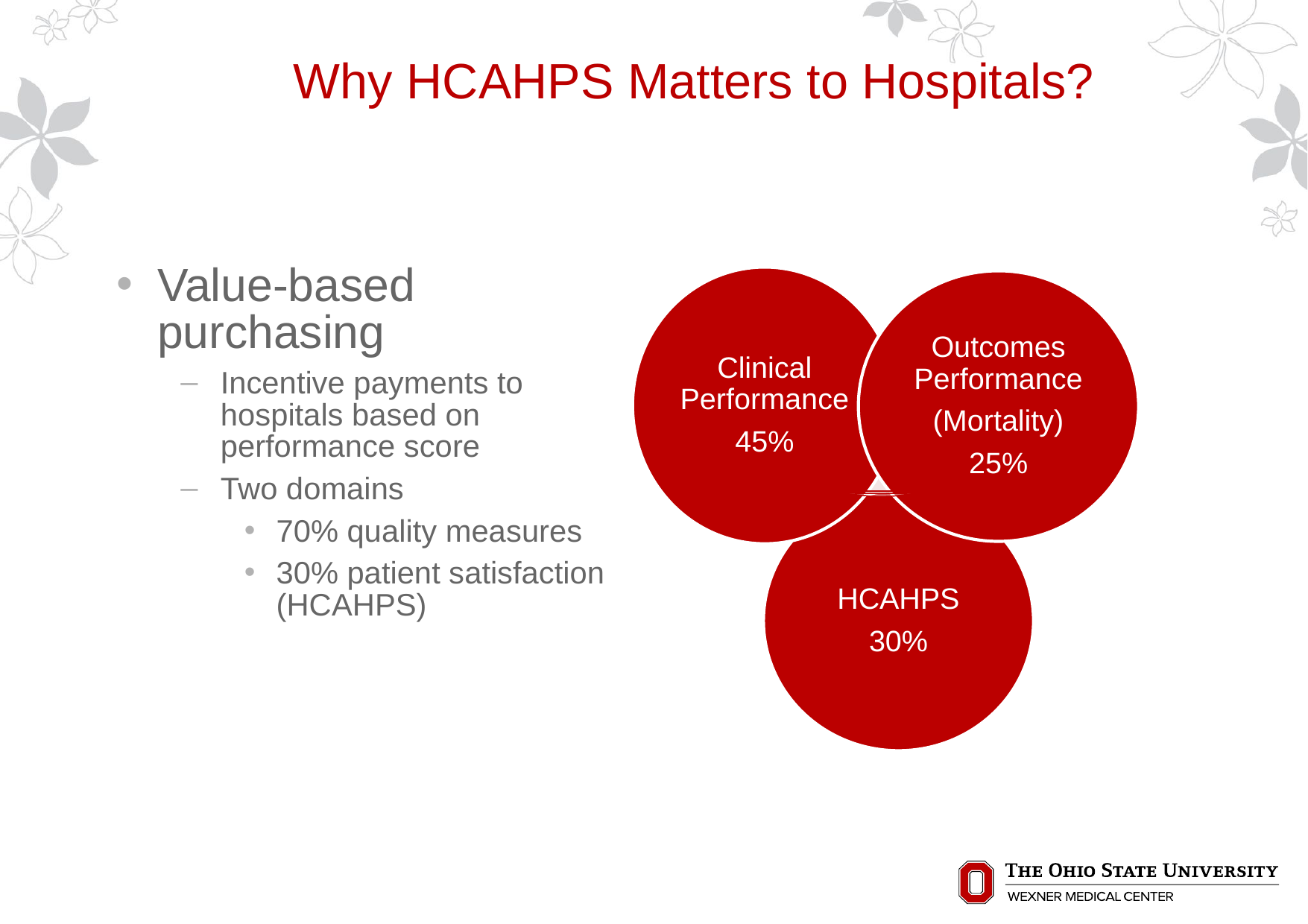

# Why HCAHPS Matters to Hospitals?
Value-based purchasing
Incentive payments to hospitals based on performance score
Two domains
70% quality measures
30% patient satisfaction (HCAHPS)

## Slide 23
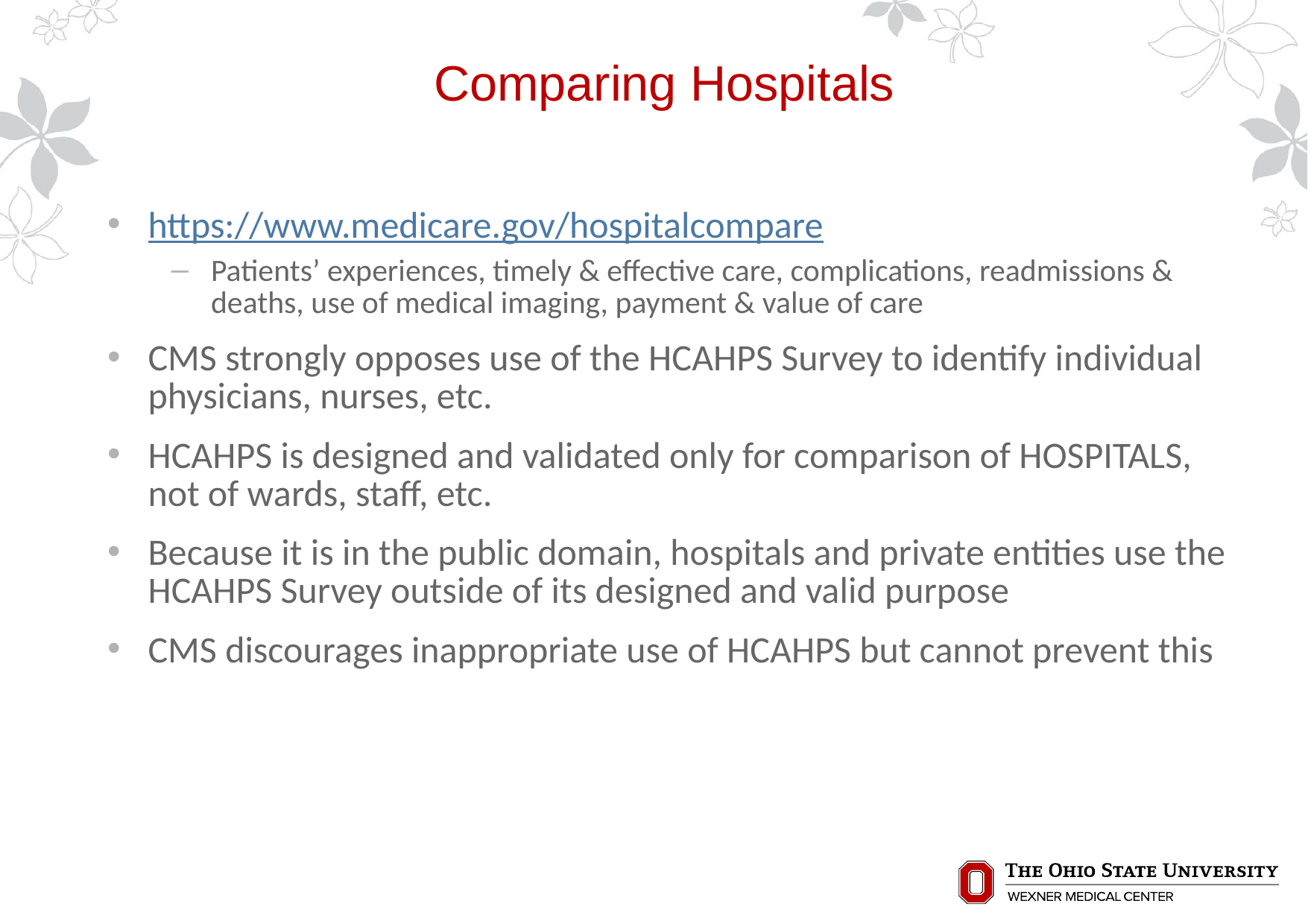

# Comparing Hospitals
https://www.medicare.gov/hospitalcompare
Patients’ experiences, timely & effective care, complications, readmissions & deaths, use of medical imaging, payment & value of care
CMS strongly opposes use of the HCAHPS Survey to identify individual physicians, nurses, etc.
HCAHPS is designed and validated only for comparison of HOSPITALS, not of wards, staff, etc.
Because it is in the public domain, hospitals and private entities use the HCAHPS Survey outside of its designed and valid purpose
CMS discourages inappropriate use of HCAHPS but cannot prevent this

## Slide 24
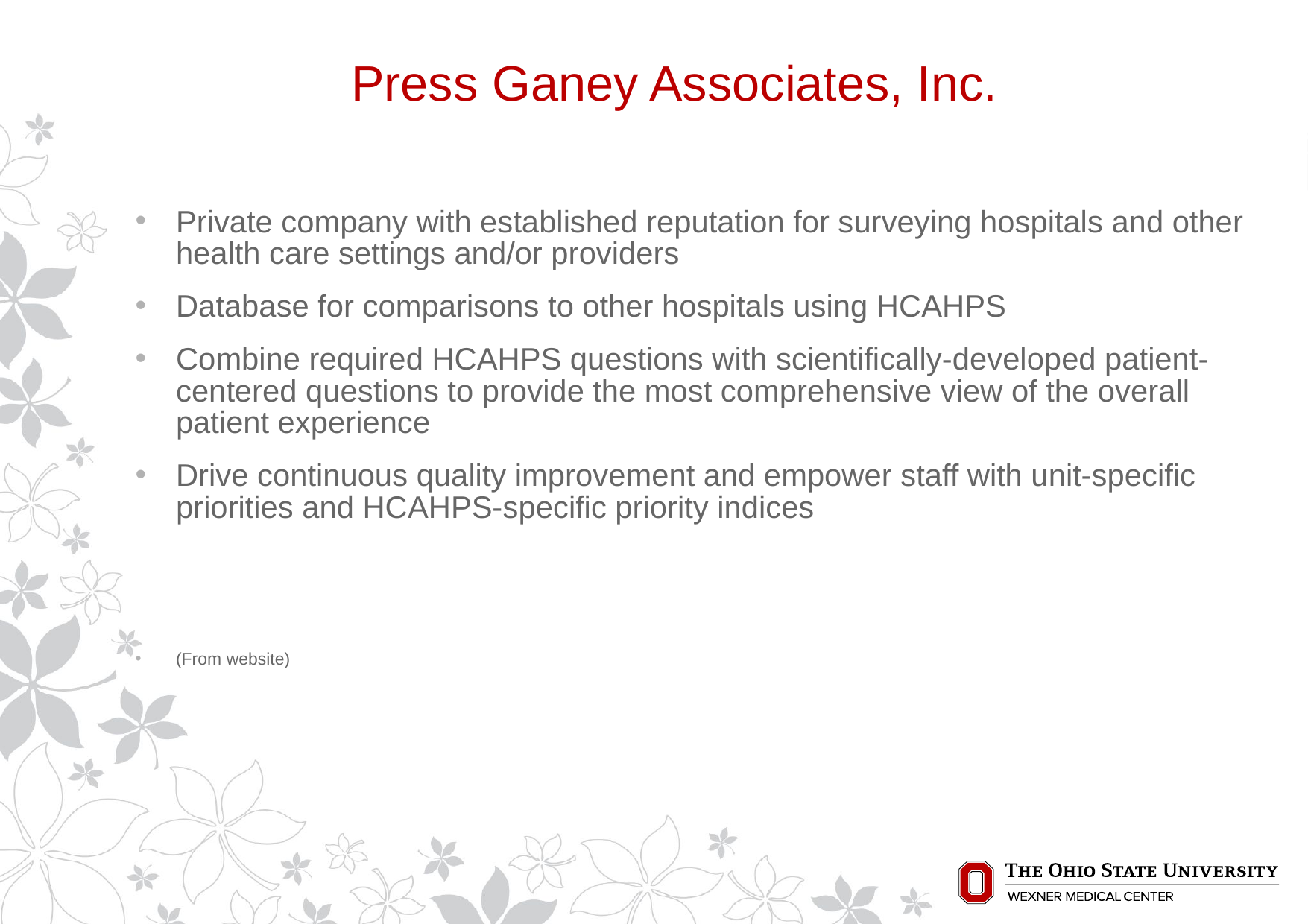

# Press Ganey Associates, Inc.
Private company with established reputation for surveying hospitals and other health care settings and/or providers
Database for comparisons to other hospitals using HCAHPS
Combine required HCAHPS questions with scientifically-developed patient-centered questions to provide the most comprehensive view of the overall patient experience
Drive continuous quality improvement and empower staff with unit-specific priorities and HCAHPS-specific priority indices
(From website)

## Slide 25
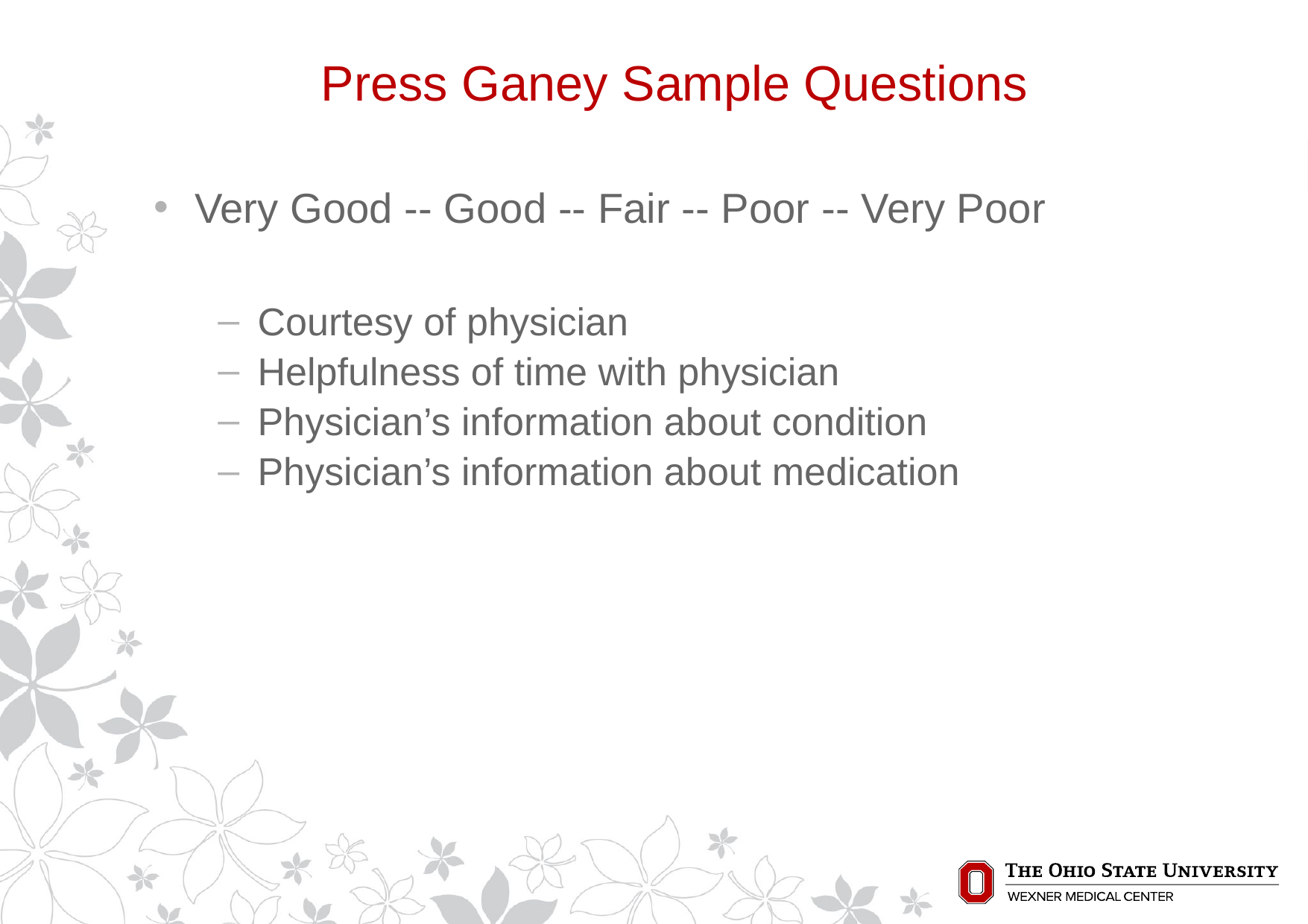

# Press Ganey Sample Questions
Very Good -- Good -- Fair -- Poor -- Very Poor
Courtesy of physician
Helpfulness of time with physician
Physician’s information about condition
Physician’s information about medication

## Slide 26
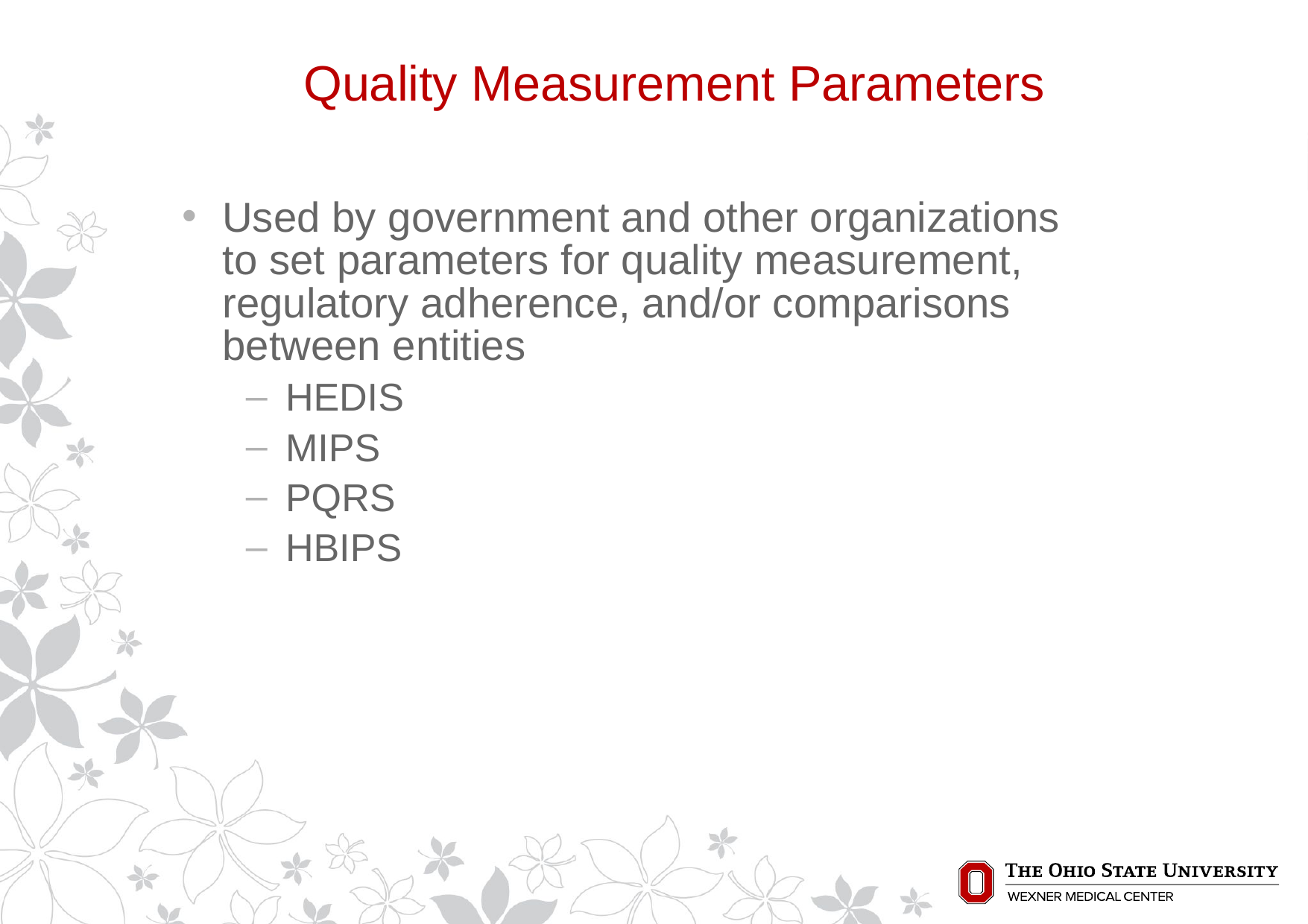

# Quality Measurement Parameters
Used by government and other organizations to set parameters for quality measurement, regulatory adherence, and/or comparisons between entities
HEDIS
MIPS
PQRS
HBIPS

## Slide 27
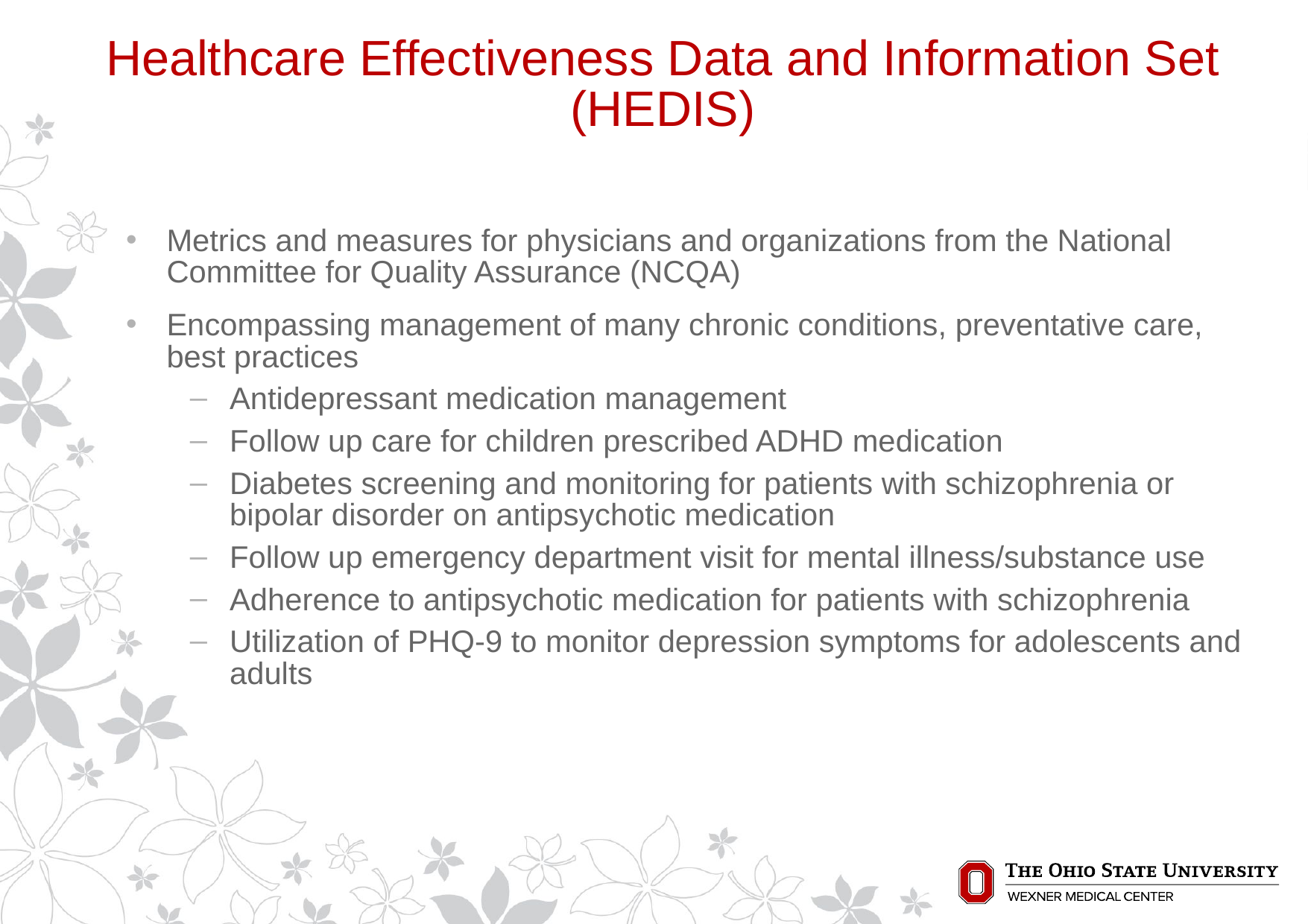

# Healthcare Effectiveness Data and Information Set (HEDIS)
Metrics and measures for physicians and organizations from the National Committee for Quality Assurance (NCQA)
Encompassing management of many chronic conditions, preventative care, best practices
Antidepressant medication management
Follow up care for children prescribed ADHD medication
Diabetes screening and monitoring for patients with schizophrenia or bipolar disorder on antipsychotic medication
Follow up emergency department visit for mental illness/substance use
Adherence to antipsychotic medication for patients with schizophrenia
Utilization of PHQ-9 to monitor depression symptoms for adolescents and adults

## Slide 28
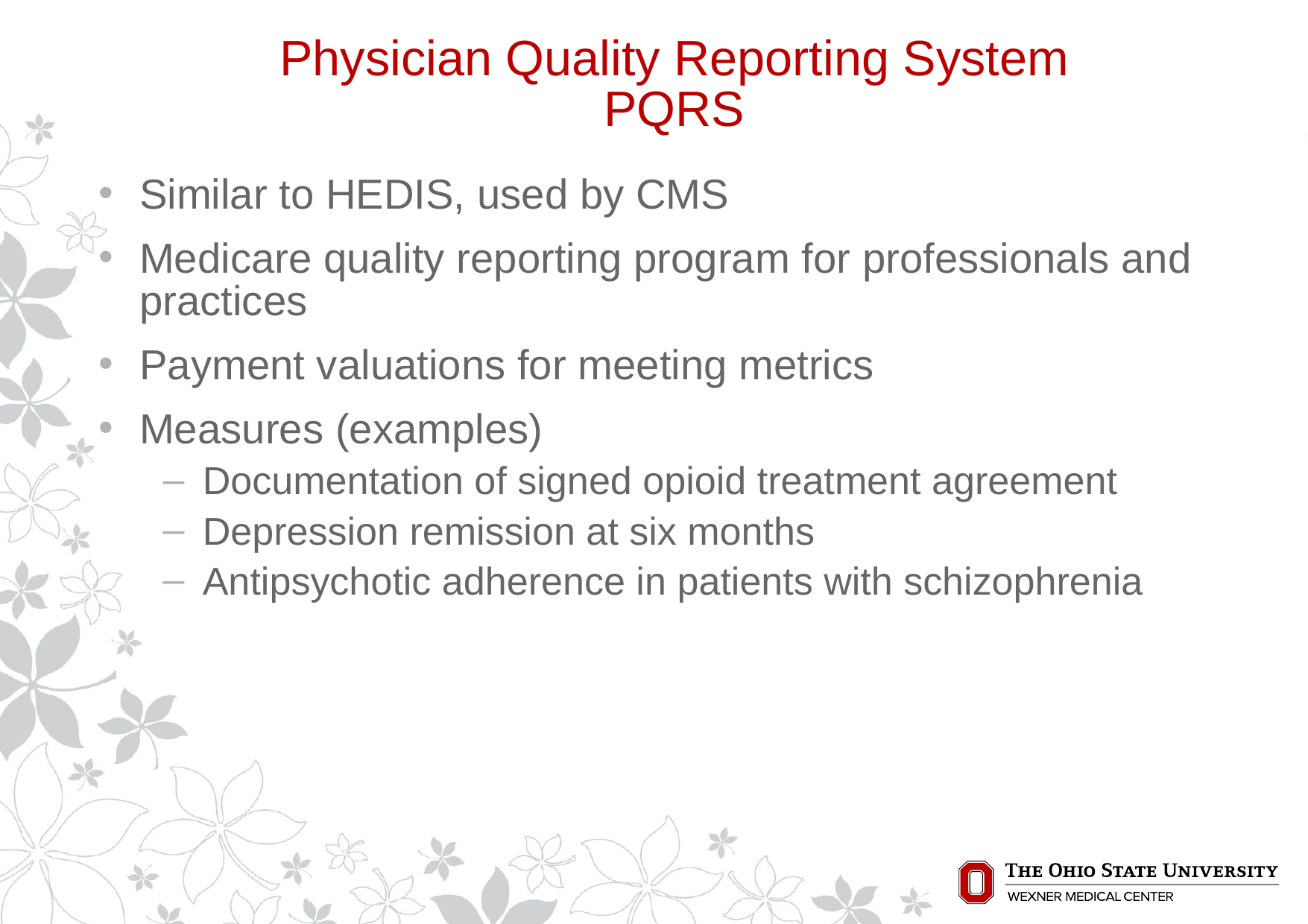

# Physician Quality Reporting System PQRS
Similar to HEDIS, used by CMS
Medicare quality reporting program for professionals and practices
Payment valuations for meeting metrics
Measures (examples)
Documentation of signed opioid treatment agreement
Depression remission at six months
Antipsychotic adherence in patients with schizophrenia

## Slide 29
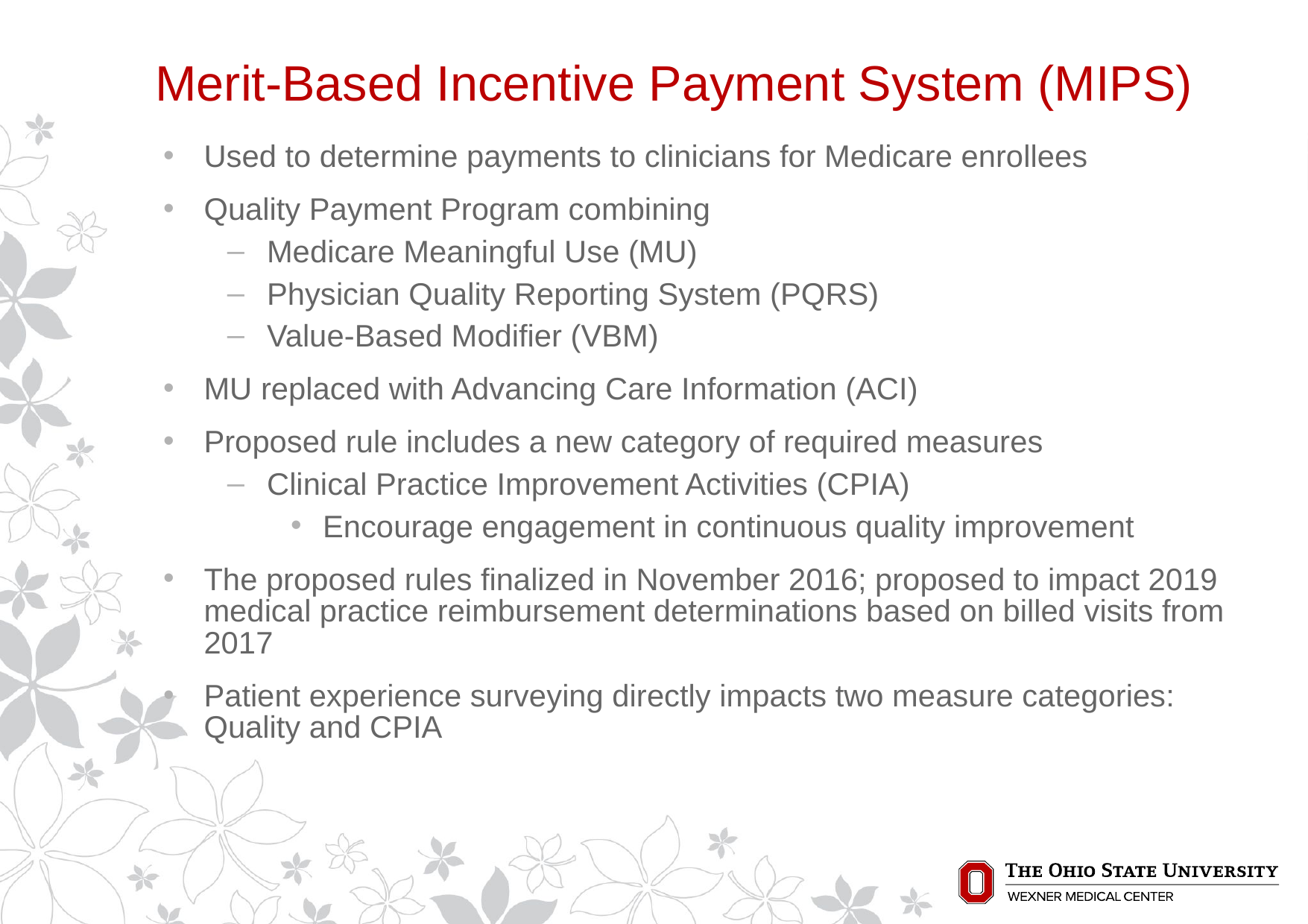

# Merit-Based Incentive Payment System (MIPS)
Used to determine payments to clinicians for Medicare enrollees
Quality Payment Program combining
Medicare Meaningful Use (MU)
Physician Quality Reporting System (PQRS)
Value-Based Modifier (VBM)
MU replaced with Advancing Care Information (ACI)
Proposed rule includes a new category of required measures
Clinical Practice Improvement Activities (CPIA)
Encourage engagement in continuous quality improvement
The proposed rules finalized in November 2016; proposed to impact 2019 medical practice reimbursement determinations based on billed visits from 2017
Patient experience surveying directly impacts two measure categories: Quality and CPIA

## Slide 30
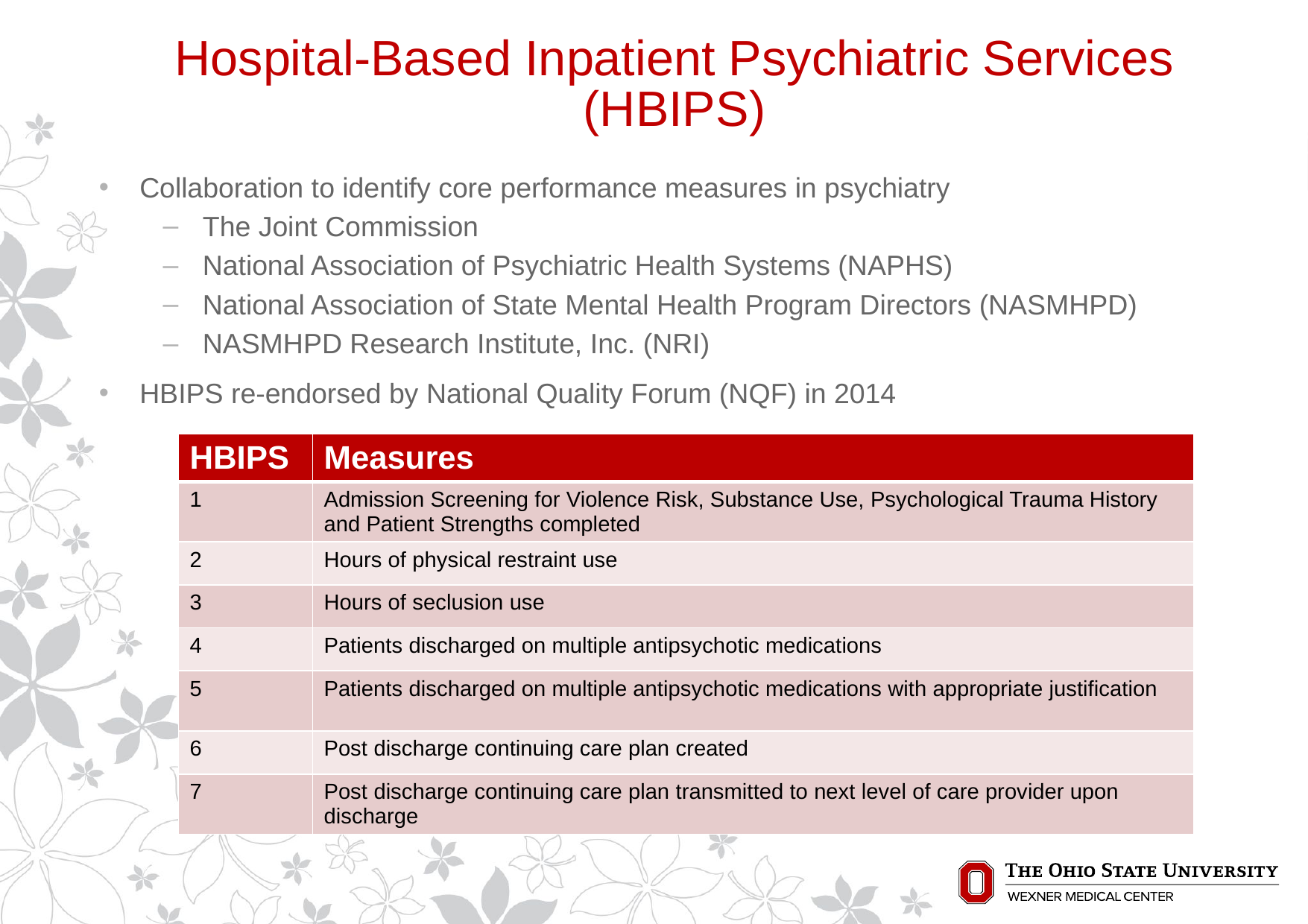

# Hospital-Based Inpatient Psychiatric Services (HBIPS)
Collaboration to identify core performance measures in psychiatry
The Joint Commission
National Association of Psychiatric Health Systems (NAPHS)
National Association of State Mental Health Program Directors (NASMHPD)
NASMHPD Research Institute, Inc. (NRI)
HBIPS re-endorsed by National Quality Forum (NQF) in 2014
| HBIPS | Measures |
| --- | --- |
| 1 | Admission Screening for Violence Risk, Substance Use, Psychological Trauma History and Patient Strengths completed |
| 2 | Hours of physical restraint use |
| 3 | Hours of seclusion use |
| 4 | Patients discharged on multiple antipsychotic medications |
| 5 | Patients discharged on multiple antipsychotic medications with appropriate justification |
| 6 | Post discharge continuing care plan created |
| 7 | Post discharge continuing care plan transmitted to next level of care provider upon discharge |

## Slide 31
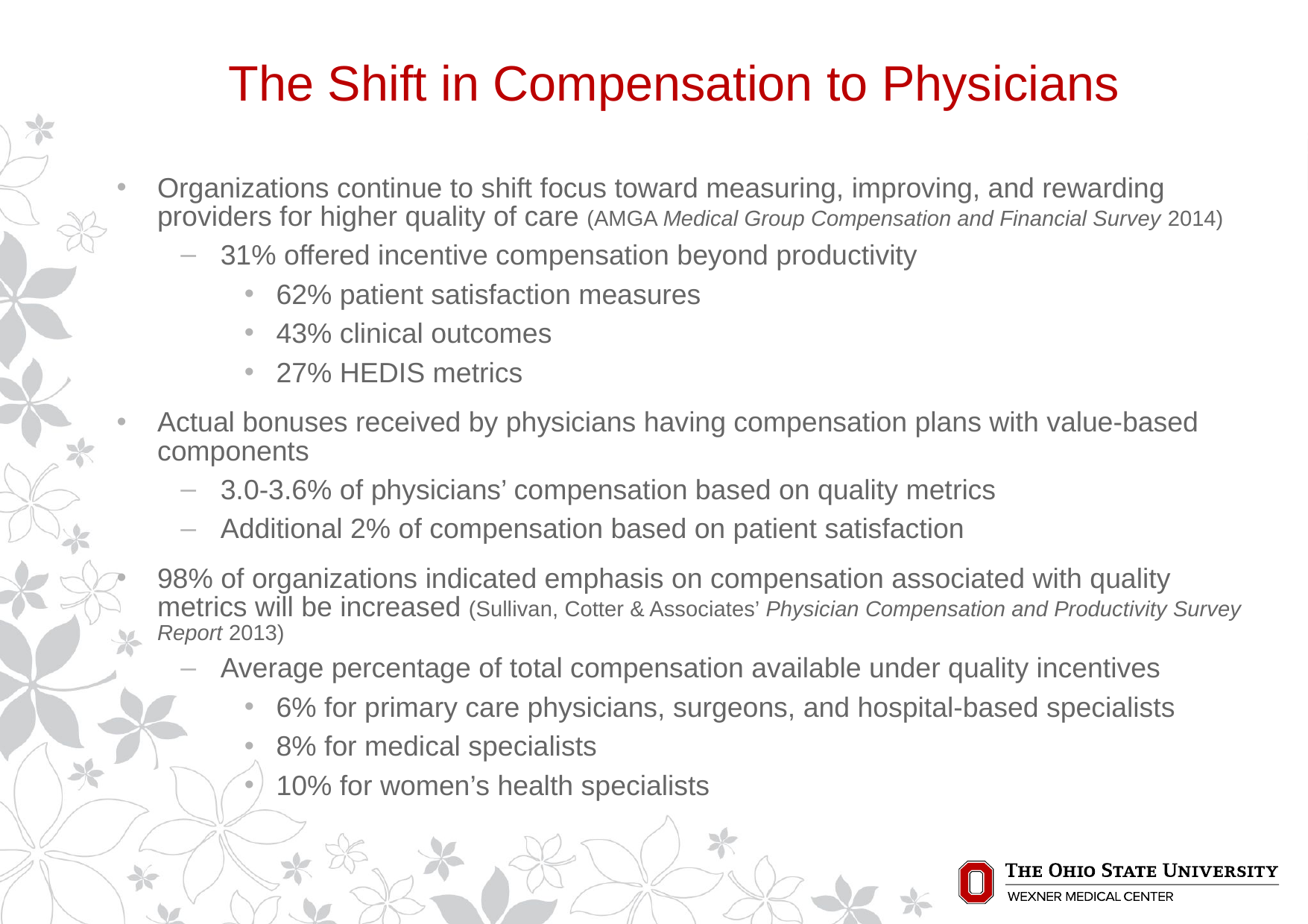

# The Shift in Compensation to Physicians
Organizations continue to shift focus toward measuring, improving, and rewarding providers for higher quality of care (AMGA Medical Group Compensation and Financial Survey 2014)
31% offered incentive compensation beyond productivity
62% patient satisfaction measures
43% clinical outcomes
27% HEDIS metrics
Actual bonuses received by physicians having compensation plans with value-based components
3.0-3.6% of physicians’ compensation based on quality metrics
Additional 2% of compensation based on patient satisfaction
98% of organizations indicated emphasis on compensation associated with quality metrics will be increased (Sullivan, Cotter & Associates’ Physician Compensation and Productivity Survey Report 2013)
Average percentage of total compensation available under quality incentives
6% for primary care physicians, surgeons, and hospital-based specialists
8% for medical specialists
10% for women’s health specialists

## Slide 32
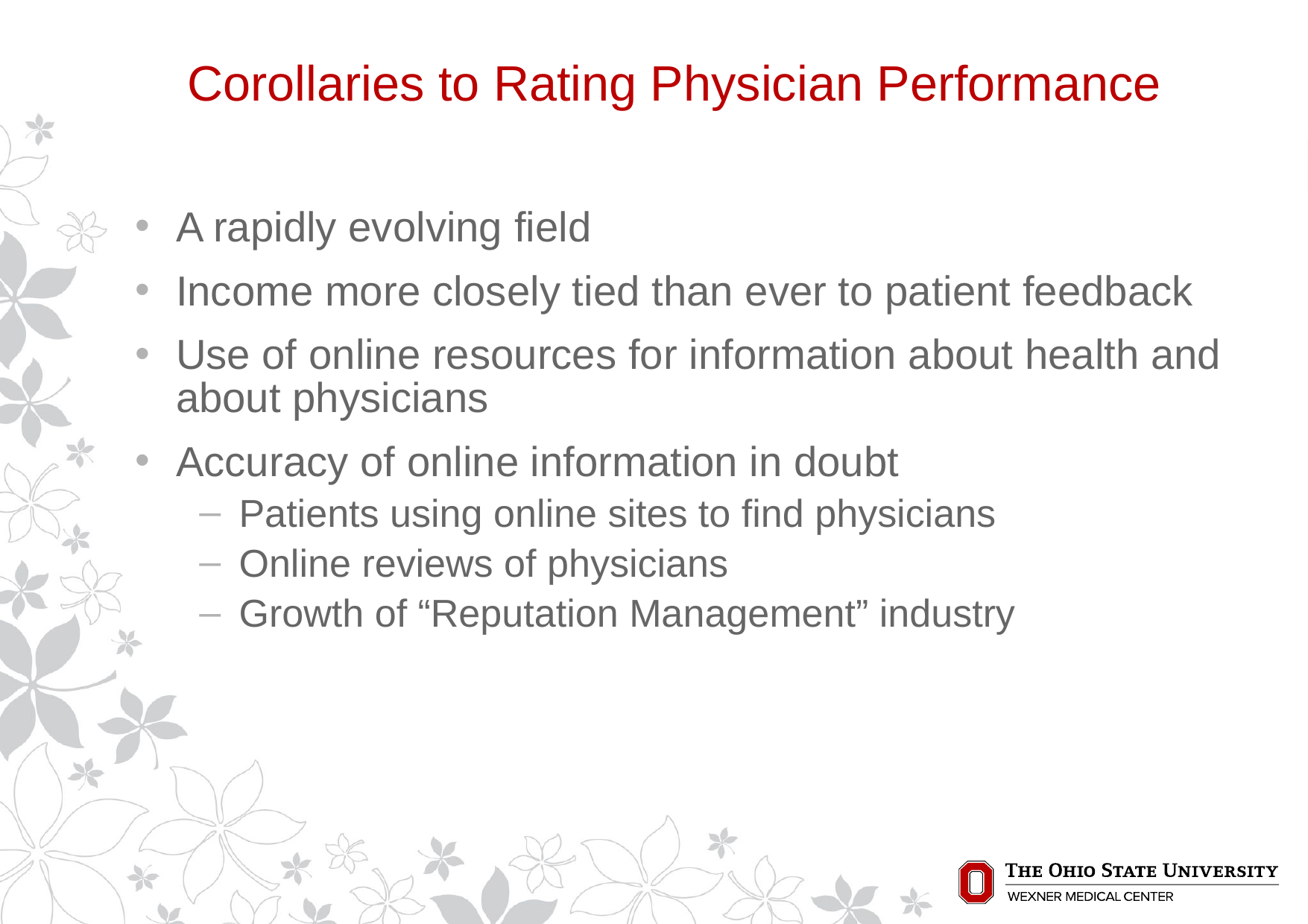

# Corollaries to Rating Physician Performance
A rapidly evolving field
Income more closely tied than ever to patient feedback
Use of online resources for information about health and about physicians
Accuracy of online information in doubt
Patients using online sites to find physicians
Online reviews of physicians
Growth of “Reputation Management” industry

## Slide 33
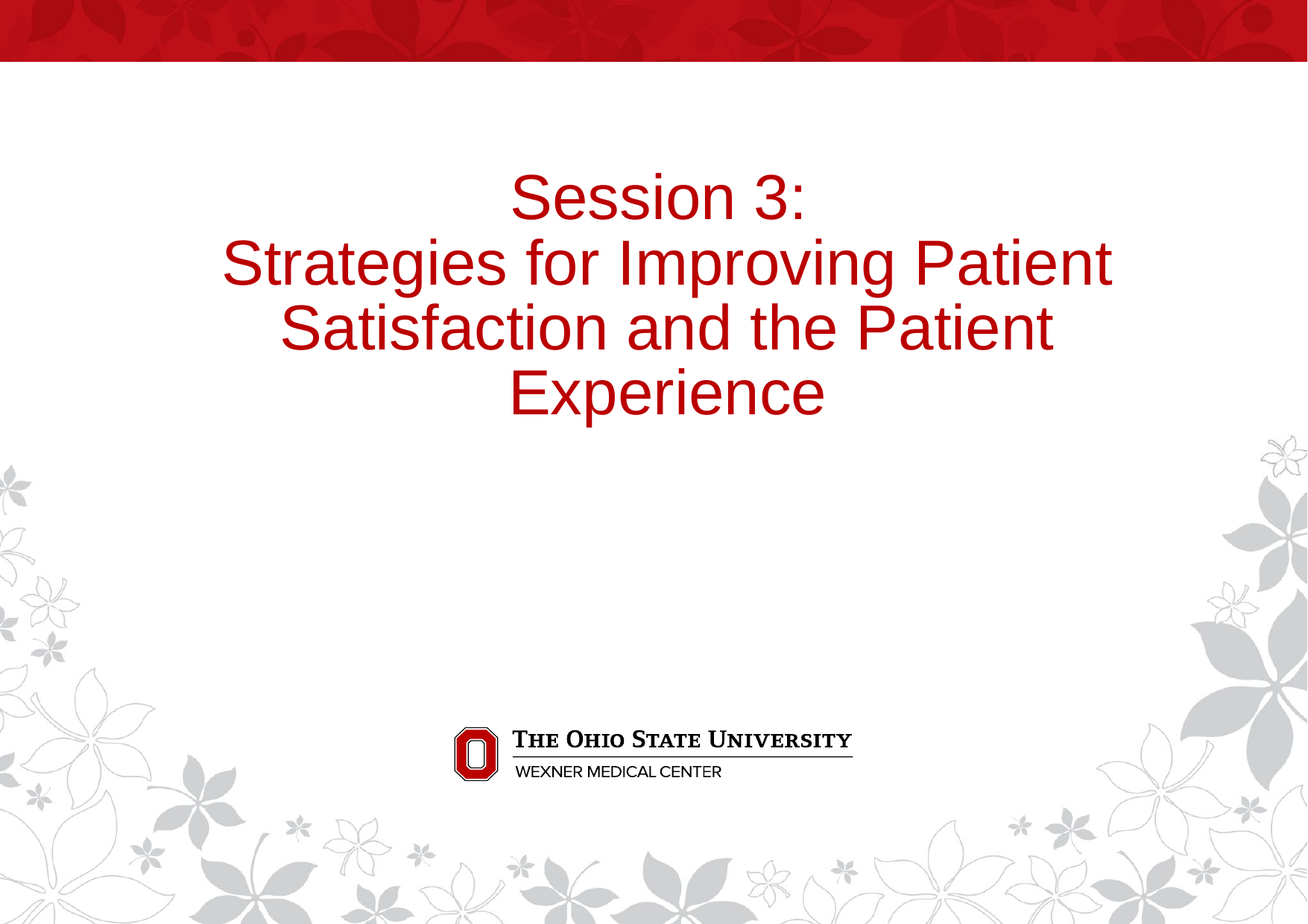

# Session 3: Strategies for Improving Patient Satisfaction and the Patient Experience

## Slide 34
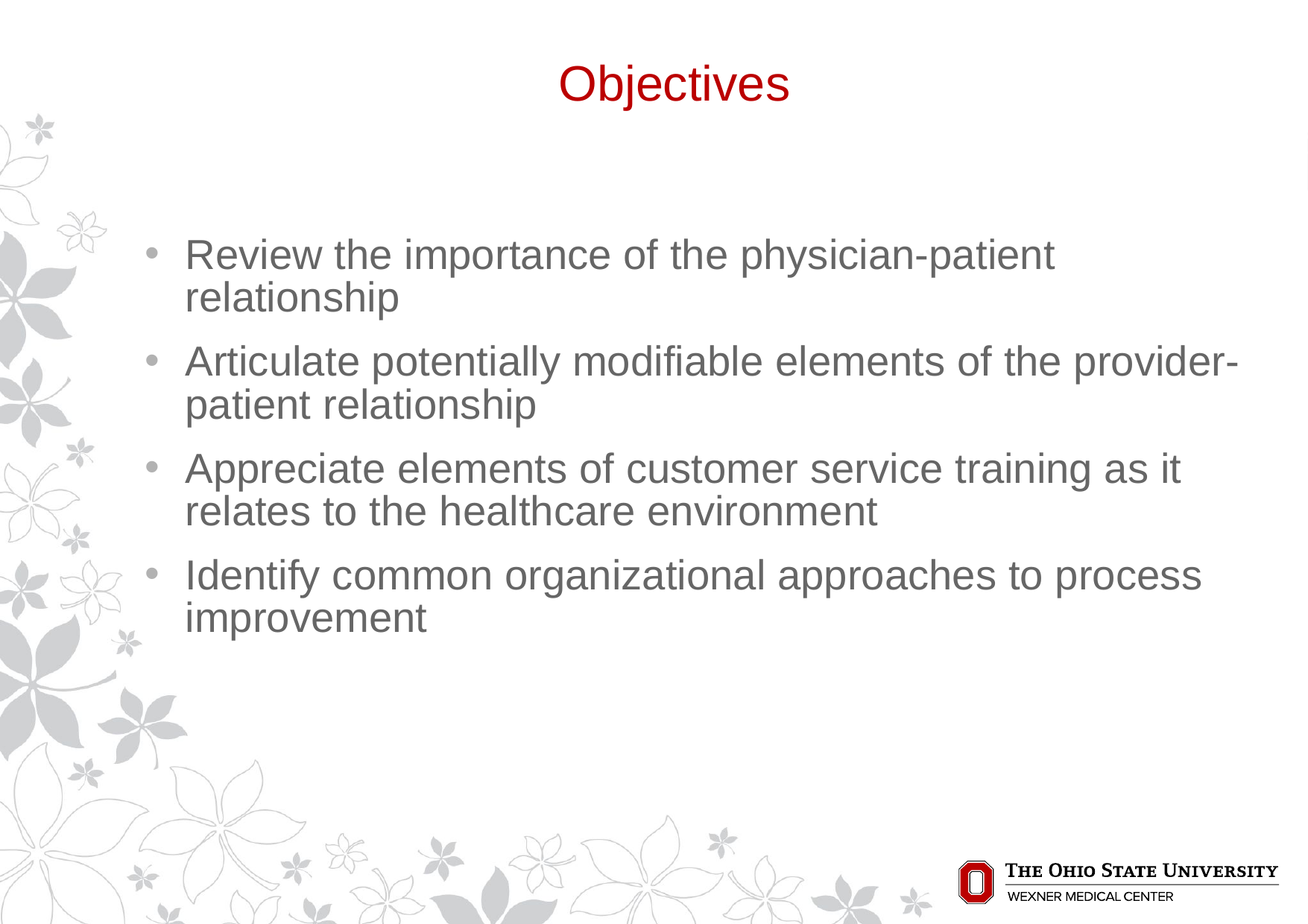

# Objectives
Review the importance of the physician-patient relationship
Articulate potentially modifiable elements of the provider-patient relationship
Appreciate elements of customer service training as it relates to the healthcare environment
Identify common organizational approaches to process improvement

## Slide 35
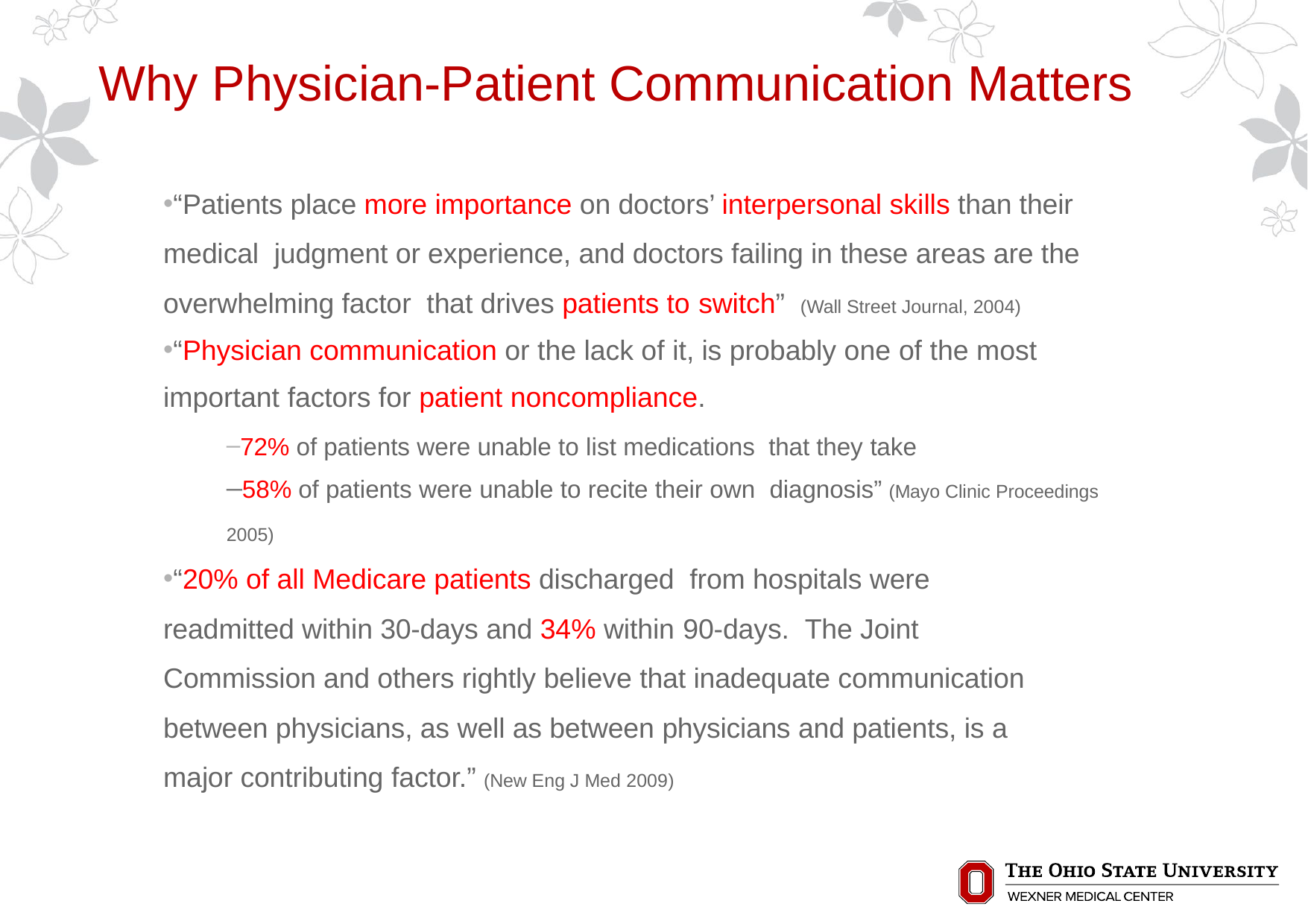

# Why Physician-Patient Communication Matters
“Patients place more importance on doctors’ interpersonal skills than their medical judgment or experience, and doctors failing in these areas are the overwhelming factor that drives patients to switch” (Wall Street Journal, 2004)
“Physician communication or the lack of it, is probably one of the most important factors for patient noncompliance.
72% of patients were unable to list medications that they take
58% of patients were unable to recite their own diagnosis” (Mayo Clinic Proceedings 2005)
“20% of all Medicare patients discharged from hospitals were readmitted within 30-days and 34% within 90-days. The Joint Commission and others rightly believe that inadequate communication between physicians, as well as between physicians and patients, is a major contributing factor.” (New Eng J Med 2009)

## Slide 36
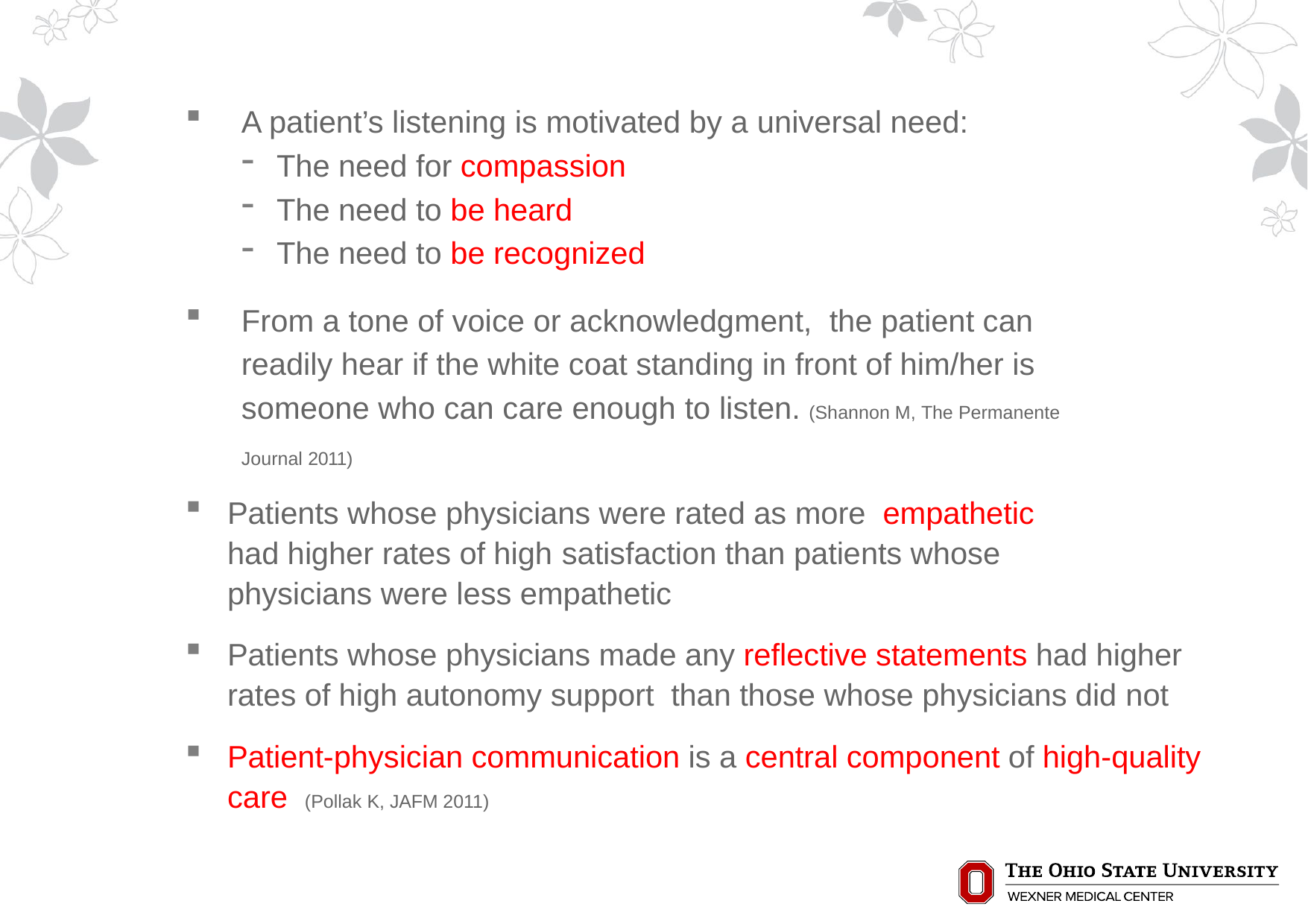

A patient’s listening is motivated by a universal need:
The need for compassion
The need to be heard
The need to be recognized
From a tone of voice or acknowledgment, the patient can readily hear if the white coat standing in front of him/her is someone who can care enough to listen. (Shannon M, The Permanente Journal 2011)
Patients whose physicians were rated as more empathetic had higher rates of high satisfaction than patients whose physicians were less empathetic
Patients whose physicians made any reflective statements had higher rates of high autonomy support than those whose physicians did not
Patient-physician communication is a central component of high-quality care (Pollak K, JAFM 2011)

## Slide 37
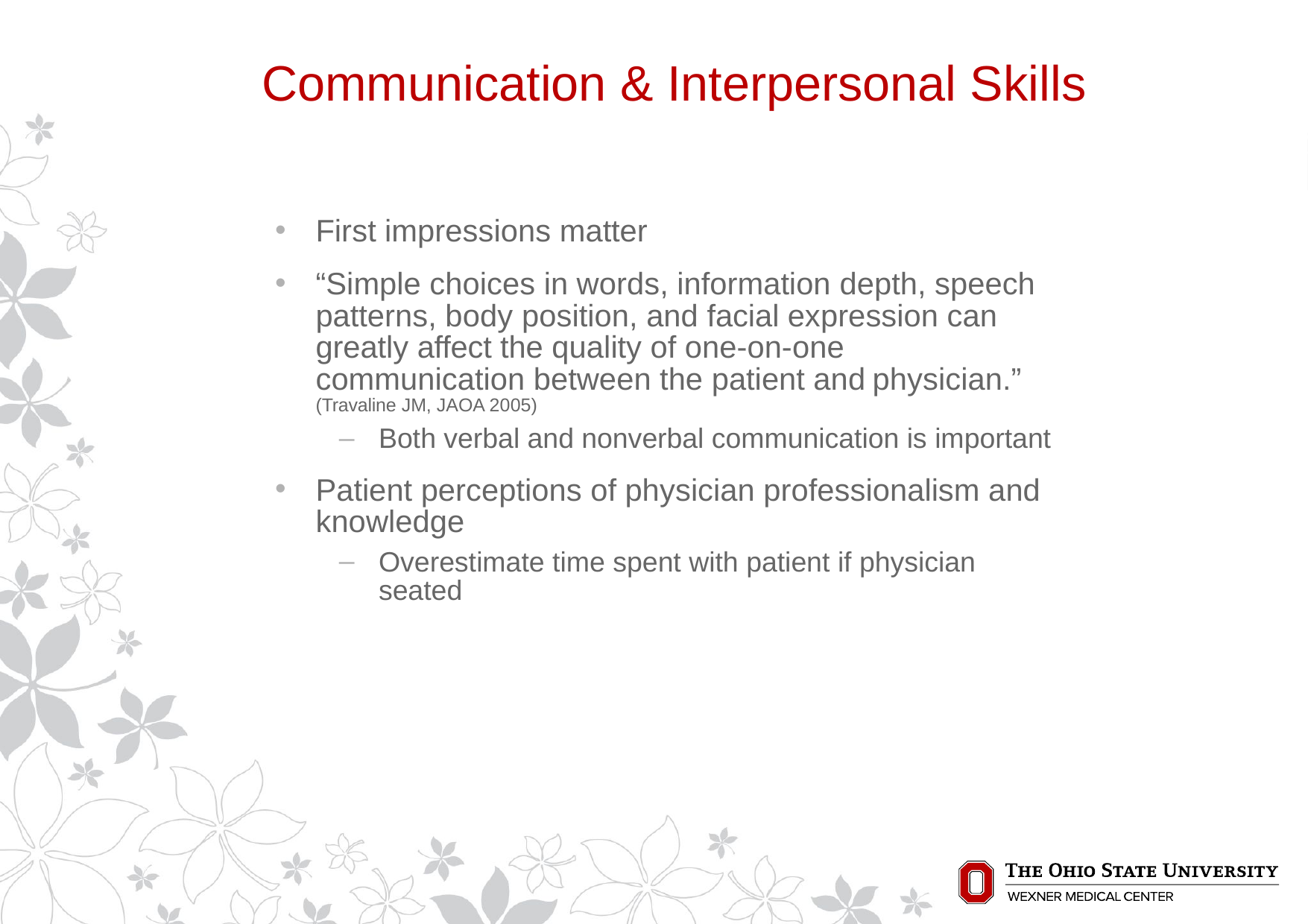

# Communication & Interpersonal Skills
First impressions matter
“Simple choices in words, information depth, speech patterns, body position, and facial expression can greatly affect the quality of one-on-one communication between the patient and physician.” (Travaline JM, JAOA 2005)
Both verbal and nonverbal communication is important
Patient perceptions of physician professionalism and knowledge
Overestimate time spent with patient if physician seated

## Slide 38
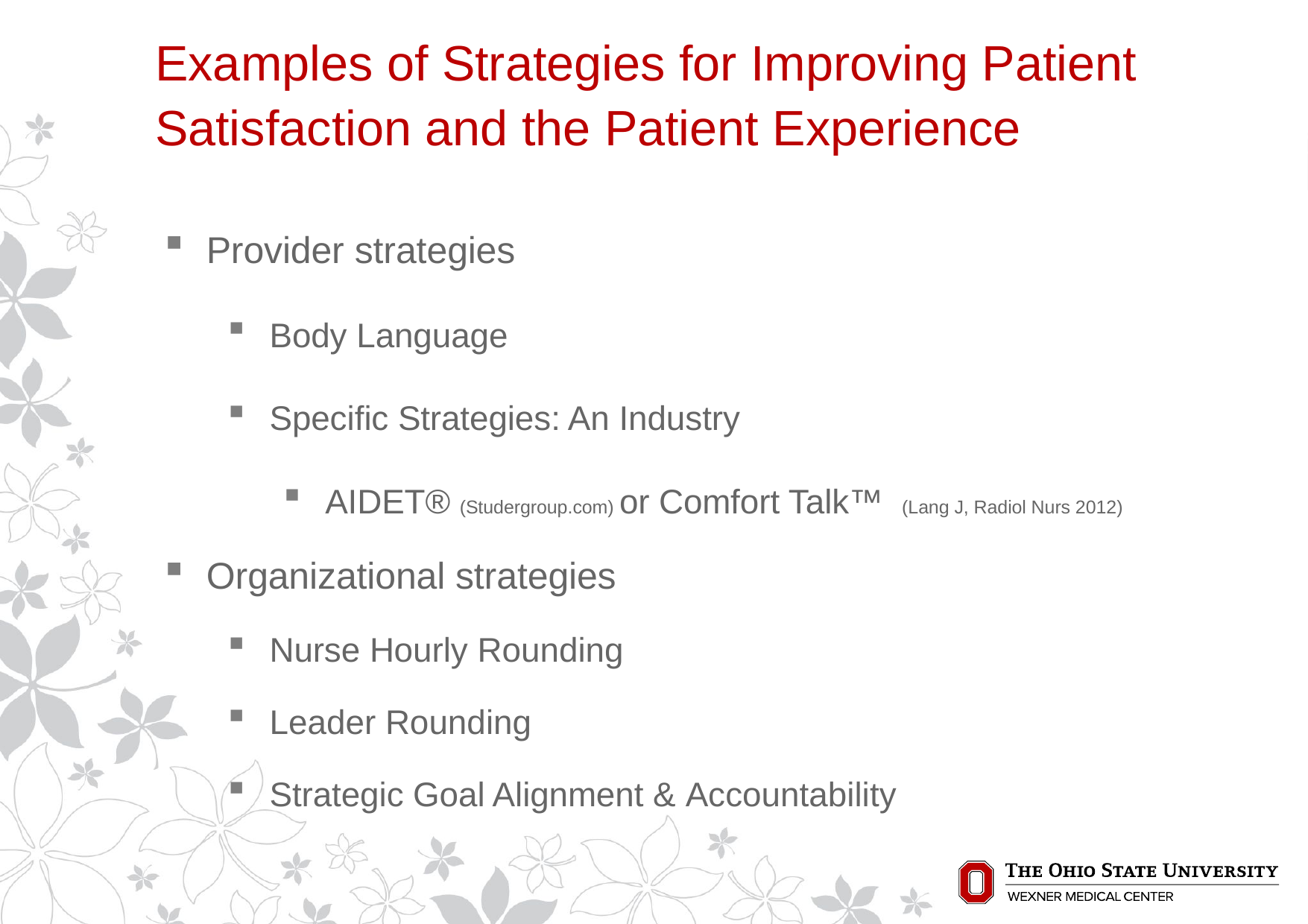

# Examples of Strategies for Improving Patient Satisfaction and the Patient Experience
Provider strategies
Body Language
Specific Strategies: An Industry
AIDET® (Studergroup.com) or Comfort Talk™ (Lang J, Radiol Nurs 2012)
Organizational strategies
Nurse Hourly Rounding
Leader Rounding
Strategic Goal Alignment & Accountability

## Slide 39
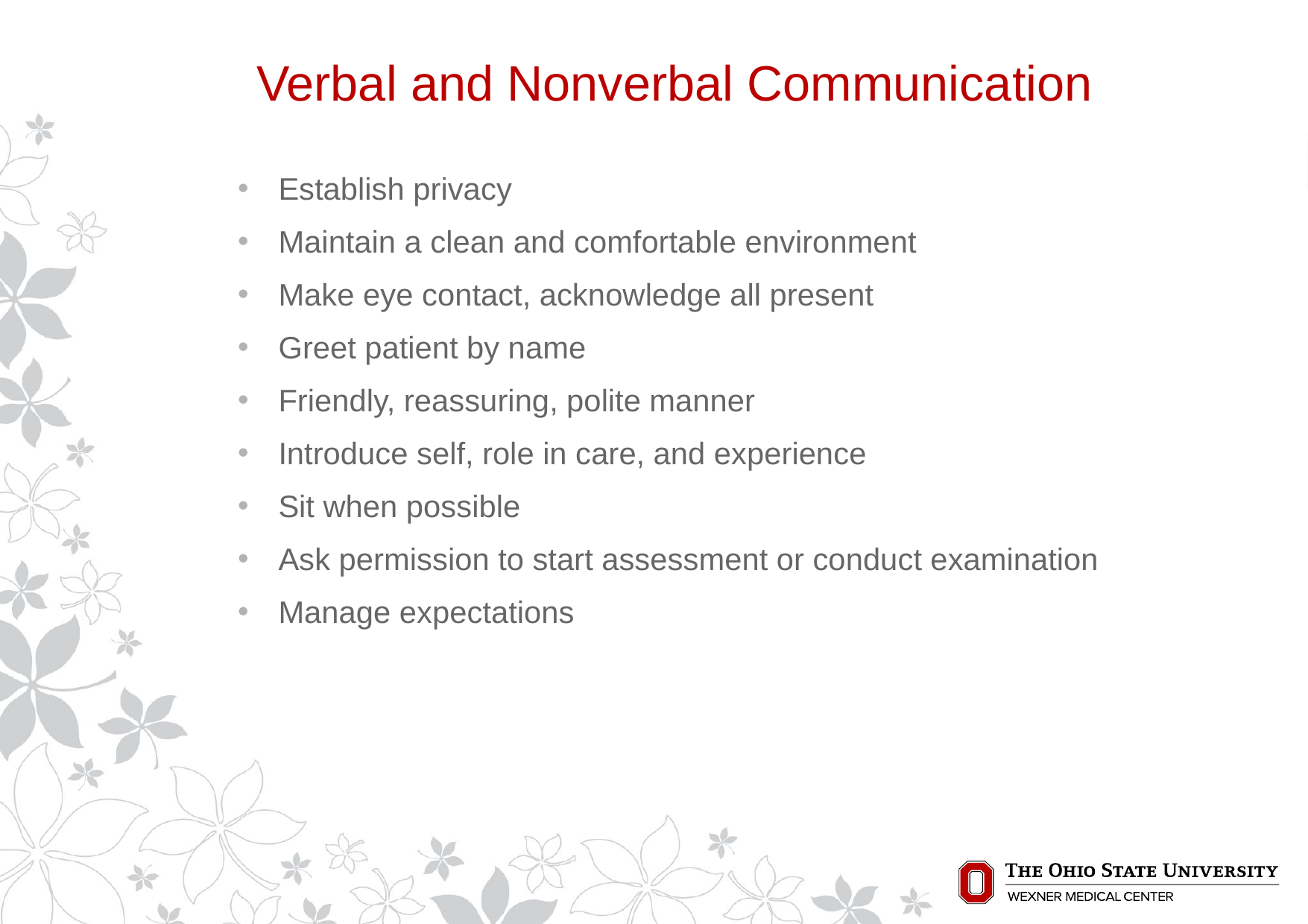

# Verbal and Nonverbal Communication
Establish privacy
Maintain a clean and comfortable environment
Make eye contact, acknowledge all present
Greet patient by name
Friendly, reassuring, polite manner
Introduce self, role in care, and experience
Sit when possible
Ask permission to start assessment or conduct examination
Manage expectations

## Slide 40
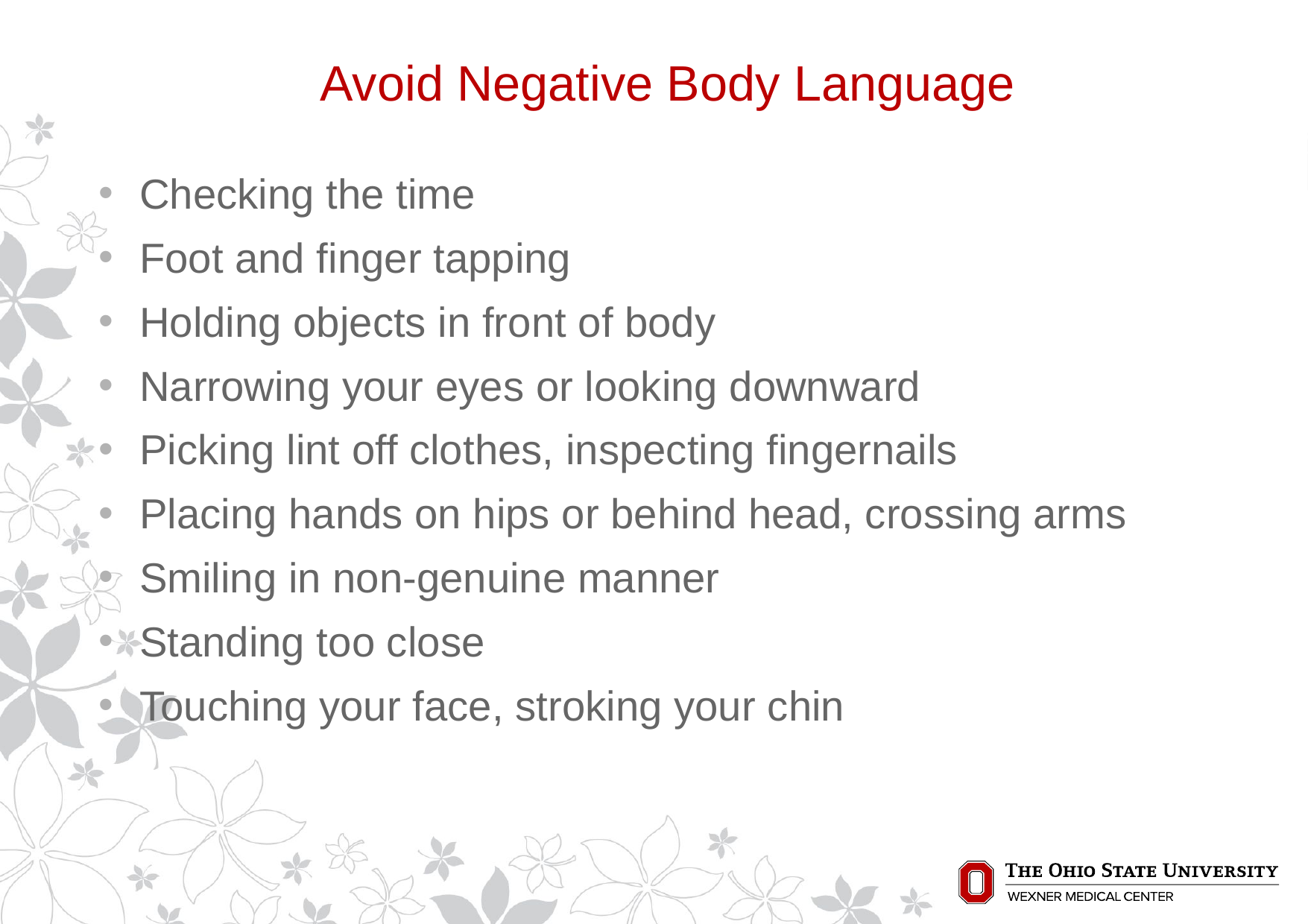

# Avoid Negative Body Language
Checking the time
Foot and finger tapping
Holding objects in front of body
Narrowing your eyes or looking downward
Picking lint off clothes, inspecting fingernails
Placing hands on hips or behind head, crossing arms
Smiling in non-genuine manner
Standing too close
Touching your face, stroking your chin

## Slide 41
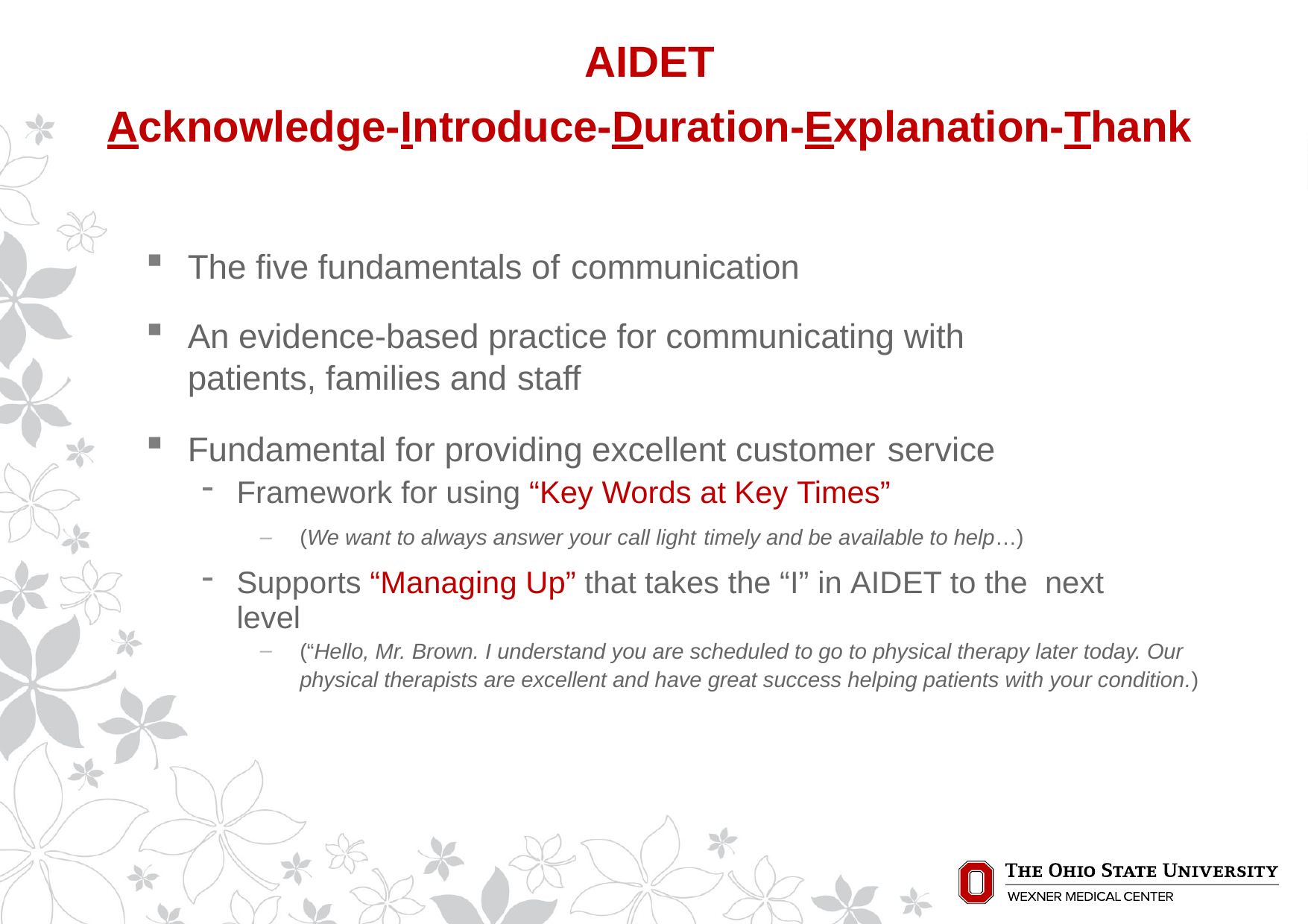

# AIDET Acknowledge-Introduce-Duration-Explanation-Thank
The five fundamentals of communication
An evidence-based practice for communicating with patients, families and staff
Fundamental for providing excellent customer service
Framework for using “Key Words at Key Times”
(We want to always answer your call light timely and be available to help…)
Supports “Managing Up” that takes the “I” in AIDET to the next level
(“Hello, Mr. Brown. I understand you are scheduled to go to physical therapy later today. Our physical therapists are excellent and have great success helping patients with your condition.)

## Slide 42
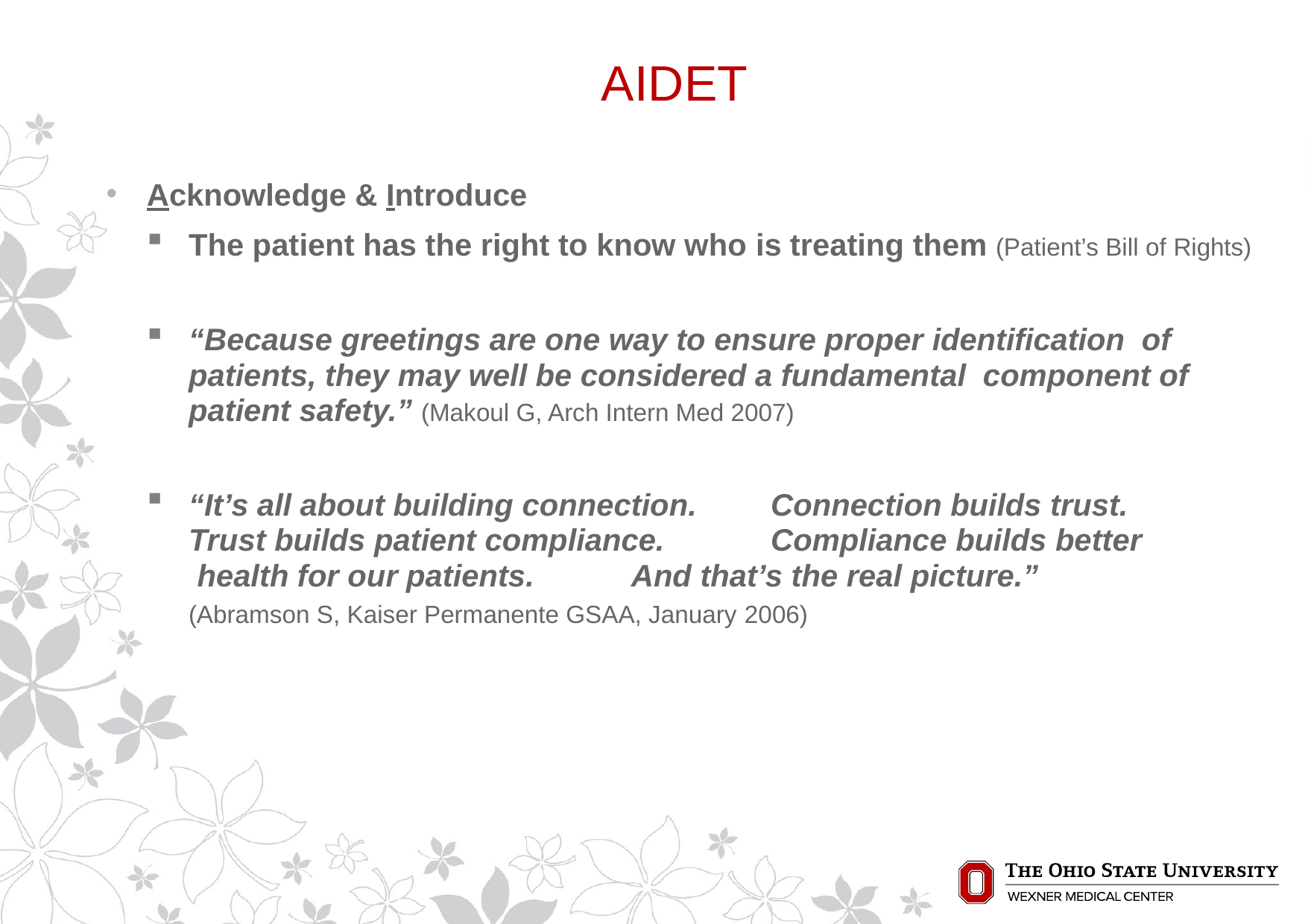

# AIDET
Acknowledge & Introduce
The patient has the right to know who is treating them (Patient’s Bill of Rights)
“Because greetings are one way to ensure proper identification of patients, they may well be considered a fundamental component of patient safety.” (Makoul G, Arch Intern Med 2007)
“It’s all about building connection.	Connection builds trust. Trust builds patient compliance.	Compliance builds better health for our patients.	And that’s the real picture.” (Abramson S, Kaiser Permanente GSAA, January 2006)

## Slide 43
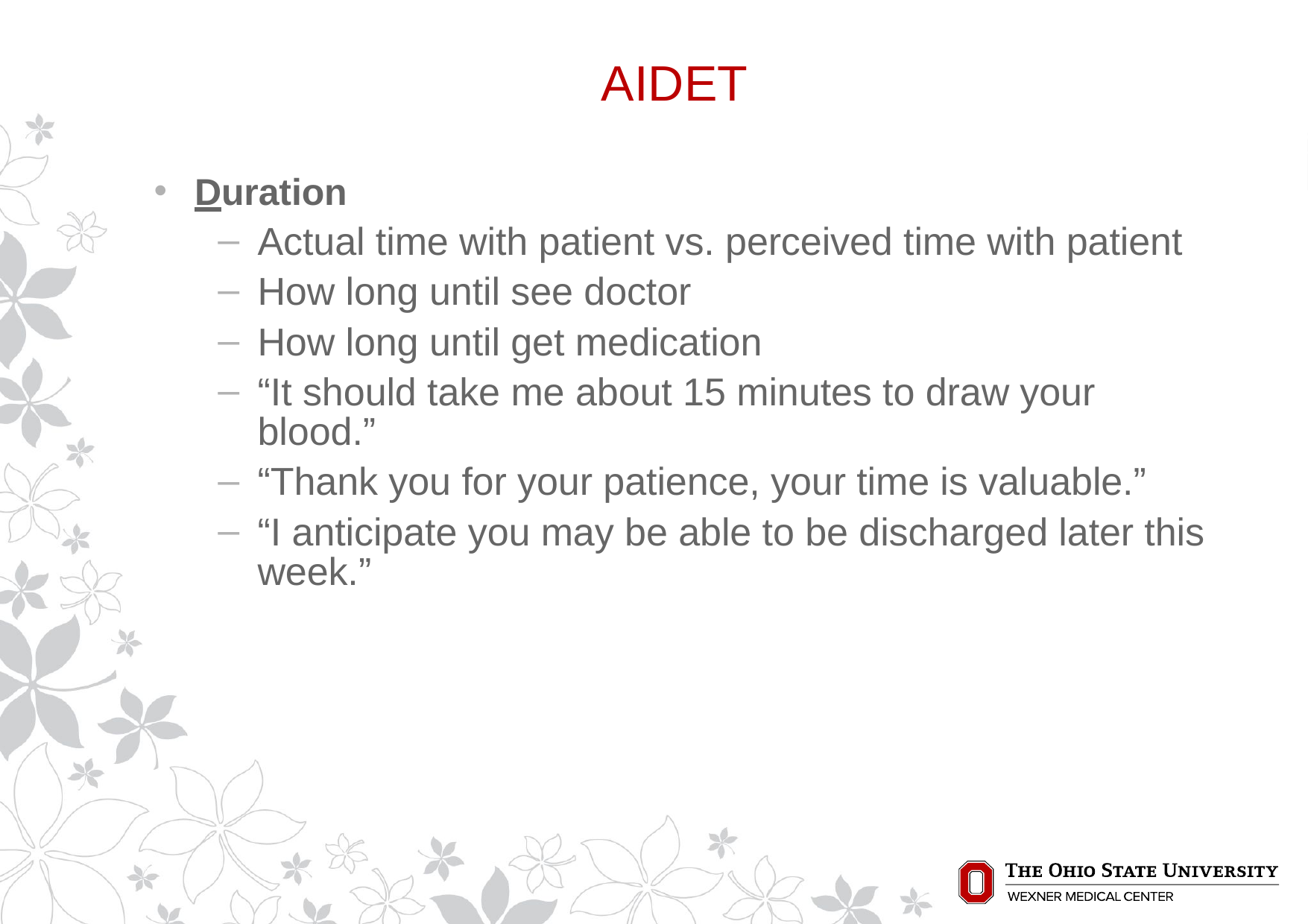

# AIDET
Duration
Actual time with patient vs. perceived time with patient
How long until see doctor
How long until get medication
“It should take me about 15 minutes to draw your blood.”
“Thank you for your patience, your time is valuable.”
“I anticipate you may be able to be discharged later this week.”

## Slide 44
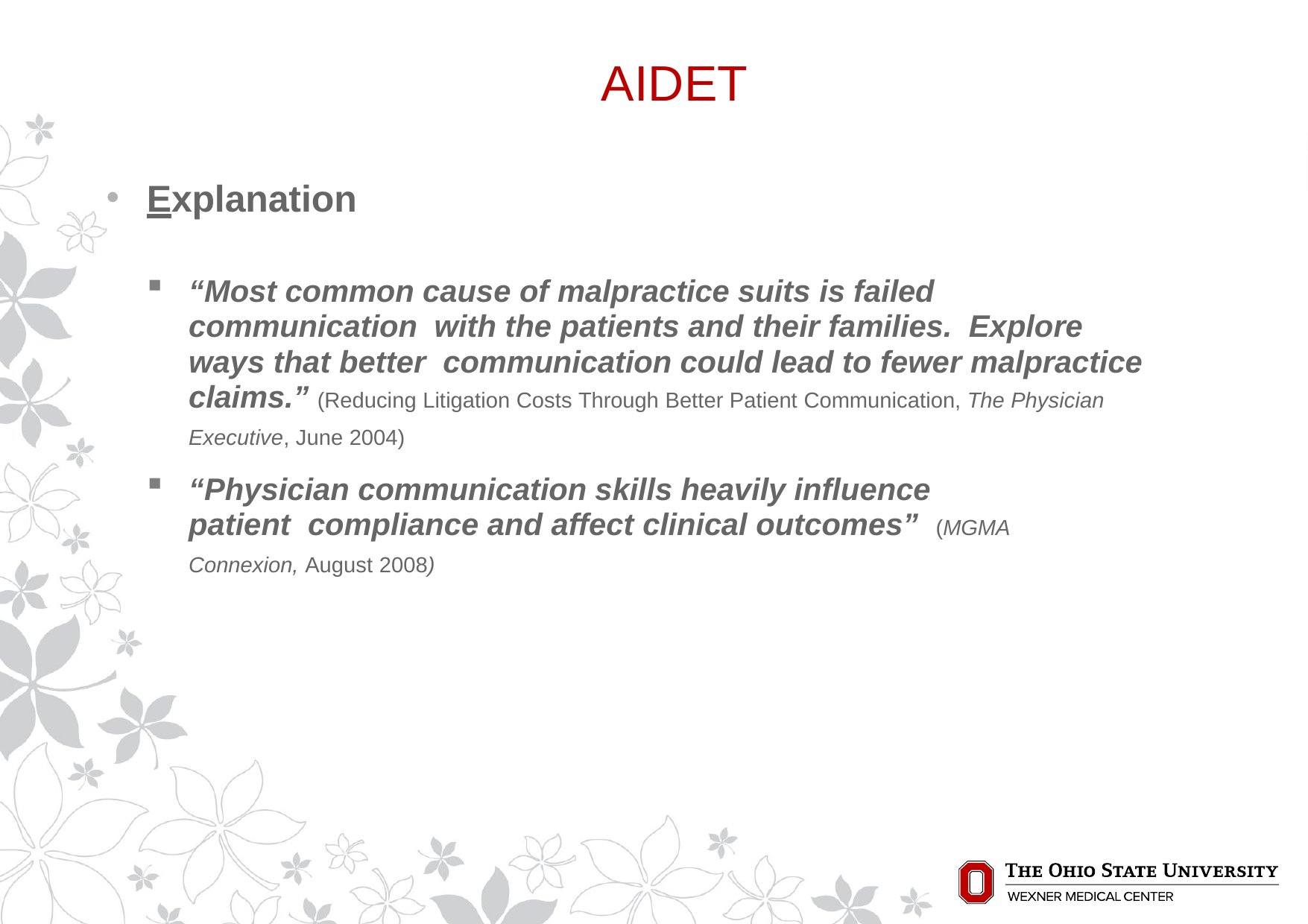

# AIDET
Explanation
“Most common cause of malpractice suits is failed communication with the patients and their families. Explore ways that better communication could lead to fewer malpractice claims.” (Reducing Litigation Costs Through Better Patient Communication, The Physician Executive, June 2004)
“Physician communication skills heavily influence patient compliance and affect clinical outcomes” (MGMA Connexion, August 2008)

## Slide 45
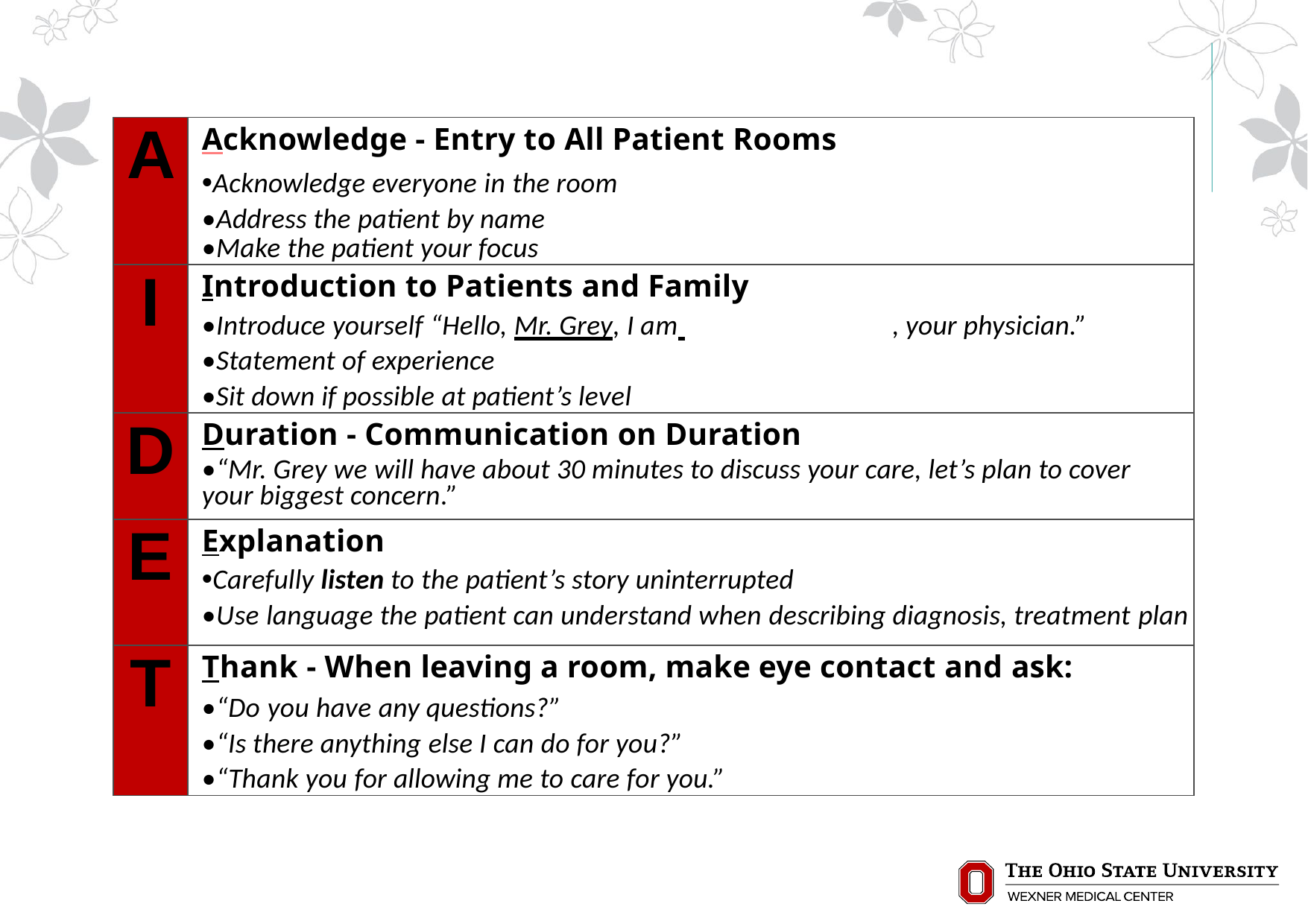

| A | Acknowledge - Entry to All Patient Rooms •Acknowledge everyone in the room •Address the patient by name •Make the patient your focus |
| --- | --- |
| I | Introduction to Patients and Family •Introduce yourself “Hello, Mr. Grey, I am , your physician.” •Statement of experience •Sit down if possible at patient’s level |
| D | Duration - Communication on Duration •“Mr. Grey we will have about 30 minutes to discuss your care, let’s plan to cover your biggest concern.” |
| E | Explanation •Carefully listen to the patient’s story uninterrupted •Use language the patient can understand when describing diagnosis, treatment plan |
| T | Thank - When leaving a room, make eye contact and ask: •“Do you have any questions?” •“Is there anything else I can do for you?” •“Thank you for allowing me to care for you.” |

## Slide 46
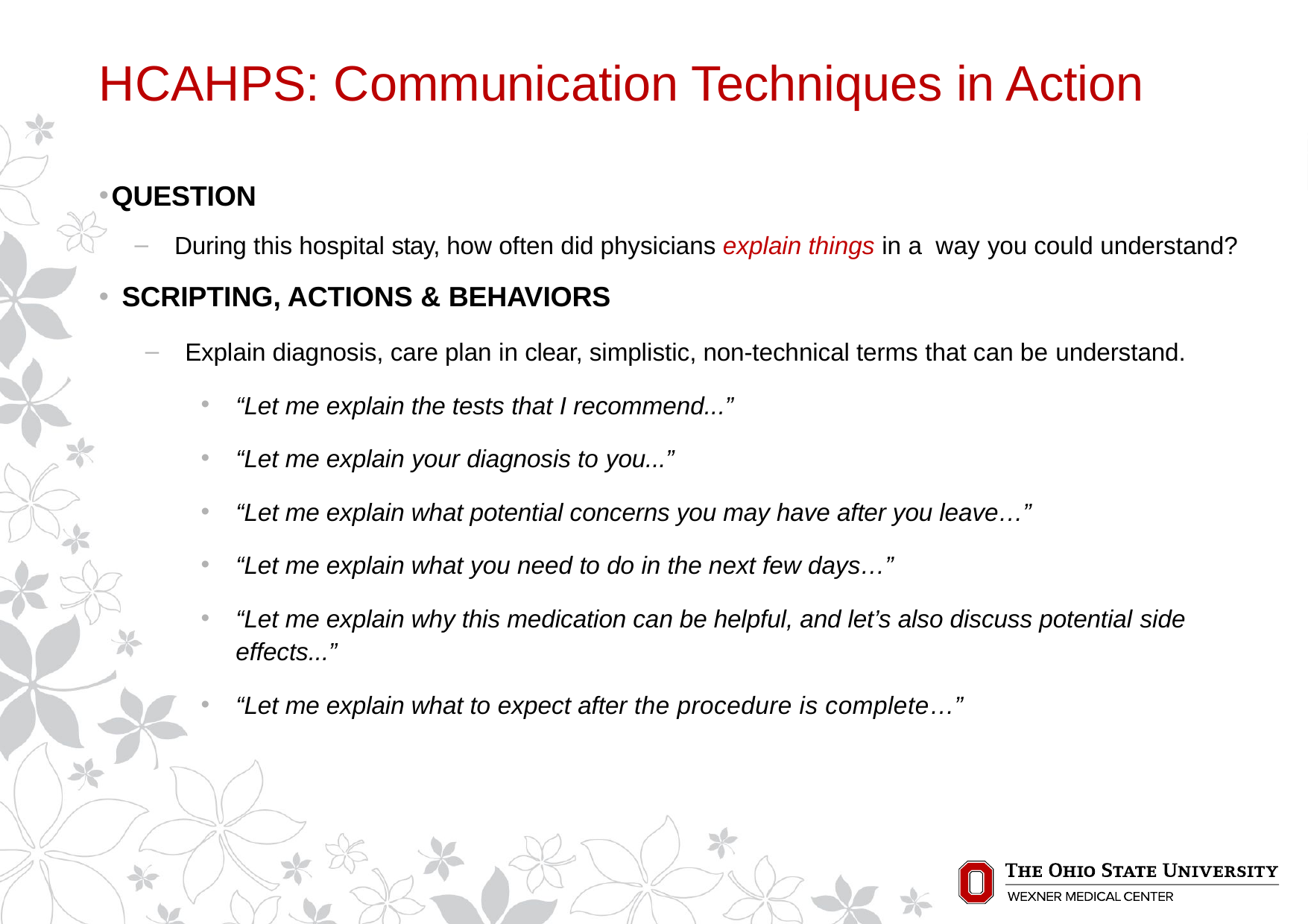

# HCAHPS: Communication Techniques in Action
QUESTION
During this hospital stay, how often did physicians explain things in a way you could understand?
SCRIPTING, ACTIONS & BEHAVIORS
Explain diagnosis, care plan in clear, simplistic, non-technical terms that can be understand.
“Let me explain the tests that I recommend...”
“Let me explain your diagnosis to you...”
“Let me explain what potential concerns you may have after you leave…”
“Let me explain what you need to do in the next few days…”
“Let me explain why this medication can be helpful, and let’s also discuss potential side effects...”
“Let me explain what to expect after the procedure is complete…”

## Slide 47
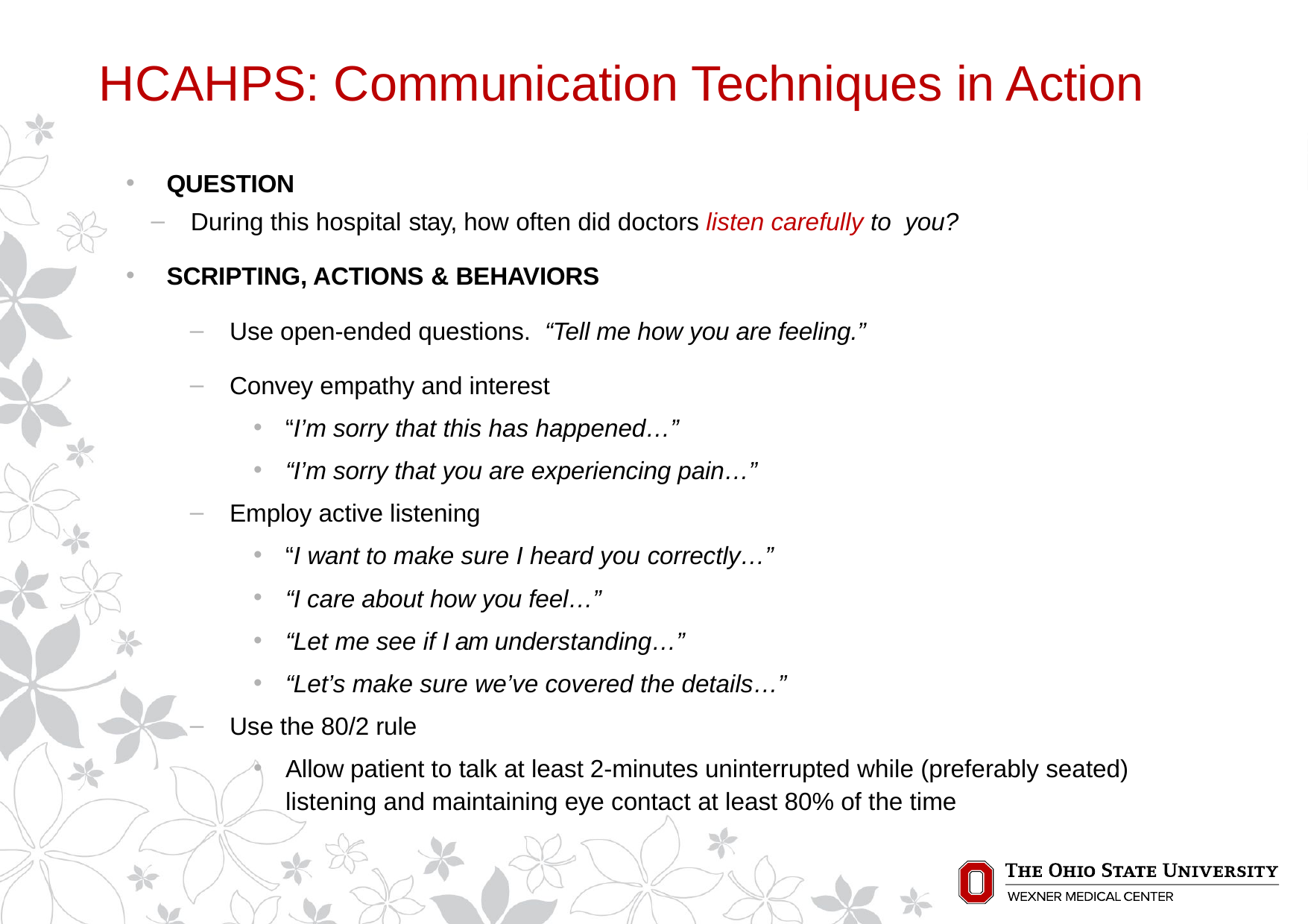

# HCAHPS: Communication Techniques in Action
QUESTION
During this hospital stay, how often did doctors listen carefully to you?
SCRIPTING, ACTIONS & BEHAVIORS
Use open-ended questions. “Tell me how you are feeling.”
Convey empathy and interest
“I’m sorry that this has happened…”
“I’m sorry that you are experiencing pain…”
Employ active listening
“I want to make sure I heard you correctly…”
“I care about how you feel…”
“Let me see if I am understanding…”
“Let’s make sure we’ve covered the details…”
Use the 80/2 rule
Allow patient to talk at least 2-minutes uninterrupted while (preferably seated) listening and maintaining eye contact at least 80% of the time

## Slide 48
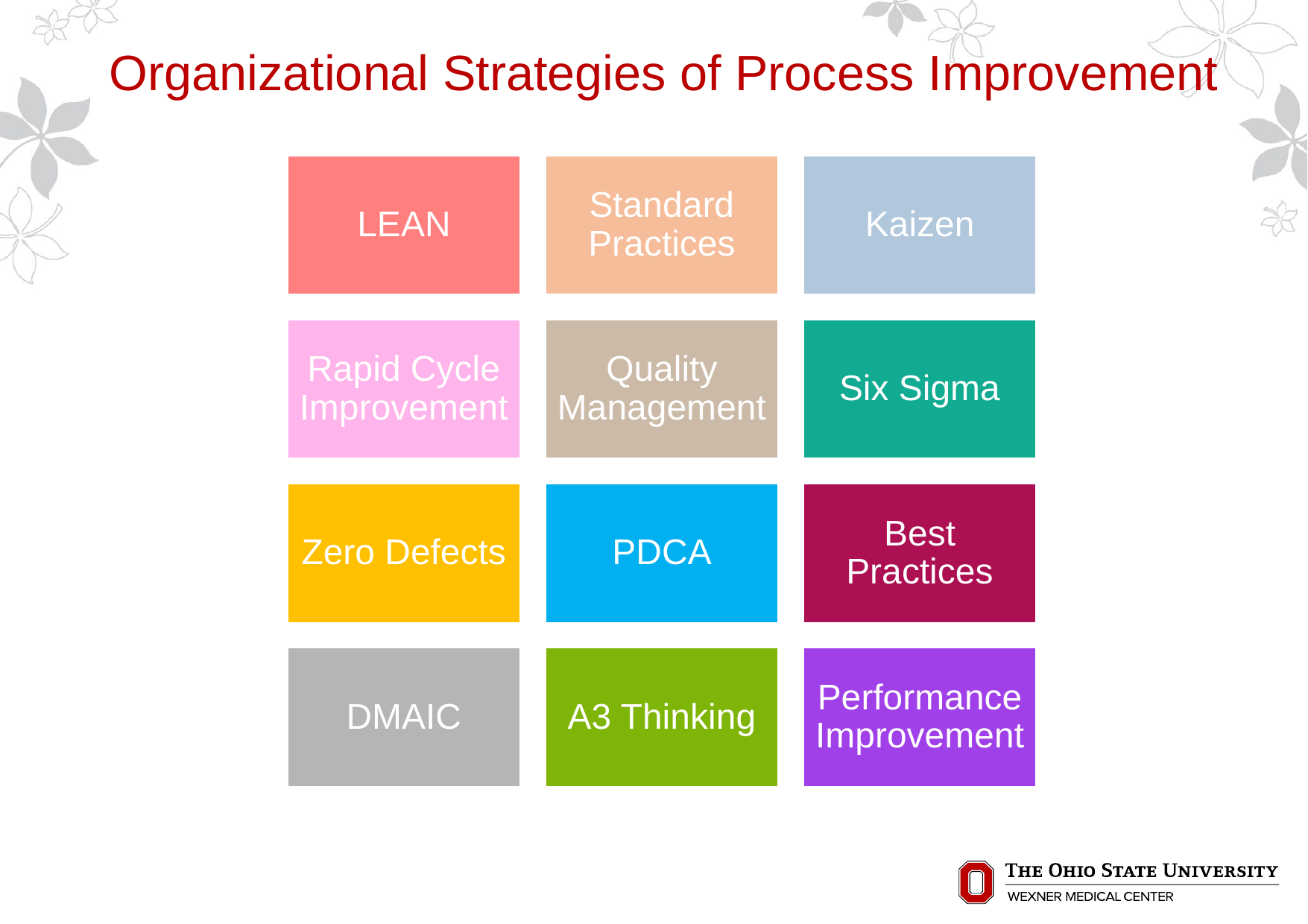

# Organizational Strategies of Process Improvement

## Slide 49
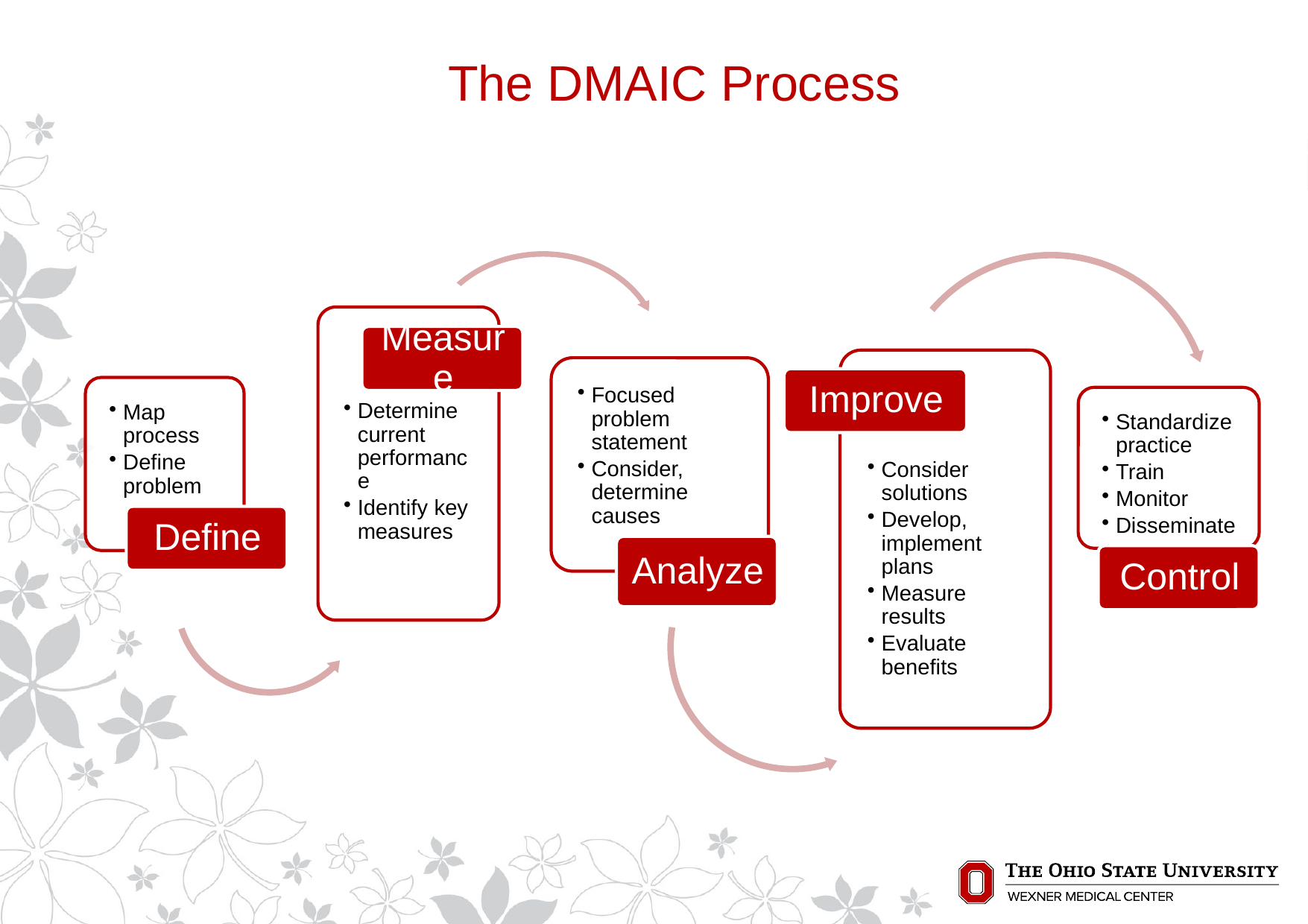

# The DMAIC Process

## Slide 50
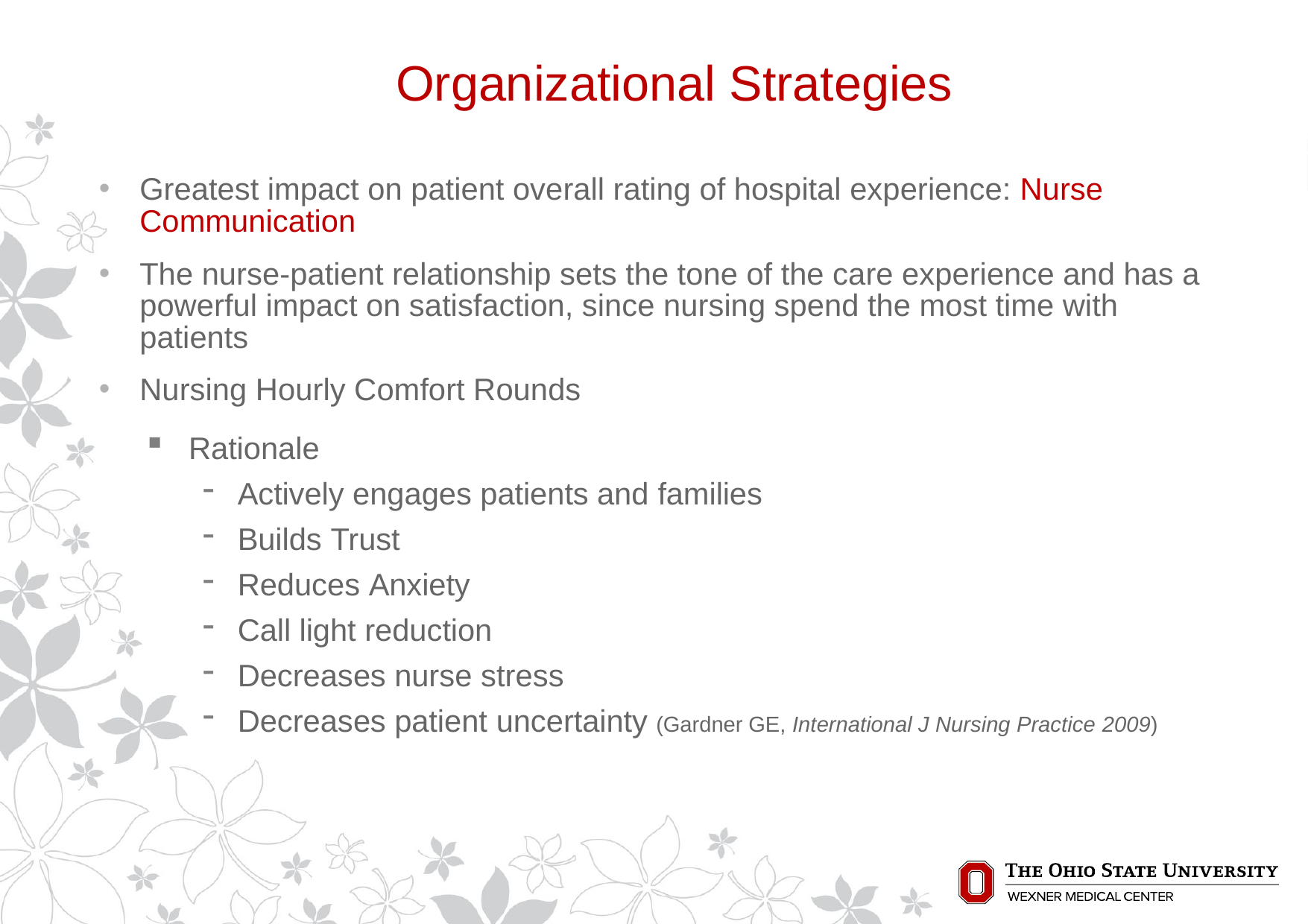

# Organizational Strategies
Greatest impact on patient overall rating of hospital experience: Nurse Communication
The nurse-patient relationship sets the tone of the care experience and has a powerful impact on satisfaction, since nursing spend the most time with patients
Nursing Hourly Comfort Rounds
Rationale
Actively engages patients and families
Builds Trust
Reduces Anxiety
Call light reduction
Decreases nurse stress
Decreases patient uncertainty (Gardner GE, International J Nursing Practice 2009)

## Slide 51
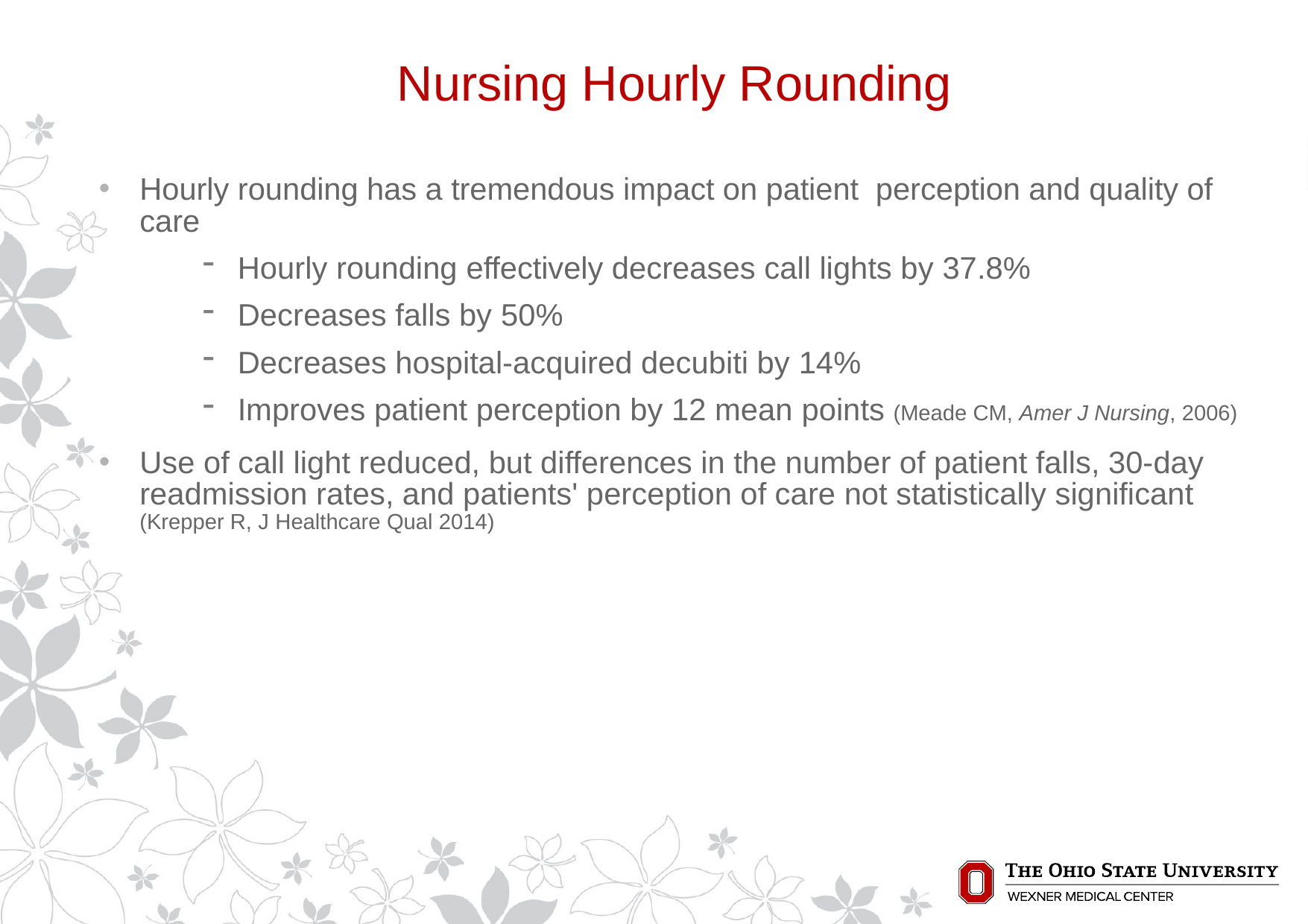

# Nursing Hourly Rounding
Hourly rounding has a tremendous impact on patient perception and quality of care
Hourly rounding effectively decreases call lights by 37.8%
Decreases falls by 50%
Decreases hospital-acquired decubiti by 14%
Improves patient perception by 12 mean points (Meade CM, Amer J Nursing, 2006)
Use of call light reduced, but differences in the number of patient falls, 30-day readmission rates, and patients' perception of care not statistically significant (Krepper R, J Healthcare Qual 2014)

## Slide 52
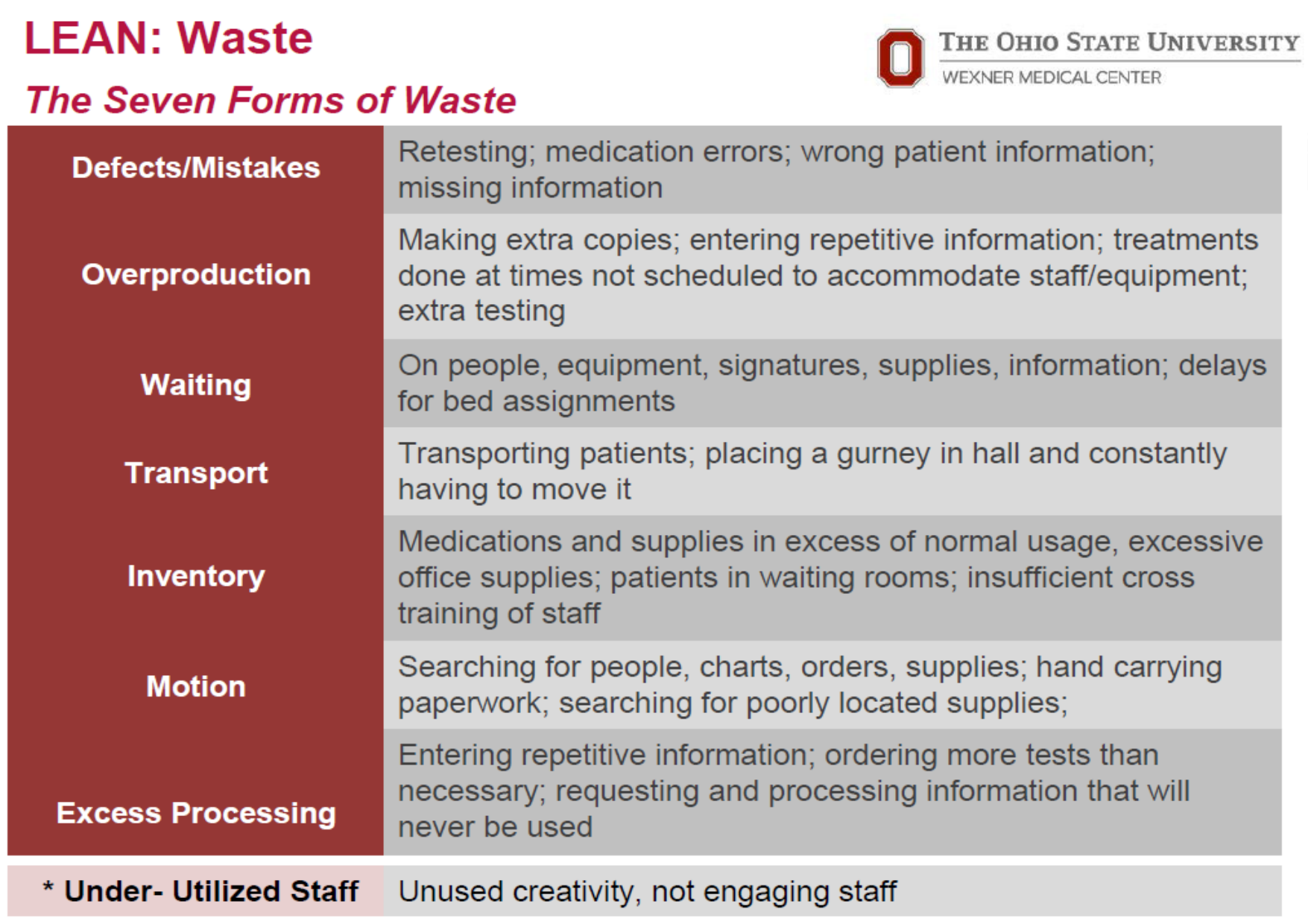

## Slide 53
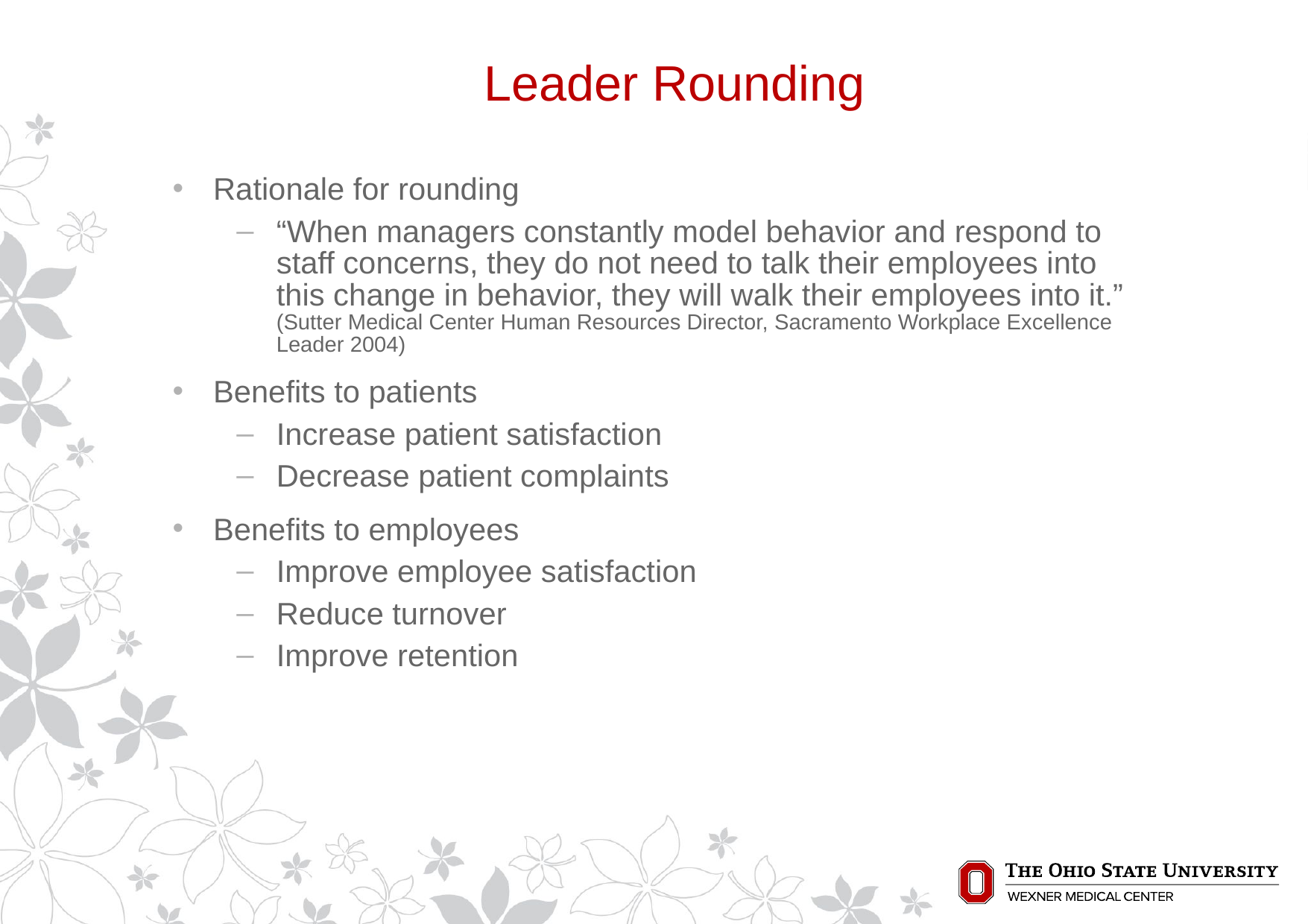

# Leader Rounding
Rationale for rounding
“When managers constantly model behavior and respond to staff concerns, they do not need to talk their employees into this change in behavior, they will walk their employees into it.” (Sutter Medical Center Human Resources Director, Sacramento Workplace Excellence Leader 2004)
Benefits to patients
Increase patient satisfaction
Decrease patient complaints
Benefits to employees
Improve employee satisfaction
Reduce turnover
Improve retention

## Slide 54
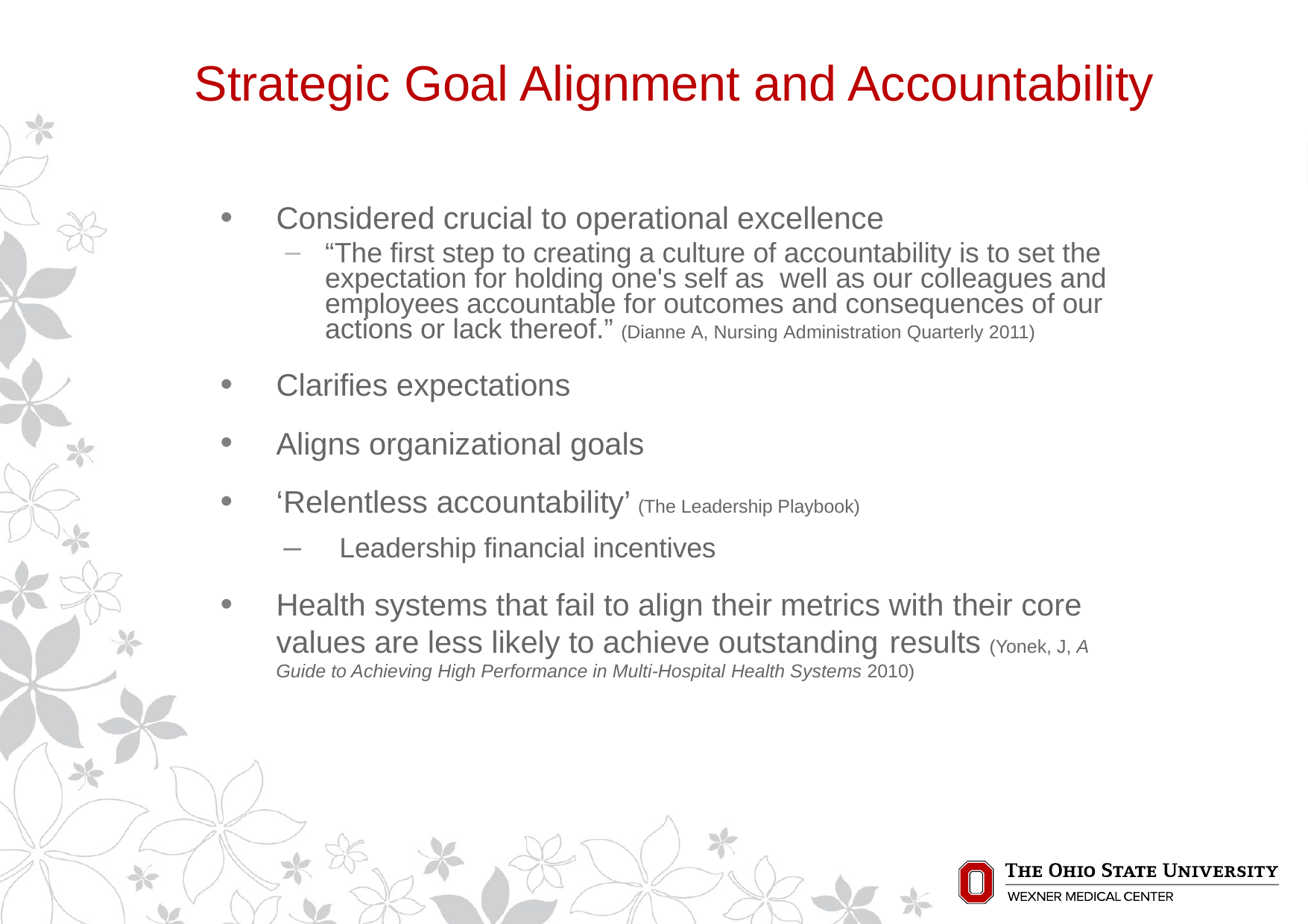

# Strategic Goal Alignment and Accountability
Considered crucial to operational excellence
“The first step to creating a culture of accountability is to set the expectation for holding one's self as well as our colleagues and employees accountable for outcomes and consequences of our actions or lack thereof.” (Dianne A, Nursing Administration Quarterly 2011)
Clarifies expectations
Aligns organizational goals
‘Relentless accountability’ (The Leadership Playbook)
Leadership financial incentives
Health systems that fail to align their metrics with their core values are less likely to achieve outstanding results (Yonek, J, A Guide to Achieving High Performance in Multi-Hospital Health Systems 2010)

## Slide 55
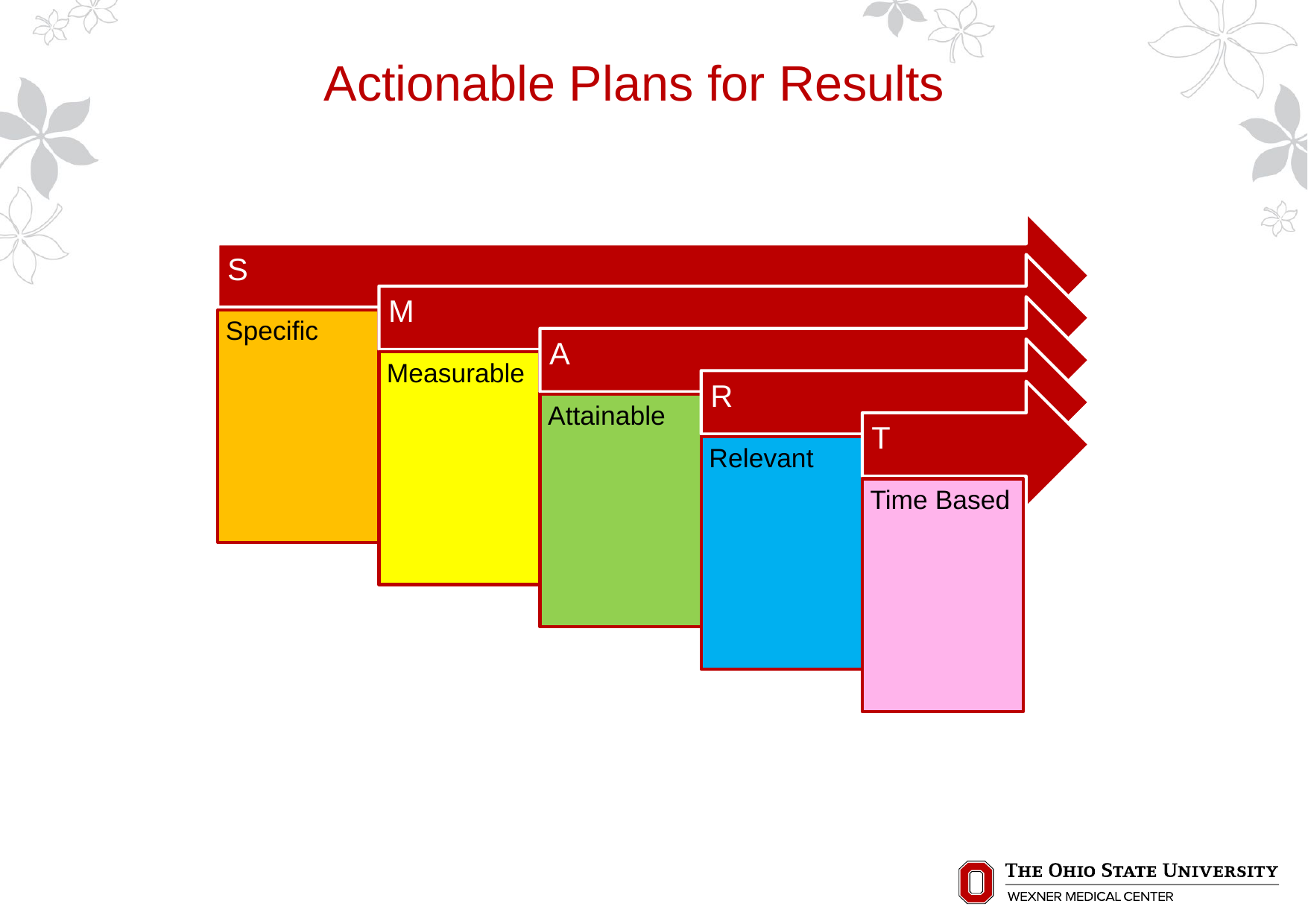

Actionable Plans for Results

## Slide 56
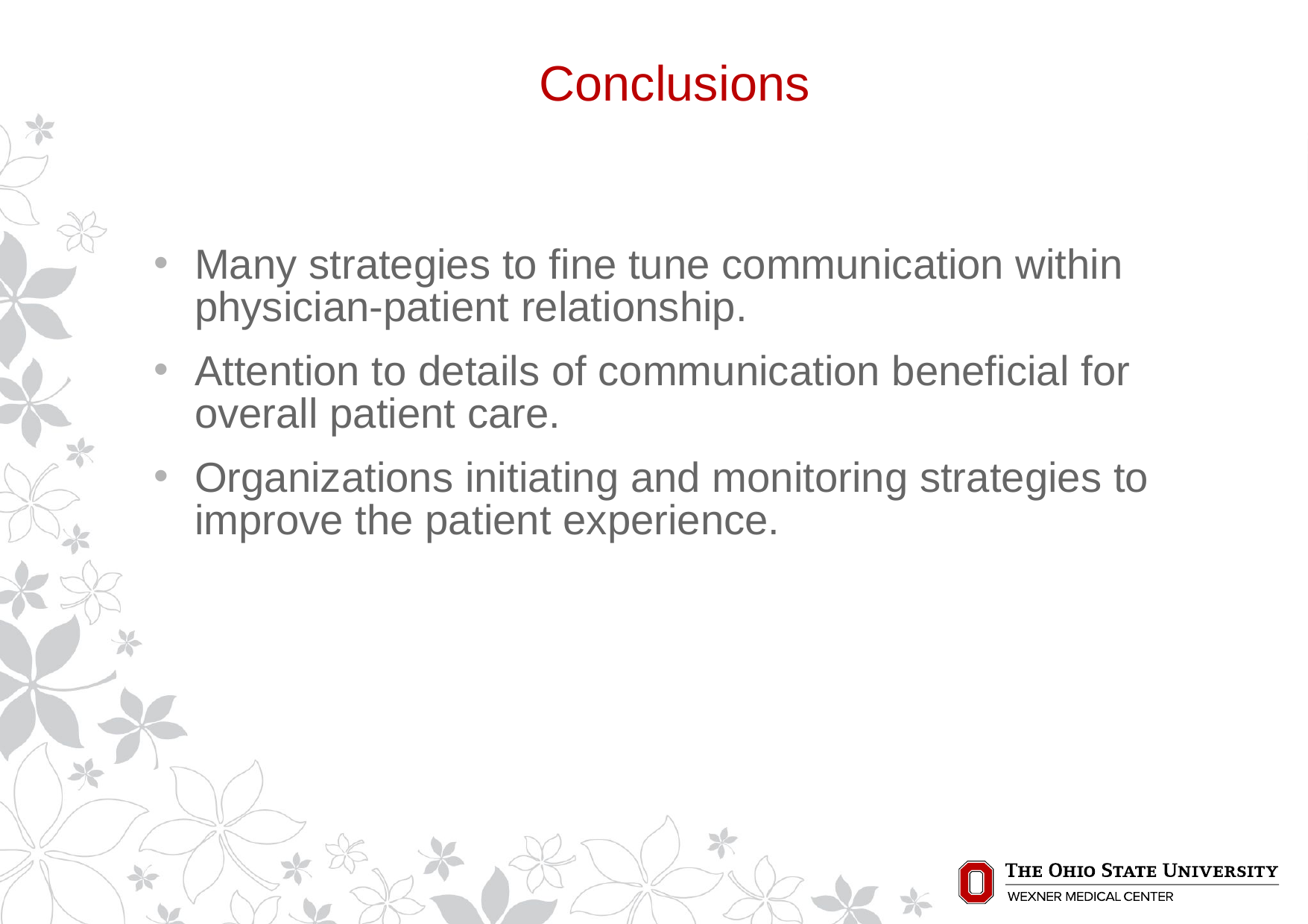

# Conclusions
Many strategies to fine tune communication within physician-patient relationship.
Attention to details of communication beneficial for overall patient care.
Organizations initiating and monitoring strategies to improve the patient experience.
