# Supplementary material for: Understanding Patient Experience: A Course for Residents
Source: MedEdPORTAL. 2017 Mar 22;13:10558. doi: 10.15766/mep_2374-8265.10558 (PMC6342057; doi:10.15766/mep_2374-8265.10558)
Supplement: Supplementary file 1 — A. Pre- and Postsession Survey.docx B. Understanding the Patient Experience Presentation.pptx C. Self-Assessment of Patient Encounters.docx D. Facilitator Questions.docx E. Patient Survey Questions.docx [file mep-13-10558-s001.zip › C. Self-Assessment of Patient Encounters.docx]

**Self-Assessment of Patient Encounters**

Place a mark in the box that describes the frequency that you perform this behavior: **Always**, **Sometimes**, or **Rarely**.

| Patient Satisfaction Behavioral Standards | Always | Sometimes | | | Rarely |
| --- | --- | --- | --- | --- | --- |
|  |  |  |  |  |  |
| I knock before entering a patient’s room. |  |  | | |  |
| I introduce myself to my patients/visitors. |  |  | | |  |
| I greet patients with a smile. |  |  | | |  |
| I shake each patient’s hand upon entry. |  |  | | |  |
| I sit down, if possible, to talk with patients. |  |  | | |  |
| I explain my role in the patient’s care. |  |  | | |  |
| I spend the first few minutes listening to the patient. |  |  | | |  |
| I acknowledge the patient’s time/wait during encounters. |  |  | | |  |
| I restate patient’s concerns to ensure common understanding. |  |  | | |  |
| I explain patient’s diagnostic considerations. |  |  | | |  |
| I explain proposed treatments. |  |  | | |  |
| I offer the patient the opportunity to ask questions. |  |  | | |  |
| I ask if there are further questions/concerns before concluding patient encounters. |  |  | | |  |
| I dress professionally. |  |  | | |  |
| I leave my personal problems out of patient care areas. |  |  | | |  |
| If I cannot help someone I take time to find someone who can. |  |  | | |  |
| I know and make an effort to learn the names of others involved in patient care. |  |  | | |  |
| I “manage up” other employees and departments every day. |  |  | | |  |
| I use phrases like “please” and “thank you”. |  |  | | |  |
| I ensure personal calls/texts do not interfere with work. |  |  | | |  |
| I attribute delays in care to others or institutional issues. |  |  | | |  |
| I confront staff about care issues in front of patients. |  |  | | |  |
| I accept responsibility by acknowledging my mistakes. |  |  | | |  |
| I put the patient first. |  |  | | |  |
| I acknowledge concerns from patients and apologize if indicated. |  |  | | |  |
| I tell patients that we are short-staffed. |  |  | | |  |
| Patient satisfaction is a priority when doing my job. |  |  | | |  |
| I am interested in improving my own performance. |  |  | | |  |
| I am accepting of rational criticism from patients/families |  |  | | |  |
| Safety is a priority when doing my job. |  |  | | |  |
| I arrive at work on time. |  |  | | |  |
| I enjoy caring for my patients and their families. |  |  | | |  |
| I believe in the mission of care delivery identified by leadership. |  |  | | |  |
| I point out problems in a positive manner. |  |  | | |  |
| I follow through on patient care issues. |  |  | | |  |
| I avoid use of negative body language. |  |  | | |  |
| I convey empathy in the care of patients. |  |  | | |  |
| I demonstrate patience, even during difficult circumstances. |  |  | | |  |
| I am efficient and utilize my time with patients constructively. |  |  | | |  |
| I demonstrate respect to everyone I encounter in the workplace. |  |  | | |  |
| I effectively represent my organization’s mission of service. |  |  | | |  |
| I have a good understanding of what represents good service. |  |  | | |  |
