# Supplementary material for: Understanding Patient Experience: A Course for Residents
Source: MedEdPORTAL. 2017 Mar 22;13:10558. doi: 10.15766/mep_2374-8265.10558 (PMC6342057; doi:10.15766/mep_2374-8265.10558)
Supplement: Supplementary file 1 — A. Pre- and Postsession Survey.docx B. Understanding the Patient Experience Presentation.pptx C. Self-Assessment of Patient Encounters.docx D. Facilitator Questions.docx E. Patient Survey Questions.docx [file mep-13-10558-s001.zip › D. Facilitator Questions.docx]

Facilitator Questions

Encourage participants to share encounters with patients and families that are and are not representative of service principles.

- What made the encounters memorable?
- Identify strengths and opportunities for growth?

Share de-identified hospital satisfaction data for learners to review.

- Discuss concerns involving the validity of data or confounding variables
  - Survey return rate
  - Location of care
  - Reason for seeking care
- Comment on complexity of domains involved in patient satisfaction
  - Food
  - Environment of care
  - Parking
  - Other factors?
- Consider trends over time (if available)
  - What are areas of improvement?
  - Are there hospital-wide efforts in place to address any concerns?
- Physician-specific areas for discussion
  - Communication
  - Time spent with patient

Optional: Provide data privately to individual physicians for review and discussion.

It may also be helpful to discuss patient feedback as a domain of practice-based learning. In other words, the analogy of using survey information to improve our patient interactions is similar to the process of evaluating treatment interventions to determine effectiveness and next course of action.
