# Supplementary material for: Understanding Patient Experience: A Course for Residents
Source: MedEdPORTAL. 2017 Mar 22;13:10558. doi: 10.15766/mep_2374-8265.10558 (PMC6342057; doi:10.15766/mep_2374-8265.10558)
Supplement: Supplementary file 1 — A. Pre- and Postsession Survey.docx B. Understanding the Patient Experience Presentation.pptx C. Self-Assessment of Patient Encounters.docx D. Facilitator Questions.docx E. Patient Survey Questions.docx [file mep-13-10558-s001.zip › E. Patient Survey Questions.docx]

Patient Experience Survey Questions

Please rate your experience for each question.

During this hospital stay or clinic visit:

1. How often did the resident physician treat you with courtesy and respect?

Always Usually Sometimes Never

1. How often did the resident physician listen carefully to you?

Always Usually Sometimes Never

1. How often did the resident physician explain things in a way you could understand?

Always Usually Sometimes Never

1. How would you describe the helpfulness of time with the resident physician?

Very Good Good Fair Poor Very Poor

1. How would you describe the resident physician’s efforts to provide you with information about your condition?

Very Good Good Fair Poor Very Poor

1. Were you given any medicine that you had not taken before? (if yes, go to question 7)

Yes No

1. How would you describe the resident physician’s efforts to provide you with information about your medication?

Very Good Good Fair Poor Very Poor

1. Please provide any additional comments about your interaction with this physician.

______________________________________________________________________________
